# Trends in US Pediatric Hospital Admissions in 2020 Compared With the Decade Before the COVID-19 Pandemic

**DOI:** 10.1001/jamanetworkopen.2020.37227

**Published:** 2021-02-12

**Authors:** Jonathan H. Pelletier, Jaskaran Rakkar, Alicia K. Au, Dana Fuhrman, Robert S. B. Clark, Christopher M. Horvat

**Affiliations:** 1 Division of Pediatric Critical Care Medicine, Department of Critical Care Medicine, UPMC Children’s Hospital of Pittsburgh, Pittsburgh, Pennsylvania; 2 Division of Health Informatics, Department of Pediatrics, UPMC Children’s Hospital of Pittsburgh, Pittsburgh, Pennsylvania

## Abstract

**Importance:**

In early 2020, the United States declared a public health emergency in response to coronavirus disease 2019 (COVID-19) and implemented a variety of social distancing measures. The association between the COVID-19 pandemic and the number of pediatric admissions is unclear.

**Objective:**

To determine the changes in patterns of pediatric admissions in 2020 compared with the prior decade.

**Design, Setting, and Participants:**

This cross-sectional study included 49 US hospitals contributing to the Pediatric Health Information Systems database. Inpatient admissions were transformed into time-series data, and ensemble forecasting models were generated to analyze admissions across a range of diagnoses in 2020 compared with previous years. The setting was inpatient admissions. All patients discharged between January 1, 2010, and June 30, 2020, from an inpatient hospital encounter were included.

**Main Outcomes and Measures:**

Number of hospital admissions by primary diagnosis for each encounter.

**Results:**

Of 5 424 688 inpatient encounters among 3 372 839 patients (median [interquartile range] age, 5.1 [0.7-13.3] years; 2 823 748 [52.1%] boys; 3 171 224 [58.5%] White individuals) at 49 hospitals, 213 571 (3.9%) were between January 1, 2020, and June 30, 2020. There was a decrease in the number of admissions beginning in March 2020 compared with the period from 2010 to 2019. At the nadir, admissions in April 2020 were reduced 45.4% compared with prior years (23 798 in April 2020 compared with a median [interquartile range] of 43 550 [42 110-43 946] in April 2010-2019). Inflation-adjusted hospital charges decreased 27.7% in the second quarter of 2020 compared with prior years ($4 327 580 511 in 2020 compared with a median [interquartile range] of $5 983 142 102 [$5 762 690 022-$6 324 978 456] in 2010-2019). Seasonal patterns were evident between 2010 and 2019 for a variety of common pediatric conditions, including asthma, atrial septal defects, bronchiolitis, diabetic ketoacidosis, Kawasaki syndrome, mental health admissions, and trauma. Ensemble models were able to discern seasonal patterns in admission diagnoses and accurately predicted admission rates from July 2019 until December 2019 but not from January 2020 to June 2020. All diagnoses except for birth decreased below the model 95% CIs between January 2020 and June 2020.

**Conclusions and Relevance:**

In this cross-sectional study, pediatric admissions to US hospitals decreased in 2020 across an array of pediatric conditions. Although some conditions may have decreased in incidence, others may represent unmet needs in pediatric care during the COVID-19 pandemic.

## Key Points

**Question:**

How have pediatric inpatient admission volumes changed in January to June 2020 compared with prior years?

**Findings:**

This cross-sectional study of 5 424 688 admissions at 49 hospitals in the Pediatric Health Information Systems database used ensemble forecasting models to demonstrate differences between inpatient pediatric admissions in 2020 compared with prior years. There was a maximum 45.4% reduction in admissions in 2020, associated with a 27.7% reduction in hospital charges, with significant reductions in all examined diagnoses except for birth.

**Meaning:**

In this study, inpatient pediatric admissions in 2020 were reduced across a heterogeneous range of diagnoses during the coronavirus disease 2019 pandemic.

## Teaser

This cross-sectional study examines the changes in patterns of pediatric admissions in 2020 compared with admissions in the prior decade.

## Introduction

The first laboratory-confirmed case of coronavirus disease 2019 (COVID-19) was reported in the United States in January 2020.^1^ In response, the United States declared a public health emergency on January 31, 2020, expanded airport screenings, required quarantines for travelers, and undertook public information efforts.^2,3^ Case counts and fatalities increased slowly in January and February 2020 before achieving exponential growth in March 2020.^4^ Individual states began additional mitigation strategies in the spring, including stay-at-home orders, which took effect from early March through June 2020.^5,6^ These efforts were associated with decreased population movement, COVID-19 incidence, and mortality.^6,7^ Additionally, the US Centers for Disease Control and Prevention issued recommendations for the use of face coverings on April 3, 2020, although enforcement varied by state.^3,8^ Although COVID-19 has resulted in relatively few pediatric hospitalizations,^1,9,10^ early reports indicate that it may have been associated with reduced pediatric admission rates in the United States^11^ and worldwide.^12,13^ A 2020 study from Brazil,^14^ in which regional differences contributed to peak bronchiolitis between February and August, showed a 78% to 85% reduction in hospitalization in infants younger than 1 year for acute bronchiolitis in 2020 compared with previous years.

Many pediatric diagnoses warranting inpatient admission exhibit seasonal variation; prior work has characterized winter predominance in bronchiolitis,^15,16^ pneumonia,^16,17^ and Kawasaki syndrome^18^; autumn predominance in asthma^16,19,20^; and summer predominance in trauma.^21^ These fluctuations in disease pattern are likely influenced by population susceptibility and behavior as well as various environmental factors. However, to our knowledge, the seasonal patterns in other pediatric admissions remain unexplored. The present study is a retrospective analysis of the Pediatric Health Information Systems (PHIS) database^22^ to determine the seasonal patterns of a variety of common pediatric admission diagnoses and compare data from the previous decade with 2020 admissions. Given the changes in population behavior in 2020,^6,7^ we hypothesized that pediatric admissions for a variety of diagnoses would be decreased in 2020 compared with prior years, except for admissions for birth.^23,24^

## Methods

### Study Design and Participants

This was a retrospective cross-sectional study of patients from 51 children’s hospitals across the United States participating in PHIS, an online, quality-controlled, anonymized, administrative database maintained by the Children’s Hospital Association.^22,25^ To ensure that changes in admission rates over time were reflective of changes in caseload, rather than expansion of the database by inclusion of new hospitals, the present study was limited to the 49 hospitals that have been providing data since 2010. Patients were eligible for inclusion if they were discharged between January 1, 2010, and June 30, 2020. There were no exclusion criteria. Patients were filtered according to their primary admission diagnoses based on *International Classification of Diseases, Ninth Revision *(*ICD-9*) or *ICD-10* codes, depending on whether their admission was before or after October 1, 2015. Selected diagnoses were determined according to the percentage of total admissions in PHIS, disease pathophysiology, and seasonal distribution between 2010 and 2019. The full list of included *ICD* codes for each diagnosis group is included in eTable 1 in the Supplement. Outcomes included the number of monthly and annual admissions over time. This study was granted exemption by the University of Pittsburgh institutional review board; because the data were anonymous, informed consent could not be obtained. This manuscript follows the Strengthening the Reporting of Observational Studies in Epidemiology (STROBE) reporting guideline for cross-sectional studies.^26^

### Statistical Analysis

Encounter-level clinical and administrative data were extracted from PHIS (admission and discharge dates, age, sex, race, ethnicity, diagnostic codes, insurance status, hospital length of stay, intensive care unit length of stay, complex chronic conditions,^27^ mechanical ventilation, abstracted charges, and cost). Admissions were described with summary statistics. Hospital charges were adjusted by the Centers for Medicare & Medicaid Services wage/price index according to hospital zip code in PHIS.^22^ Charge-over-time analyses were adjusted for the quarterly gross domestic product, provided by the Bureau of Economic Analysis and expressed in quarter 1, 2010, dollars.^28^ Admission dates were grouped into month and year. Admission numbers were transformed into time series, databased on date of admission, and displayed graphically. Seasonality testing was performed with the Webel and Ollech method.^29^ Seasonal trends in admission rates between 2010 and 2019 were displayed with locally estimated scatterplot smoothing. For visual comparison, the number of admissions in 2020 was displayed against the aforementioned models. For each diagnosis group, ensemble machine-learning forecasting models were created with autoregressive integrated moving average,^30^ neural network,^31^ and locally estimated scatterplot smoothing algorithms,^32^ each weighted on time-series cross-validation.^33^ Time-series data were arranged such that each model was trained on data from January 1, 2010, until June 30, 2019. Model forecasts were tested against actual admission numbers from July 1, 2019, until June 30, 2020. Model predictions were assessed with mean absolute percentage error,^33^ and the error rates for July 2019 to December 2019 were compared against those for January 2020 to June 2020. To quantify whether social distancing had a statistically significant association with pediatric admission rates, the actual admission rate in 2019 vs 2020 was compared against the ensemble algorithm’s 95% CI.^33^ We conducted 2 exploratory analyses by repeating the aforementioned steps for patients with any diagnostic code for acute respiratory failure and for in-hospital mortality. All statistical analyses were performed with R Studio version 1.3.1073 and R versions 4.0.2 and 4.0.3 (R Project for Statistical Computing) with the following packages: Metrics, forecastHybrid, thief, forecast, cowplot, lubridate, forcats, stringr, dplyr, purrr, readr, tidyr, tibble, ggplot2, and tidyverse.^34^ An α value of .05 was set as the threshold for statistical significance, and all tests were 2-tailed. The code used to create the manuscript is publicly available.^35^

## Results

### Demographic Characteristics

There were 49 hospitals with admission data in PHIS between January 1, 2010, and June 30, 2020. There were 5 424 688 encounters among 3 372 839 patients beginning in 2010, of which 213 571 encounters (3.9%) were between January 1, 2020, and June 30, 2020. As shown in the Table, the cohort had a median (interquartile range [IQR]) age of 5.1 (0.7-13.3) years and included 2 823 748 (52.1%) boys, 3 171 224 (58.5%) White individuals, 994 915 (18.3%) Black individuals, and 1 111 025 (20.5%) Hispanic individuals. A total of 2 977 372 patients (54.9%) had government insurance, and 2 481 647 (45.7%) had a preexisting complex chronic condition. As shown in eFigure 1 in the Supplement, the median (IQR) length of stay was 3 (2-5) days. A total of 186 238 admissions (3.4%) had a length of stay of 30 days or more. Overall survival to discharge was 99.2% (5 380 125 encounters). There were 16 selected diagnoses, encompassing 1 446 376 encounters (26.7%). The number of encounters for each selected diagnosis is shown in eTable 2 in the Supplement; birth, bronchiolitis, and trauma were the most common diagnoses.

**Table.** Demographic Characteristics

Characteristic	All PHIS inpatient encounters, No. (%) (N = 5 424 688)
Unique patients, No.	3 372 839
Age, median (IQR), y	5.1 (0.7-13.3)
Sex	
Male	2 823 748 (52.1)
Female	2 599 893(47.9)
Missing or unknown	1047 (<0.1)
Race	
White	3 171 224 (58.5)
Black	994 915 (18.3)
Asian	213 026 (3.9)
American Indian	49 826 (0.9)
Pacific Islander	29 938 (0.6)
Other	792 834 (14.6)
Ethnicity	
Hispanic	1 111 025 (20.5)
Insurance	
Commercial	2 196 624 (40.5)
Government	2 977 372 (54.9)
Other	250 692 (4.6)
Complex chronic conditions	
Any	2 481 647 (45.7)
Cardiovascular	544 375 (10.0)
Gastrointestinal	663 338 (12.2)
Hematologic or immunologic	360 238 (6.6)
Malignant neoplasm	475 279 (8.8)
Metabolic	315 349 (5.8)
Neurologic	608 740 (11.2)
Genetic	395 052 (7.3)
Premature or neonatal	179 729 (3.3)
Renal	292 633 (5.4)
Respiratory	280 044 (5.2)
Technology dependent	820 083 (15.1)
Transplant recipient	90 406 (1.7)
Mental health disorder	1 021 623 (18.8)
Admission characteristics	
Hospital length of stay, median (IQR), d	3 (2-5)
Admitted to ICU	927 942 (17.1)
Received mechanical ventilation	522 725 (9.6)
Received ECMO	14 691 (0.3)
Admission charges, median (IQR), $	
Abstracted	25 813 (13 376-56 403)
CMS adjusted abstracted	25 095 (13 109-54 272)
Survival	
Survived to discharge	5 380 125 (99.2)

Abbreviations: CMS, Centers for Medicare & Medicaid Services; ECMO, extracorporeal circulation membrane oxygenation; ICU, intensive care unit; IQR, interquartile range; PHIS, Pediatric Health Information Systems.

### Seasonal Patterns in PHIS Admissions

**Figure 1.**
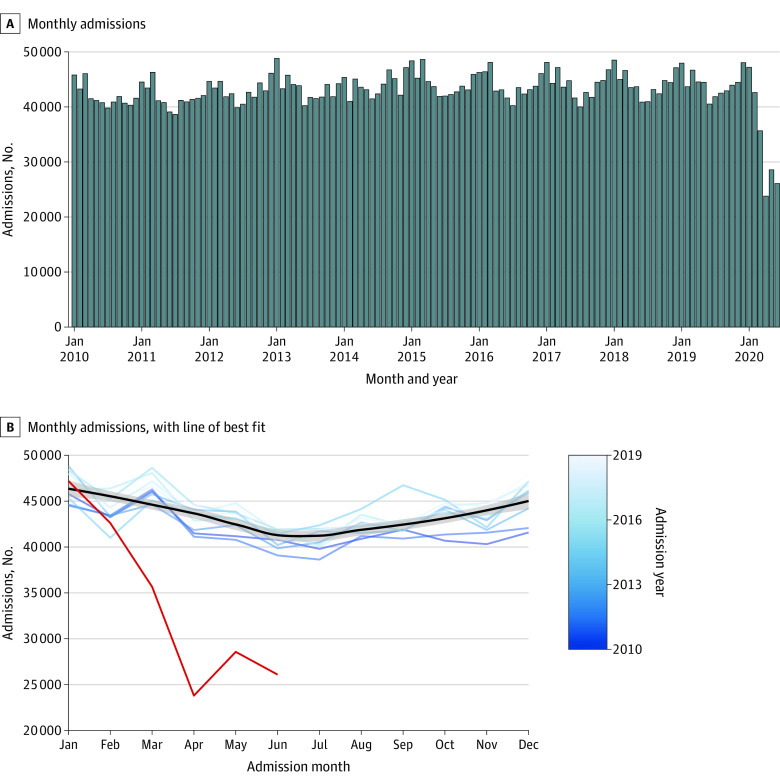
Monthly Admissions in Pediatric Health Information Systems A, Number of monthly admissions in Pediatric Health Information Systems between January 2010 and June 2020, represented as a bar chart. B, Number of monthly admissions between January 2010 and June 2020 represented as a line chart. Each blue line represents an admission year between 2010 and 2019, with progressively lighter shades of blue representing later years. The black line represents the line of best fit for the data using locally estimated scatterplot smoothing. The gray bar represents the 95% CI of the regression line. The red line shows the number of monthly admissions from January through June 2020.

Overall admissions in PHIS between 2010 and 2019 are shown in Figure 1. There was an increase in the total number of admissions in winter months compared with summer months between 2010 and 2019. There were significantly fewer admissions in July vs January in 2010 to 2019 (median [IQR], 40 726 [40 063-41 839] vs 47 116 [45 472-48 298]; *P* < .001). As shown in eFigure 2 in the Supplement, there was a significantly lower median (IQR) number of intensive care unit admissions and admissions requiring mechanical ventilation in July vs January 2010 to 2019 (7097 [6754-7483] vs 8002 [7222-8501]; *P* = .04 and 4140 [3962-4234] vs 4525 [4213-4776]; *P* = .02, respectively). There was a decrease in the number of admissions beginning in March 2020 compared with the period from 2010 to 2019, with a nadir in April 2020 at 23 798 compared with a median (IQR) of 43 550 (42 110-43 946) in April 2010 to 2019. Admissions increased in May and June of 2020 but remained below the level of previous years, with 26 089 in June 2020 compared with a median (IQR) of 40 824 (40 302-41 583) in June 2010 to 2019.

### Seasonal Patterns in Selected Diagnoses

**Figure 2.**
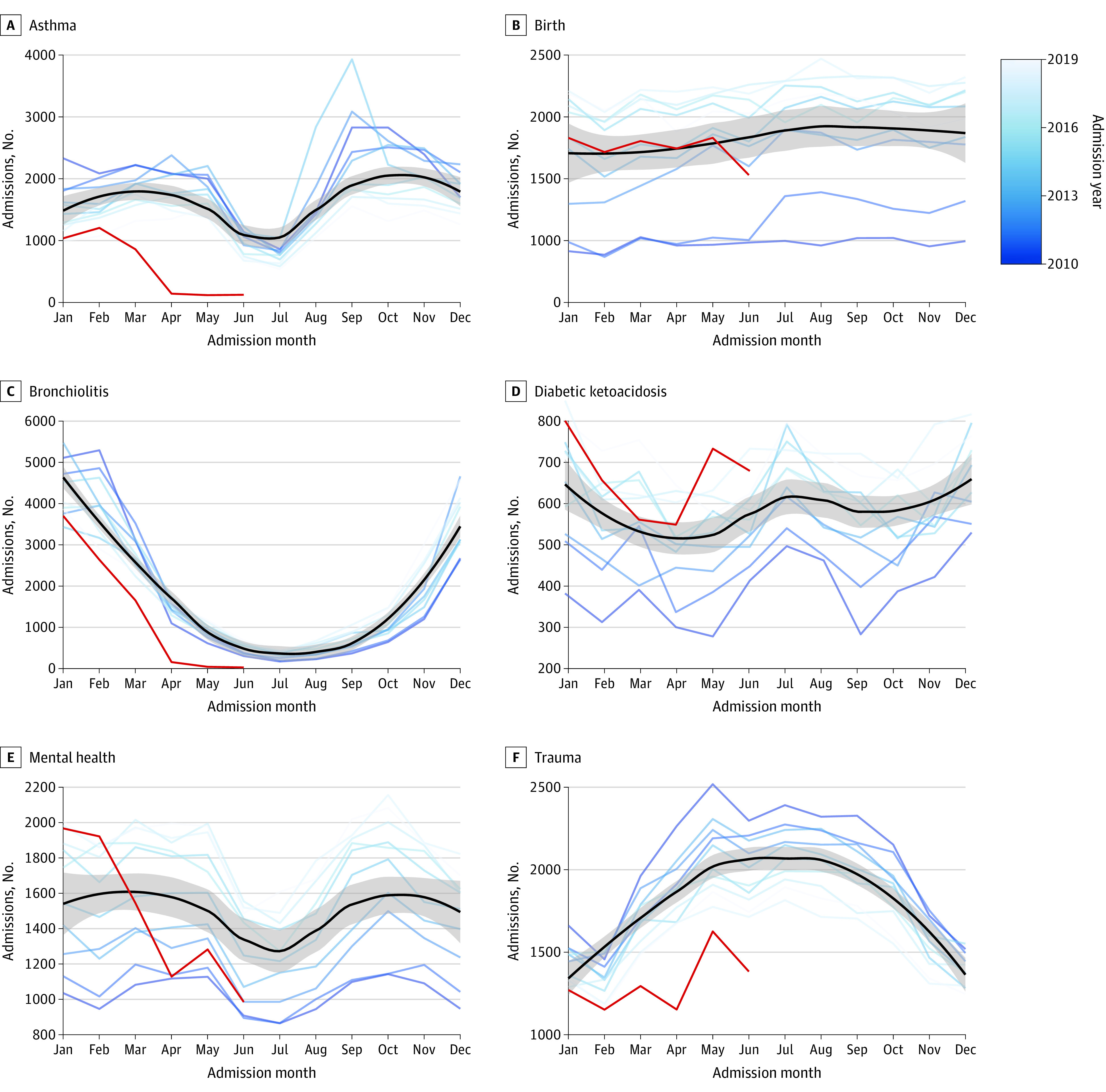
Diagnosis-Specific Monthly Admissions in Pediatric Health Information Systems Each panel shows the number of monthly admissions for a specified diagnosis between January 2010 and June 2020. Each blue line represents an admission year between 2010 and 2019, with progressively lighter shades of blue representing later years. The black line represents the line of best fit for the data using locally estimated scatterplot smoothing. The gray bar represents the 95% CI of the regression line. The red line shows the number of monthly admissions from January through June 2020.

Admissions by diagnoses are shown in Figure 2 and eFigure 3 in the Supplement. Several distinct seasonal patterns were noted. Admissions for bronchiolitis, dehydration, Kawasaki syndrome, *Streptococcus pneumoniae*, and sepsis exhibited significant winter predominance. Because bronchiolitis coding changed in *ICD-10 *to include separate diagnostic codes for respiratory syncytial virus and human metapneumovirus, the seasonality of individual pathogens is shown in eFigure 4 in the Supplement for 2016 to 2019. Admissions for asthma and mental health disorders showed significant fall and spring predominance, associated with the school-year calendar. Admissions for diabetic ketoacidosis showed significant summer and winter predominance, inversely associated with the school-year calendar in children aged 5 years and older, as shown in eFigure 5 in the Supplement. Admissions for appendicitis, atrial septal defects, birth, hypoplastic left heart syndrome, tetralogy of Fallot, and trauma showed significant summer predominance. In the case of hypoplastic left heart syndrome, summer predominance was associated with admissions in children older than 1 year, as shown in eFigure 6 in the Supplement. Admissions for cardiac arrest and coarctation of the aorta did not meet criteria for significant seasonal variation.

### Changes in Seasonal Patterns During the COVID-19 Pandemic

**Figure 3.**
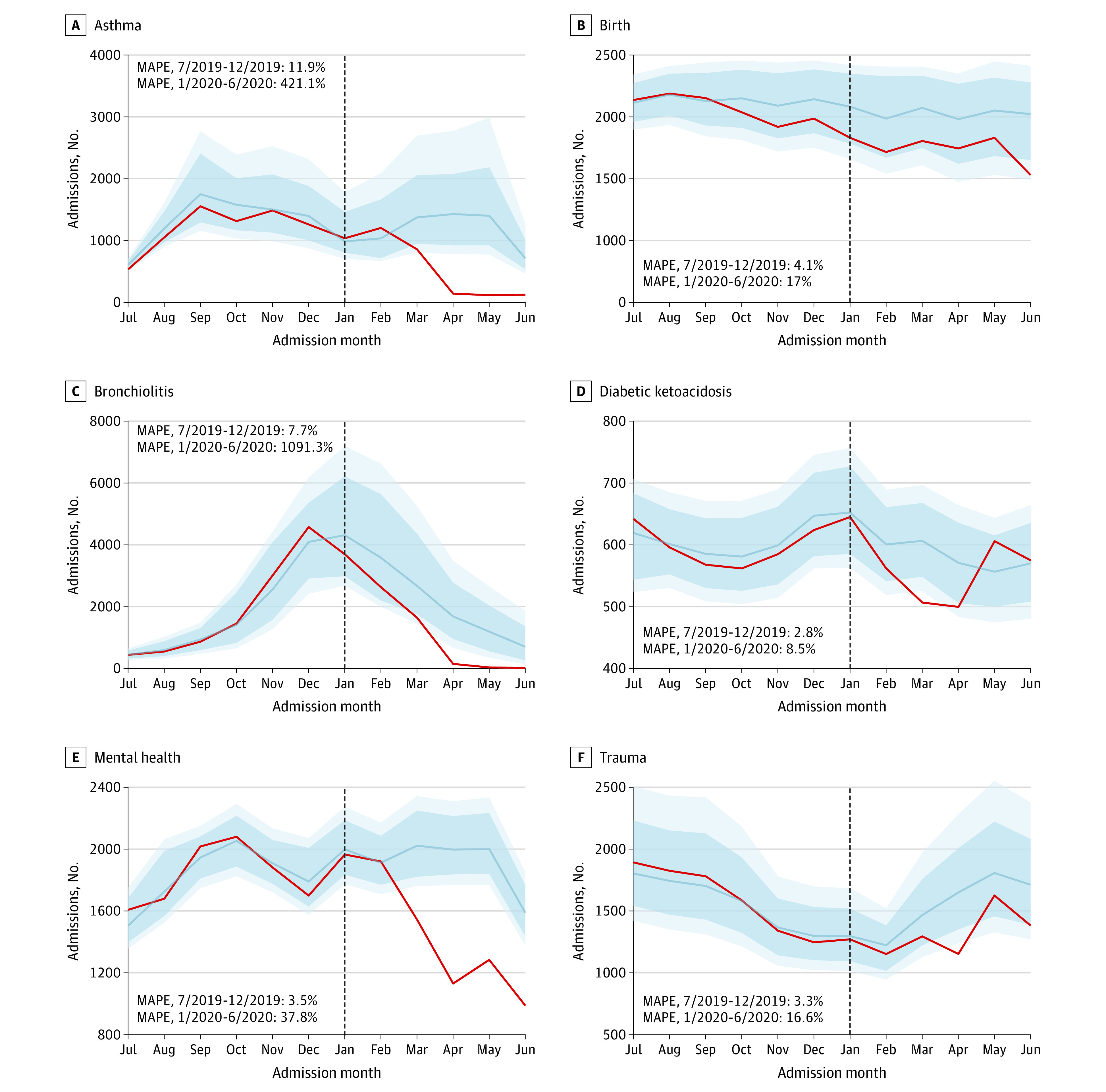
Ensemble Forecasts Each panel shows the estimated number of monthly admissions for a specified diagnosis between January 2010 and June 2020, according to ensemble forecasting models including autoregressive integrated moving average, neural network, and locally estimated scatterplot smoothing algorithms weighted on time-series cross-validation. All models were trained on data from January 2010 through June 2019, and the testing window for each model is shown. The blue line is the model estimate, and the dark and light blue shaded regions are the model 80% and 95% CIs, respectively. The red line is the actual number of admissions for each month. The vertical line represents January 2020. MAPE indicates mean absolute percentage error.

There were deviations from previous seasonal patterns in 2020, as shown in Figure 2 (denoted in red). Admissions for bronchiolitis and *S pneumoniae* reached previous summer rates by April 2020. Admissions for trauma did not exhibit a typical increase in spring 2020. Admissions for appendicitis, atrial septal defects, asthma, coarctation of the aorta, dehydration, hypoplastic left heart syndrome, Kawasaki syndrome, mental health conditions, sepsis, and tetralogy of Fallot decreased below expected levels. Birth rates were unaffected. Changes in seasonal patterns identified with ensemble time-series forecasting models are shown in Figure 3 and eFigure 7 in the Supplement. Overall, models accurately predicted admission rates from July until December 2019 but not from January to June 2020 (mean absolute percentage error range, 3% to 21.2% vs 8.5% to 1111.7%, respectively; *P* = .06). A total of 70 of 72 monthly admission (97.2%) rates between July and December 2019 fell within the 95% CI of the models. The admission rates for atrial septal defect and hypoplastic left heart syndrome were below the 95% CI predicted by the models in August and December 2019, respectively. All other conditions remained within the model 95% CIs between July and December 2019. Admissions for birth remained within the model 95% CI between January and June 2020. Every other condition decreased below the model 95% CI between January and June 2020.

### Hospital Charges

Overall, 5 377 745 of 5 424 688 encounters (99.1%) among 3 356 656 patients had hospital charge data available. The quarterly total hospital charges, adjusted for Centers for Medicare & Medicaid Services wage/price index and quarterly gross domestic product, are shown in eFigure 8 in the Supplement. The per-admission charges with the same adjustments are shown in eFigure 9 in the Supplement. The total hospital charges in quarter 1 of 2020 were $6 024 735 078 compared with a median (IQR) of $6 229 556 859 ($5 951 083 755-$6 551 134 282) between 2010 and 2019, representing a 3.3% decrease. The total hospital charges in quarter 2 of 2020 were $4 327 580 511 compared with a median (IQR) of $5 983 142 102 ($5 762 690 022-$5 991 085 056) between 2010 and 2019, representing a 27.7% decrease. However, the median (IQR) per-admission charge in 2020 was $24 358 ($12 631-$51 740) compared with a median (IQR) of $20 352 ($10 781-$43 604) in 2010 to 2019 (*P* < .001).

### Exploratory Analyses

Diagnoses including any cause of acute respiratory failure displayed significant winter predominance. In-hospital mortality did not meet criteria for significant seasonal variation. Seasonal trends and machine-learning models for respiratory failure and in-hospital mortality are shown in eFigure 10 in the Supplement. There were annual increases in admissions for acute respiratory failure from 2010 through February 2020, with a decline in spring 2020. There were 3517 admissions for acute respiratory failure in December 2019 compared with 2143 admissions in December 2018, and 2894 in January 2020 compared with 2050 in January 2019. Acute respiratory failure decreased below the model 95% CI beginning in April 2020. There were no significant changes in admissions with in-hospital mortality between 2010 and 2019. Admissions with in-hospital mortality decreased below the model 95% CI beginning in March 2020.

## Discussion

To our knowledge, the present study is the first to use a large database to model pediatric admission volumes during the COVID-19 pandemic for various diagnoses compared with prior years. Using this approach, we identified seasonal patterns in a diverse group of pediatric conditions. Before 2020, pediatric admissions overall displayed winter predominance, associated with an increase in infectious respiratory conditions, such as bronchiolitis and pneumonia. Summer-predominant conditions included trauma and admissions for semielective surgical conditions (eg, atrial septal defects, hypoplastic left heart syndrome in children aged >1 year). The present study is in agreement with several prior studies documenting seasonality in bronchiolitis,^15,16^ pneumonia,^16,17^ asthma,^16,19,20^ Kawasaki syndrome,^18^ and trauma.^21^ However, we additionally noted novel seasonality for diabetic ketoacidosis and mental health admissions and the absence of seasonality in admissions for cardiac arrest and coarctation of the aorta. The seasonality of diabetic ketoacidosis was associated with children aged 5 years or older and may be reflective of the efforts of the American Diabetes Association to incorporate school personnel in the diabetes medical management plans for these patients.^36^

Although COVID-19 has resulted in few pediatric admissions and mortalities compared with those for adults,^1,9,10^ the present study found reductions in pediatric admission rates for non–COVID-19–related diagnoses in 2020. Admissions in April 2020 were reduced 45.4% compared with prior years (23 798 in April 2020 vs a median of 43 550 in April 2010-2019). Reductions in admissions below those predicted by ensemble forecasts were present in all of the selected diagnoses except birth. There was a premature termination of winter-predominant conditions such as bronchiolitis and pneumonia and a reduction in admissions for asthma in 2020 compared with previous years. In exploratory analyses, this was coupled with a decrease in all acute respiratory failure diagnoses. Given the infectious nature of these conditions and their spread by respiratory droplets,^37,38,39^ this may have been associated with the effects of social distancing and possibly was even fortunate, given evidence of a potentially severe respiratory virus season in 2019 to 2020, with the highest number of respiratory failure admissions recorded in the past decade. A recent Australian study corroborates our findings, with more than 95% reduction in respiratory syncytial virus and influenza detection in Western Australia through winter 2020.^40^ Taken together, these studies suggest that rates of infectious pediatric respiratory conditions are potentially modifiable by changes in human behavior. Similarly, pediatric trauma admissions are often sports related,^41^ and the COVID-19 pandemic has been shown to be associated with decreased childhood physical activity; thus, reductions in trauma admissions were anticipated.^42^ However, reductions in mental health admissions are surprising, given limited literature suggesting that pandemic-related stressors may worsen mental health in both the general population and those with preexisting psychiatric conditions.^43,44^ This may represent an opportunity for changes in the delivery of mental health care.^45^

The present study cannot delineate which changes in admission rates represent decreases in incidence, hospital avoidance, or unmet care needs. One inpatient survey suggested that greater than one-third of parents delayed seeking medical care for their child because of fears surrounding COVID-19,^46^ and case series have reported delayed presentations leading to harm.^47,48^ Similarly, although admissions for repair of atrial septal defects or later-stage repairs of hypoplastic left heart syndrome may safely be deferred for a short period, it is unclear whether reductions in admission rates represent purely elective surgical delays or the possibility of decreased recognition of congenital heart disease because of decreased contact with health care; small case series of increases in sudden death in adults with congenital heart disease without contact with health care institutions during the COVID-19 pandemic have been reported.^49^ Taken together, these findings are worrisome, although not definitive, in that unmet health care needs may be accumulating in the pediatric population as a result of decreased health care interactions.

Reduced patient volumes during early 2020 have reportedly contributed to financial instability for many hospitals and health systems.^50^ The reduction in charges observed in the present study suggests a proportionate decline in operating income. Although children’s hospitals generally maintain better operating margins compared with other nonprofit hospitals, the 45.4% reduction in inpatient admissions at children’s hospitals observed during the first 6 months of 2020 and 27.7% reduction in charges observed in the second quarter of 2020 compared with prior years has an influence on financial security.^50,51^ Leaders of children’s hospitals could face unique challenges in the coming months as patient volumes eventually recover and are potentially coupled with the destabilizing financial effects of the COVID-19 pandemic.

Characterizing alterations in usual patient admission patterns aids in anticipating the potential ramifications of recent national policies and can also help in crafting responses in a variety of areas. The present study highlights the strengths of an ensemble machine-learning approach to model complex time-series data displaying a variety of seasonal relationships. As complexity in medical information has increased, there has been progressive interest in the application of machine learning to facilitate clinical decision-making. Such algorithms have been studied in a diverse array of applications, including sepsis prediction,^52^ COVID-19 prognosis,^53^ population health,^54^ and cyberbullying.^55^ In the present study, before the COVID-19 pandemic,^6,7^ ensemble time-series forecasting models accurately predicted 70 of 72 monthly pediatric admission rates (97.2%) between July 2019 and December 2019. Quantification of the seasonality of various admission types in pediatrics has applicability for hospital, intensive care unit, and ventilator capacity planning as well as trainee education. According to the present study, pediatricians and trainees assigned to inpatient wards in the winter will experience higher-than-average case burden and gain greater experience in managing pneumonia and bronchiolitis, whereas those assigned to emergency departments in the summer will achieve greater experience in the management of pediatric trauma. Such knowledge can be harnessed in the development of training curricula that support experience across the spectrum of pediatric illness.

### Limitations

This study has important limitations. As with any retrospective database analysis, admission diagnoses are vulnerable to misclassification owing to coding error. Although including greater than 5 000 000 inpatient encounters, this study is limited to 49 hospitals that consistently provided data to PHIS between January 2010 and June 2020 and may not be representative of national pediatric admission trends. Because of data availability, data from some admissions from late June 2020 are not yet available. This may have resulted in undercounting of admissions from June. However, hospitalizations for birth did not decrease below the 95% CIs for the forecasting model. It is also possible that some hospitals have been delayed in reporting admissions to PHIS because of the pandemic, although this is not a known data quality issue. Owing to data availability, only admissions between January and June 2020 were analyzed regarding the effects of social distancing. Further study is warranted as additional data become available.

## Conclusions

In this study, common pediatric admission diagnoses exhibited seasonal variation. These trends were effectively modeled with ensemble time-series forecasting algorithms. The overall number of pediatric admissions in PHIS decreased between January 2020 and June 2020. This trend held through a wide variety of diagnoses. The reduction in pediatric admissions may be representative of unmet needs in pediatric care during the COVID-19 pandemic.

## Notes

Supplement. **eFigure 1.** Length of Stay Histogram **eFigure 2.** Monthly ICU and Ventilator Admissions in PHIS **eFigure 3.** Additional Diagnosis-Specific Monthly Admissions in PHIS **eFigure 4.** Seasonality of Bronchiolitis Pathogens **eFigure 5.** Seasonality of Diabetic Ketoacidosis by Age **eFigure 6.** Seasonality of Hypoplastic Left Heart Syndrome by Age **eFigure 7.** Ensemble Forecasts for Additional Diagnoses **eFigure 8.** GDP-Adjusted Quarterly Total Hospital Charges **eFigure 9.** GDP-Adjusted Quarterly Per-Admission Hospital Charges **eFigure 10.** Seasonality of Exploratory Analyses **eTable 1.** List of Included Diagnostic Codes **eTable 2.** Number of Encounters With Included Diagnoses
