# Error in Figure

**DOI:** 10.1001/jamanetworkopen.2021.11979

**Published:** 2021-04-29

In the Original Investigation titled “Trends in US Pediatric Hospital Admissions in 2020 Compared With the Decade Before the COVID-19 Pandemic,”^1^ published February 12, 2021, there was an error in Figure 2D. The markers on the y-axis were incorrect. The article has been corrected.^1^
