# Supplementary material for: Trends in US Pediatric Hospital Admissions in 2020 Compared With the Decade Before the COVID-19 Pandemic
Source: JAMA Netw Open. 2021 Feb 12;4(2):e2037227. doi: 10.1001/jamanetworkopen.2020.37227 (PMC7881361; doi:10.1001/jamanetworkopen.2020.37227)
Supplement: Supplement. — eFigure 1. Length of Stay Histogram eFigure 2. Monthly ICU and Ventilator Admissions in PHIS eFigure 3. Additional Diagnosis-Specific Monthly Admissions in PHIS eFigure 4. Seasonality of Bronchiolitis Pathogens eFigure 5. Seasonality of Diabetic Ketoacidosis by Age eFigure 6. Seasonality of Hypoplastic Left Heart Syndrome by Age eFigure 7. Ensemble Forecasts for Additional Diagnoses eFigure 8. GDP-Adjusted Quarterly Total Hospital Charges eFigure 9. GDP-Adjusted Quarterly Per-Admission Hospital Charges eFigure 10. Seasonality of Exploratory Analyses eTable 1. List of Included Diagnostic Codes eTable 2. Number of Encounters With Included Diagnoses [file jamanetwopen-e2037227-s001.pdf]

Supplemental Online Content

Pelletier JH, Rakkar J, Au AK, Fuhrman D, Clark RSB, Horvat CM. Trends in US pediatric hospital admissions in 2020 compared with the decade before the COVID-19 pandemic. *JAMA Netw Open*. 2021;4(2):e2037227. doi:10.1001/jamanetworkopen.2020.37227

eFigure 1. Length of Stay Histogram

eFigure 2. Monthly ICU and Ventilator Admissions in PHIS

eFigure 3. Additional Diagnosis-Specific Monthly Admissions in PHIS

eFigure 4. Seasonality of Bronchiolitis Pathogens

eFigure 5. Seasonality of Diabetic Ketoacidosis by Age

eFigure 6. Seasonality of Hypoplastic Left Heart Syndrome by Age

eFigure 7. Ensemble Forecasts for Additional Diagnoses

eFigure 8. GDP-Adjusted Quarterly Total Hospital Charges

eFigure 9. GDP-Adjusted Quarterly Per-Admission Hospital Charges

eFigure 10. Seasonality of Exploratory Analyses

eTable 1. List of Included Diagnostic Codes

eTable 2. Number of Encounters With Included Diagnoses

This supplemental material has been provided by the authors to give readers additional information about their work.

© 2021 Pelletier et al. *JAMA Network Open.*

eFigure 1. Length of Stay Histogram. The x-axis shows the length of stay in days for the cohort. The y-axis shows the number of admissions.

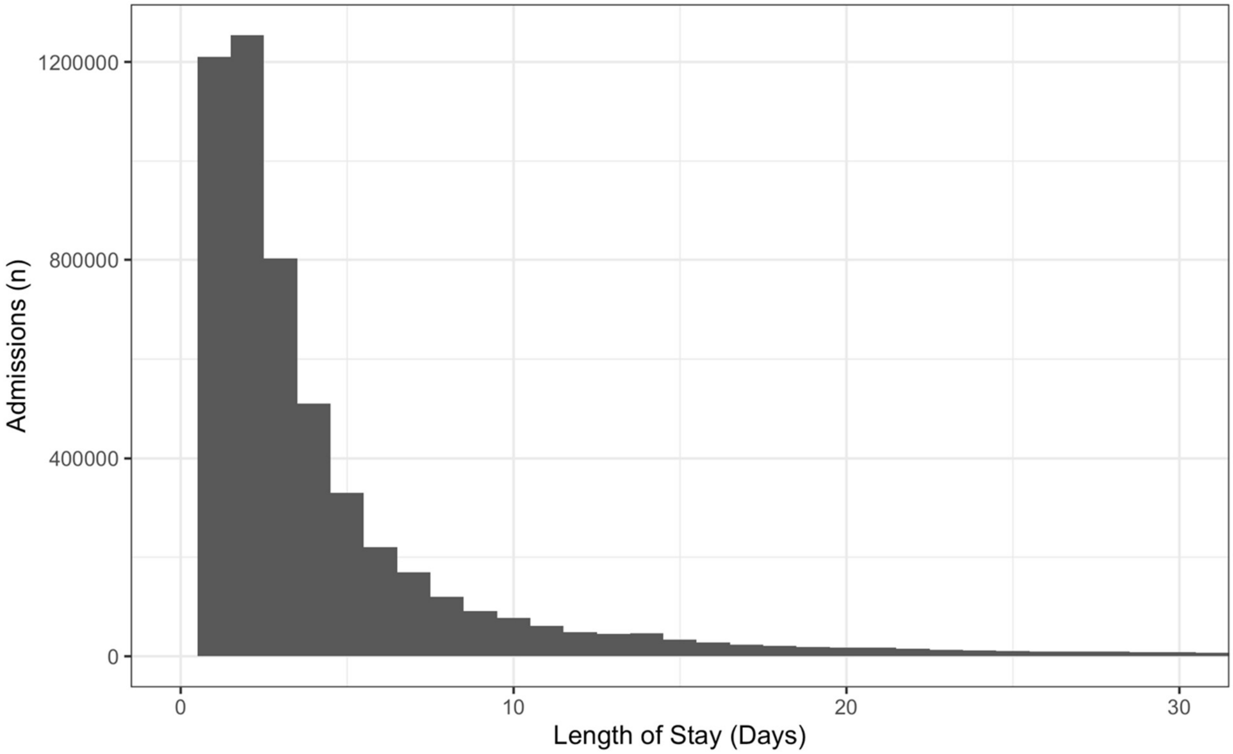

© 2021 Pelletier et al. *JAMA Network Open.*

eFigure 2. Monthly ICU and Ventilator Admissions in PHIS. Panel A shows the number of monthly ICU admissions in PHIS between 1/2010 and 6/2020 represented as a line chart. Panel B shows the number of admissions requiring mechanical ventilation for the same time period. Each blue line represents an admission year between 2010 and 2019, with progressively lighter shades of blue representing later years. The black line represents the line of best fit for the data using LOcally Estimated Scatterplot Smoothing (LOESS). The gray bar represents the 95% confidence interval of the regression line. The red line shows the number of monthly admissions from 1/2020 through 6/2020.

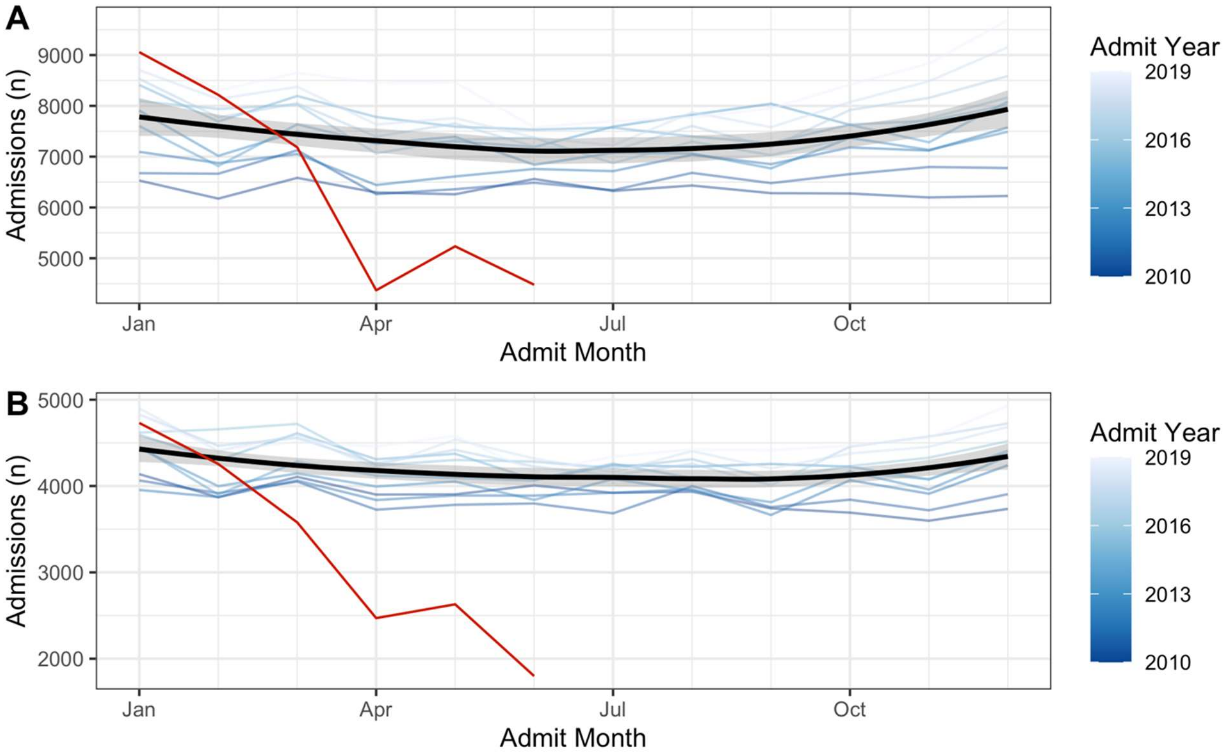

© 2021 Pelletier et al. *JAMA Network Open.*

eFigure 3. Additional Diagnosis-Specific Monthly Admissions in PHIS. Each panel shows the number of monthly admissions for a specified diagnosis between 1/2010 and 6/2020. As in Figure 1B, each blue line represents an admission year between 2010 and 2019, with progressively lighter shades of blue representing later years. The black line represents the line of best fit for the data using LOcally Estimated Scatterplot Smoothing (LOESS). The gray bar represents the 95% confidence interval of the regression line. The red line shows the number of monthly admissions from 1/2020 through 6/2020. Panel A shows additional general diagnoses, and Panel B shows cardiac diagnoses. ASD: atrial septal defect, COA: coarctation of the aorta, HLHS: hypoplastic left heart syndrome, TOF: Tetralogy of Fallot.

A

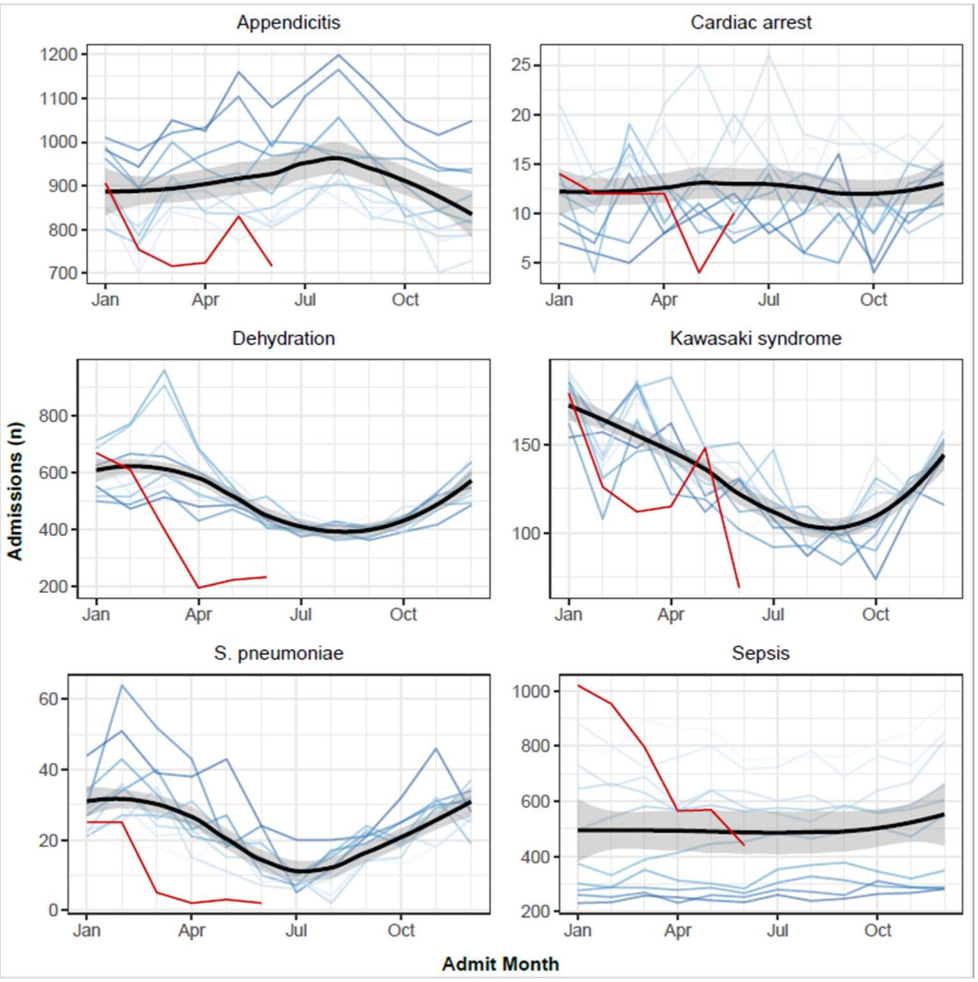

© 2021 Pelletier et al. *JAMA Network Open.*

B

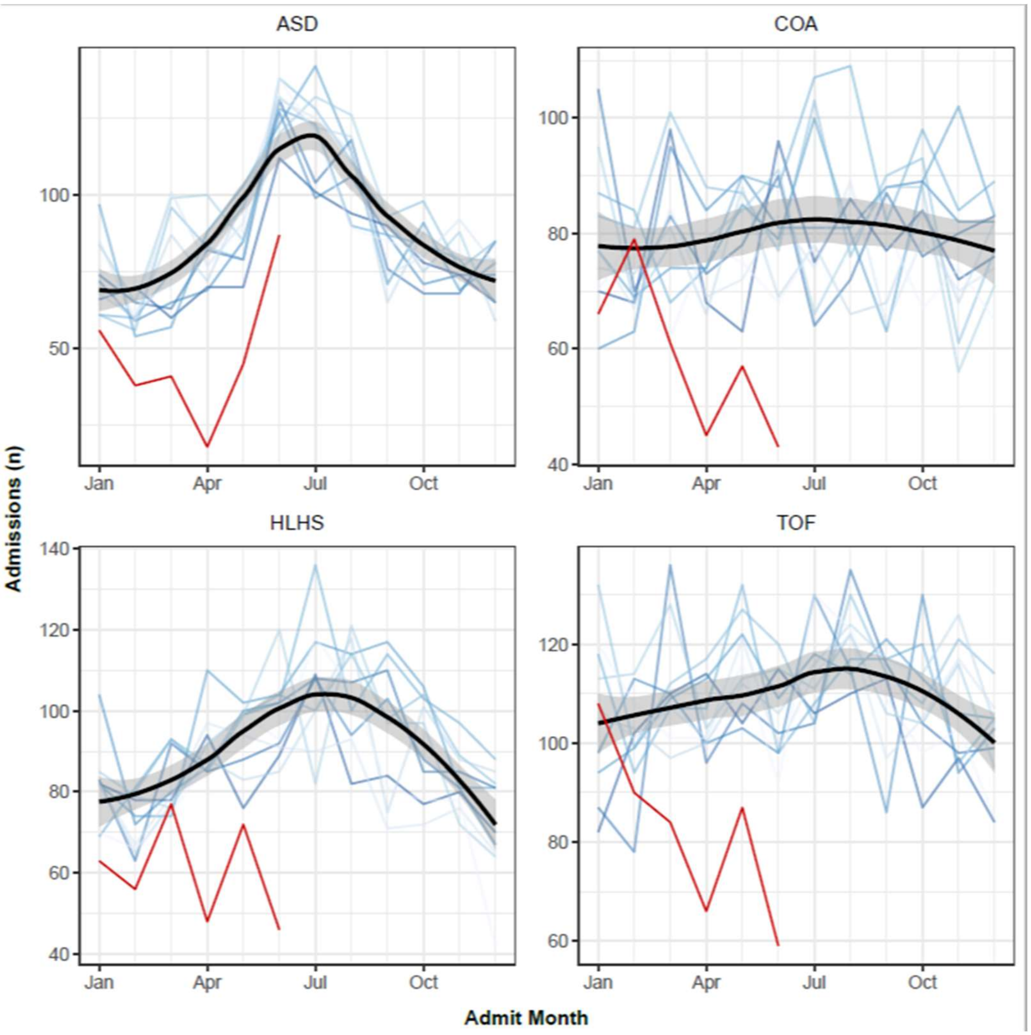

ASD
COA
HLHS
TOF
Admissions (n)
Admit Month
Jan
Apr
Jul
Oct
50
100
40
60
80
100
120
140

eFigure 4. Seasonality of Bronchiolitis Pathogens. Each panel shows the number of monthly admissions for viral bronchiolitis between 1/2016 and 12/2019 (limited to the time window during which ICD-10 was in effect). Panels are faceted according to the viral pathogen specified as the primary encounter diagnosis by ICD-10.

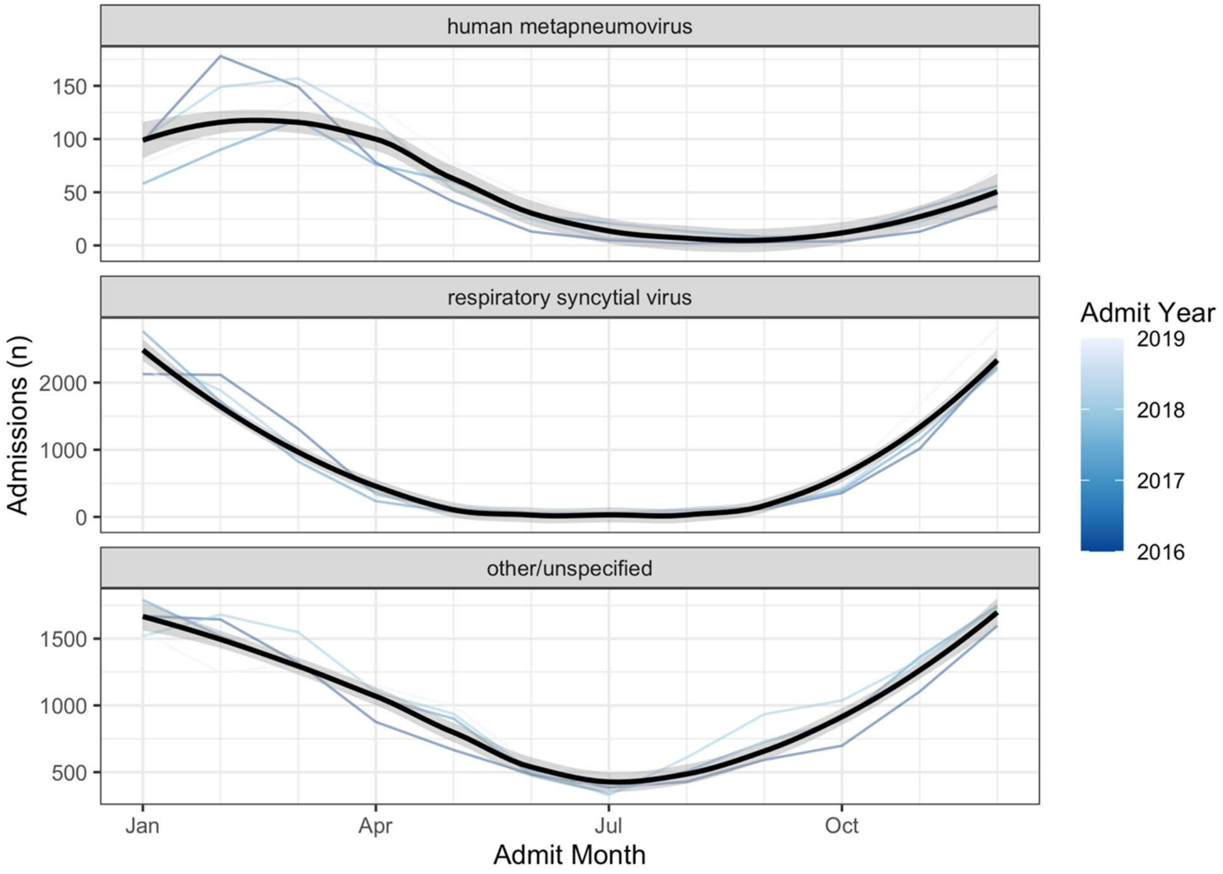

© 2021 Pelletier et al. *JAMA Network Open.*

eFigure 5. Seasonality of Diabetic Ketoacidosis by Age. Each panel shows the number of monthly admissions for hypoplastic left heart syndrome between 1/2010 and 12/2019. Panels are faceted according to the age at admission, specified in years.

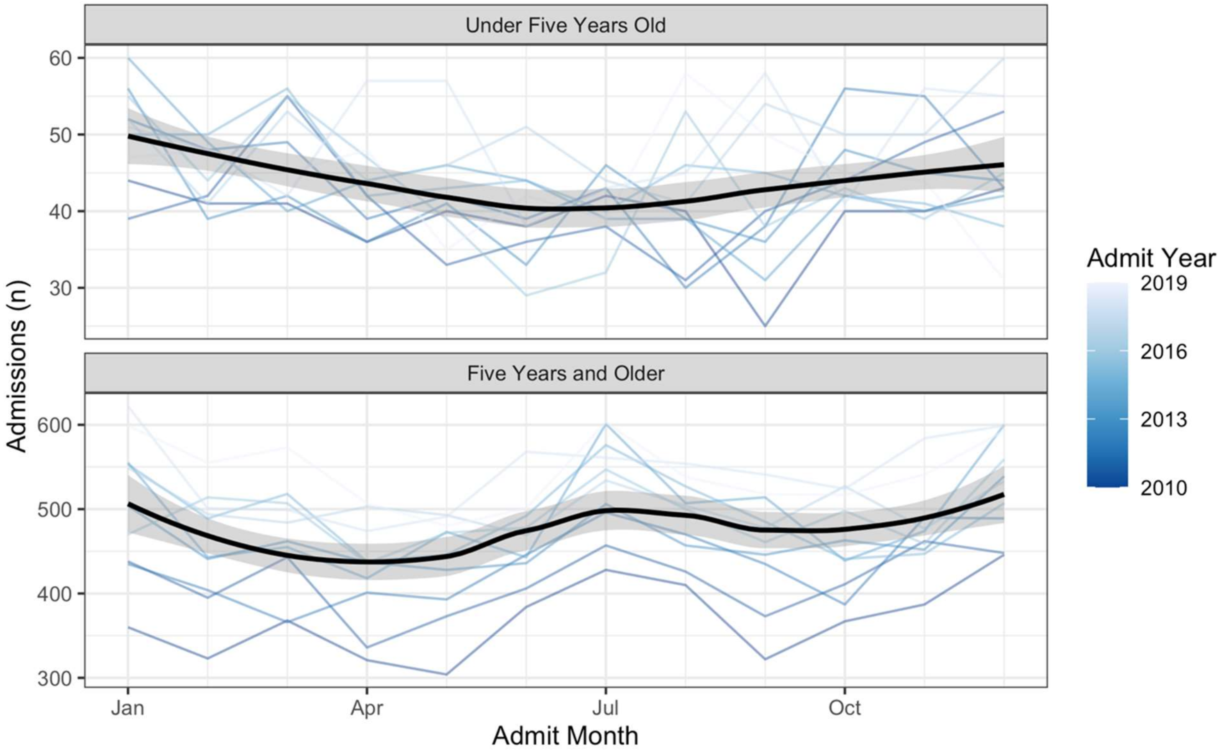

© 2021 Pelletier et al. *JAMA Network Open.*

eFigure 6. Seasonality of Hypoplastic Left Heart Syndrome by Age. Each panel shows the number of monthly admissions for hypoplastic left heart syndrome between 1/2010 and 12/2019. Panels are faceted according to the age at admission, specified in years.

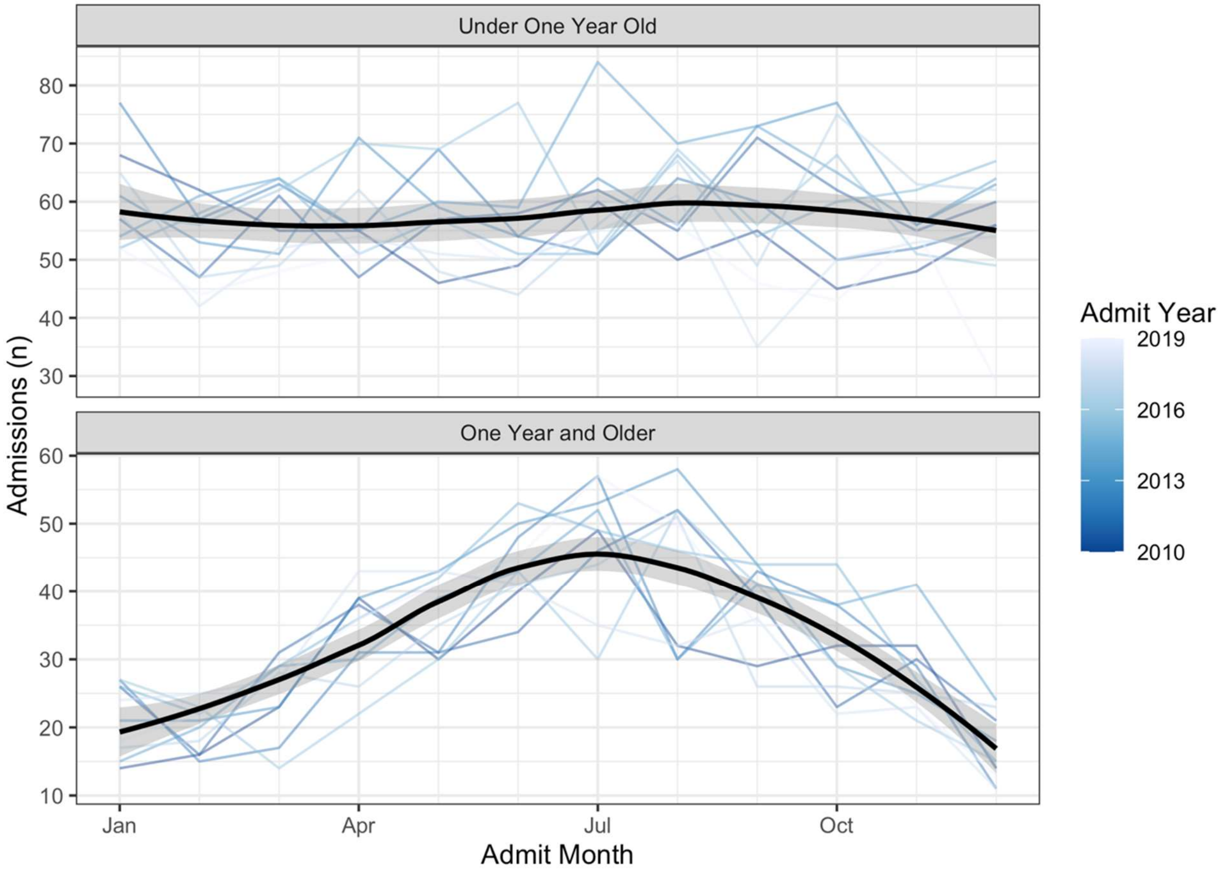

© 2021 Pelletier et al. *JAMA Network Open.*

eFigure 7. Ensemble Forecasts for Additional Diagnoses. Each panel shows the predicted number of monthly admissions for a specified diagnosis between 1/2010 and 6/2020, according to ensemble forecasting models including Autoregressive Integrated Moving Average (ARIMA), neural network, and LOcally Estimated Scatterplot Smoothing (LOESS) algorithms weighted on time series cross-validation. All models were trained on data from 1/2010 through 6/2019, and the testing window for each model is shown. The blue line is the model estimate, and the dark and light blue shaded regions are the model 80% and 95% confidence intervals, respectively. The red line is the actual number of admissions for each month. The vertical line represents January 2020. The numbers in the top left and top right of each pane represent the model mean absolute percentage error (MAPE) for 7/2019-12/2019 and 1/2020-6/2020, respectively. Panel A shows additional general diagnoses, and Panel B shows cardiac diagnoses. ASD: atrial septal defect, COA: coarctation of the aorta, HLHS: hypoplastic left heart syndrome, TOF: Tetralogy of Fallot.

© 2021 Pelletier et al. *JAMA Network Open.*

A

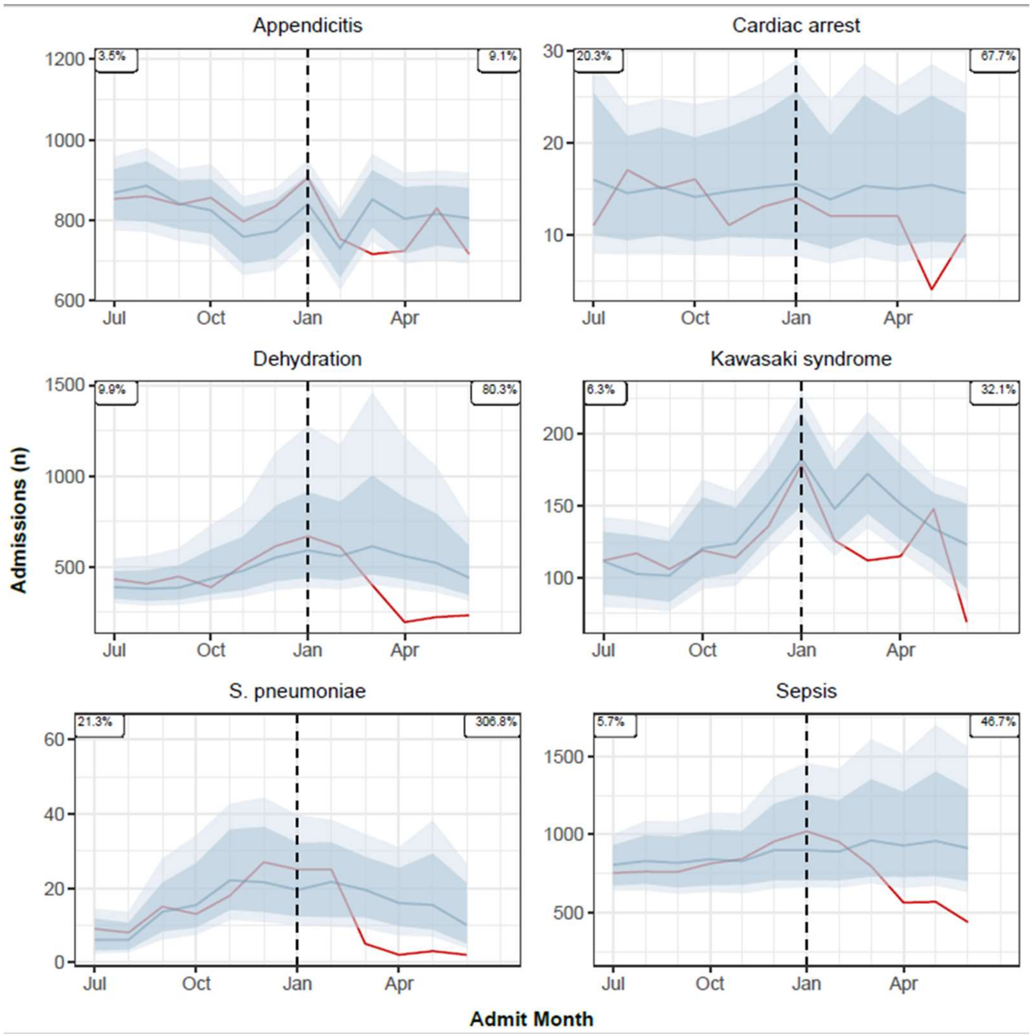

© 2021 Pelletier et al. *JAMA Network Open.*

B

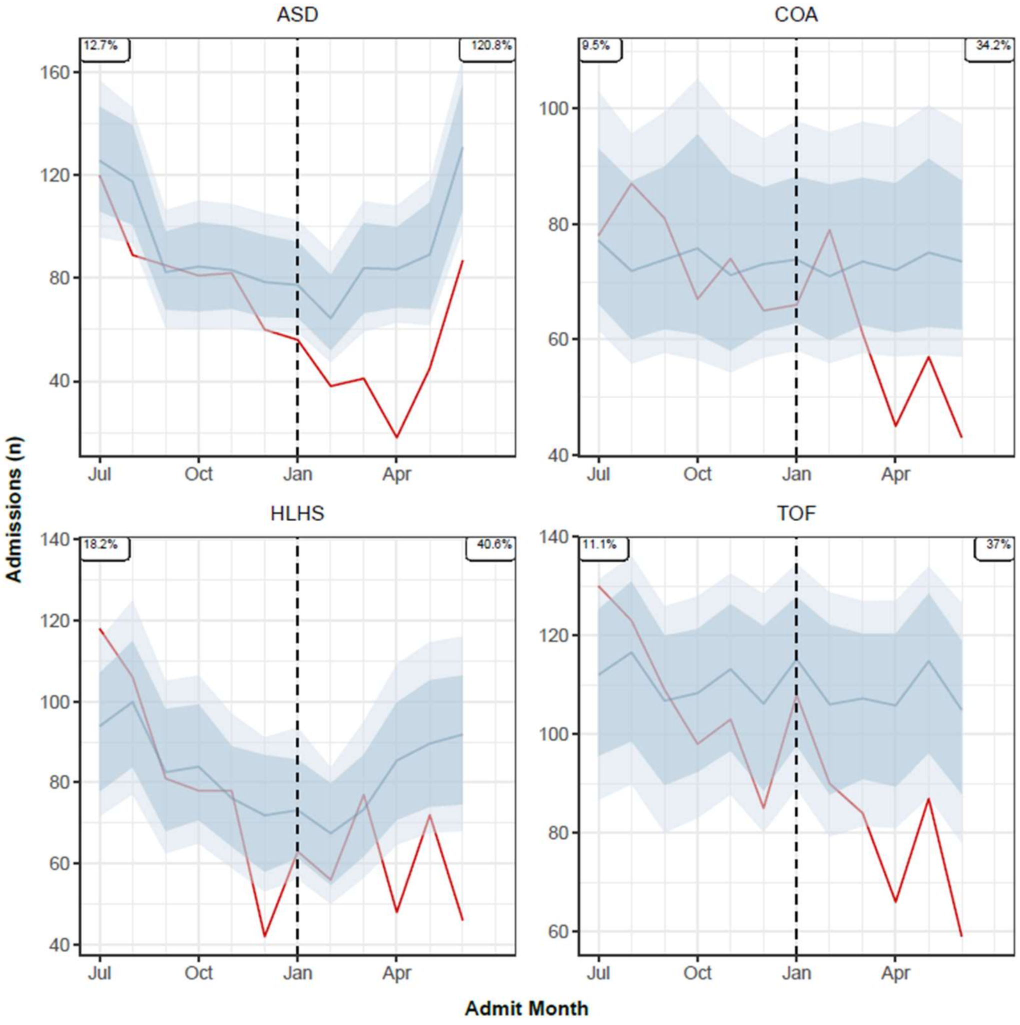

© 2021 Pelletier et al. *JAMA Network Open.*

eFigure 8. GDP-Adjusted Quarterly Total Hospital Charges. Each bar represents the total hospital charges for a given quarter and year in billions of dollars. All charges are adjusted for the Centers for Medicare & Medicaid Services wage/price index according to hospital zip code, and the quarterly Gross Domestic Product provided by the Bureau of Economic Analysis, and expressed in quarter one, 2010 dollars.

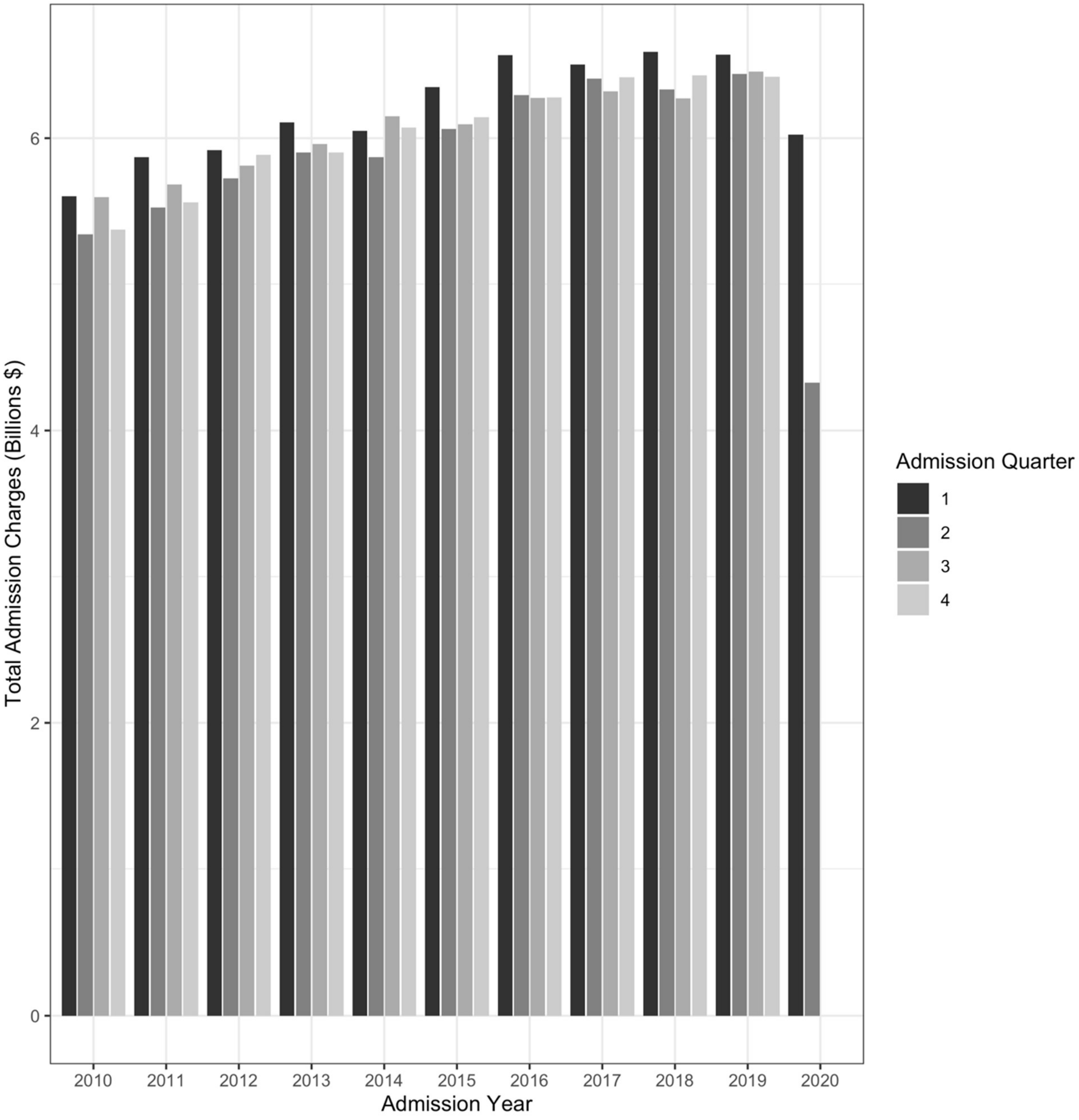

© 2021 Pelletier et al. *JAMA Network Open.*

eFigure 9. GDP-Adjusted Quarterly Per-Admission Hospital Charges. Each box-and-whiskers plot represents the cost per hospital admission for a given quarter and year in thousands of dollars. The center line represents the median, and the hinges represent the 25th and 75th percentiles. The whiskers extend 1.5 times the interquartile range below and above the 25th and 75th percentiles, respectively. The notch represents the confidence interval of the median and is calculated as 1.58 times the interquartile range divided by the square root of the sample size.

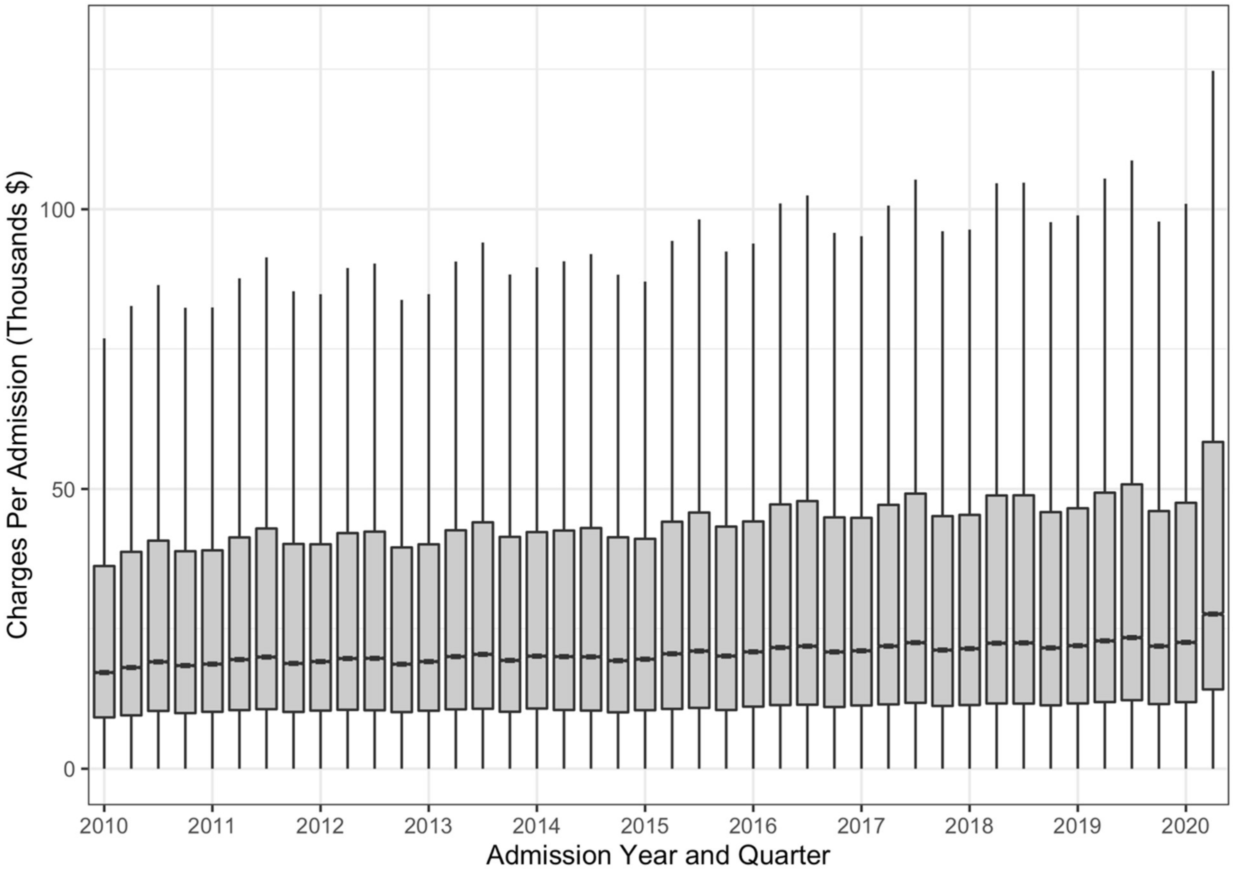

© 2021 Pelletier et al. *JAMA Network Open.*

eFigure 10. Seasonality of Exploratory Analyses. The top two panes show all diagnoses coded as “acute respiratory failure” and the bottom two panes show all hospital encounters during which the patient died. The upper left and lower left panes are identical in formatting to Figure 2. Each panel shows the number of monthly admissions for a specified diagnosis between 1/2010 and 6/2020. Each blue line represents an admission year between 2010 and 2019, with progressively lighter shades of blue representing later years. The black line represents the line of best fit for the data using LOcally Estimated Scatterplot Smoothing (LOESS). The gray bar represents the 95% confidence interval of the regression line. The red line shows the number of monthly admissions from 1/2020 through 6/2020. The upper right and lower right panes are identical in formatting to Figure 3. Each panel shows the predicted number of monthly admissions for a specified diagnosis between 1/2010 and 6/2020, according to ensemble forecasting models including Autoregressive Integrated Moving Average (ARIMA), neural network, and LOcally Estimated Scatterplot Smoothing (LOESS) algorithms weighted on time series cross-validation. All models were trained on data from 1/2010 through 6/2019, and the testing window for each model is shown. The blue line is the model estimate, and the dark and light blue shaded regions are the model 80% and 95% confidence intervals, respectively. The red line is the actual number of admissions for each month. The vertical line represents January 2020. The numbers in the top left and top right of each pane represent the model mean absolute percentage error (MAPE) for 7/2019-12/2019 and 1/2020-6/2020, respectively.

© 2021 Pelletier et al. *JAMA Network Open.*

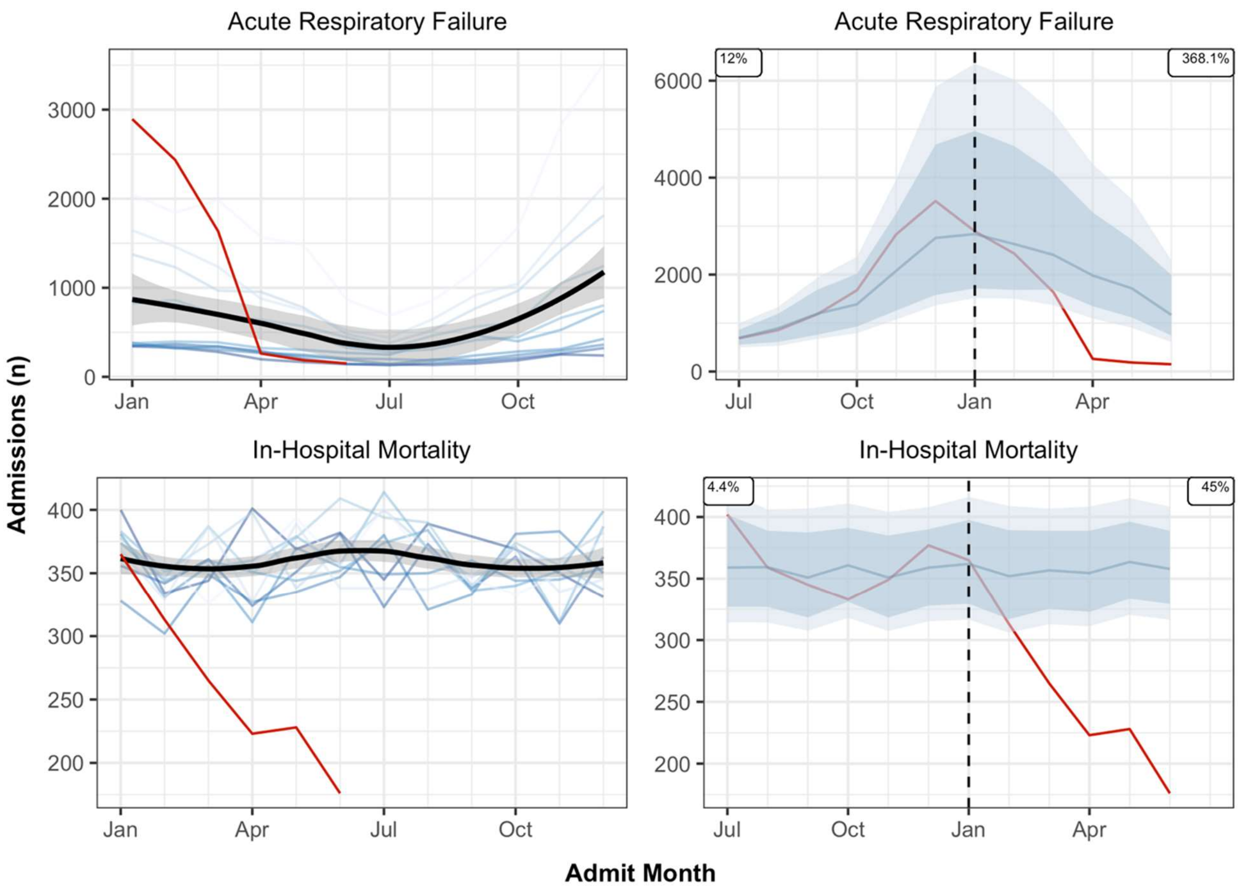

© 2021 Pelletier et al. *JAMA Network Open.*

eTable 1. List of Included Diagnostic Codes

Diagnosis	ICD Version	ICD Code	Number of Encounters
Appendicitis	10	K352	12674
Appendicitis	10	K35890	193
Appendicitis	10	K3530	1082
Appendicitis	10	K353	9281
Appendicitis	10	K3580	10832
Appendicitis	10	K3532	5632
Appendicitis	10	K3533	3929
Appendicitis	10	K3531	370
Appendicitis	10	K35891	526
Appendicitis	10	K3589	613
Appendicitis	10	K3520	1131
Appendicitis	10	K3521	815
Appendicitis	9	5400	22313
Appendicitis	9	5409	32764
Appendicitis	9	5401	11239
ASD	10	Q211	4885
ASD	9	7455	5902
Asthma	10	J4542	15030
Asthma	10	J4532	9491

© 2021 Pelletier et al. *JAMA Network Open.*

Diagnosis	ICD Version	ICD Code	Number of Encounters
Asthma	10	J4522	9875
Asthma	10	J4521	4817
Asthma	10	J45901	7574
Asthma	10	J4552	6302
Asthma	10	J4531	4124
Asthma	10	J4530	86
Asthma	10	J4541	4880
Asthma	10	J45909	952
Asthma	10	J45902	9166
Asthma	10	J4551	1570
Asthma	10	J45990	15
Asthma	10	J4550	57
Asthma	10	J4520	141
Asthma	10	J45991	35
Asthma	10	J4540	94
Asthma	10	J45998	8
Asthma	9	49301	21874
Asthma	9	49302	13499
Asthma	9	49300	945
Asthma	9	49311	654

© 2021 Pelletier et al. *JAMA Network Open.*

Diagnosis	ICD Version	ICD Code	Number of Encounters
Asthma	9	49391	50012
Asthma	9	49381	31
Asthma	9	49392	38107
Asthma	9	49321	49
Asthma	9	49382	94
Asthma	9	49322	94
Asthma	9	49312	1105
Asthma	9	49390	2740
Asthma	9	49310	39
Asthma	9	49320	26
Birth	10	Z381	366
Birth	10	Z3831	5872
Birth	10	Z3801	41063
Birth	10	Z3800	71558
Birth	10	Z3830	1640
Birth	10	Z3862	359
Birth	10	Z3869	3
Birth	10	Z3864	35
Birth	10	Z3861	25
Birth	10	Z382	6

© 2021 Pelletier et al. *JAMA Network Open.*

Diagnosis	ICD Version	ICD Code	Number of Encounters
Birth	10	Z384	12
Birth	10	Z3866	9
Birth	9	V301	509
Birth	9	7689	121
Birth	9	7684	8
Birth	9	V3000	63896
Birth	9	V3001	35099
Birth	9	V3100	1740
Birth	9	V3101	5991
Birth	9	V3401	678
Birth	9	7682	3
Birth	9	7683	14
Birth	9	V311	13
Birth	9	V270	4
Birth	9	V3400	21
Birth	9	V391	1
Birth	9	V3900	10
Birth	9	V3901	1
Birth	9	V331	2
Bronchiolitis	10	J210	46823

© 2021 Pelletier et al. *JAMA Network Open.*

Diagnosis	ICD Version	ICD Code	Number of Encounters
Bronchiolitis	10	J218	26817
Bronchiolitis	10	J211	3150
Bronchiolitis	10	J219	29601
Bronchiolitis	9	46611	66605
Bronchiolitis	9	46619	55641
Cardiac arrest	10	I469	777
Cardiac arrest	10	I468	43
Cardiac arrest	10	I462	11
Cardiac arrest	9	4275	726
COA	10	Q251	4253
COA	9	74710	5646
Dehydration	10	E860	28279
Dehydration	10	E861	74
Dehydration	10	E869	29
Dehydration	9	27651	34723
Dehydration	9	27652	130
Dehydration	9	27650	56
Diabetic ketoacidosis	10	E1010	31903
Diabetic ketoacidosis	10	E1011	266
Diabetic ketoacidosis	9	25013	31754

© 2021 Pelletier et al. *JAMA Network Open.*

Diagnosis	ICD Version	ICD Code	Number of Encounters
Diabetic ketoacidosis	9	25012	852
Diabetic ketoacidosis	9	25011	824
Diabetic ketoacidosis	9	25010	89
HLHS	10	Q234	4822
HLHS	9	7467	6261
Kawasaki syndrome	10	M303	7649
Kawasaki syndrome	9	4461	9004
Mental health	10	F329	18030
Mental health	10	F3481	5405
Mental health	10	F323	1614
Mental health	10	F333	2060
Mental health	10	F331	1912
Mental health	10	F332	9558
Mental health	10	F4310	2804
Mental health	10	F411	1332
Mental health	10	F6381	3530
Mental health	10	F840	1688
Mental health	10	F322	5914
Mental health	10	F29	1249
Mental health	10	F913	1350

Diagnosis	ICD Version	ICD Code	Number of Encounters
Mental health	10	F339	1486
Mental health	10	F3189	117
Mental health	10	F4323	381
Mental health	10	F259	106
Mental health	10	F23	576
Mental health	10	F445	1194
Mental health	10	F419	1039
Mental health	10	F919	2423
Mental health	10	F12980	2
Mental health	10	F941	354
Mental health	10	F39	3577
Mental health	10	F5000	1035
Mental health	10	F13950	1
Mental health	10	F10129	477
Mental health	10	F321	1352
Mental health	10	F911	1297
Mental health	10	F4321	580
Mental health	10	F319	1632
Mental health	10	F202	173
Mental health	10	F410	224

© 2021 Pelletier et al. *JAMA Network Open.*

Diagnosis	ICD Version	ICD Code	Number of Encounters
Mental health	10	F489	14
Mental health	10	F10121	31
Mental health	10	F909	949
Mental health	10	F450	32
Mental health	10	F4489	108
Mental health	10	F3181	305
Mental health	10	F444	526
Mental health	10	F209	399
Mental health	10	F959	184
Mental health	10	F449	668
Mental health	10	F4320	315
Mental health	10	F918	654
Mental health	10	F902	1091
Mental health	10	F459	97
Mental health	10	F19129	21
Mental health	10	F5001	4223
Mental health	10	F12188	113
Mental health	10	F12950	7
Mental health	10	F4541	83
Mental health	10	F3132	73

© 2021 Pelletier et al. *JAMA Network Open.*

Diagnosis	ICD Version	ICD Code	Number of Encounters
Mental health	10	F3130	116
Mental health	10	F4325	1184
Mental health	10	F349	35
Mental health	10	F328	149
Mental health	10	F0631	7
Mental health	10	F639	1524
Mental health	10	F1994	80
Mental health	10	F19959	53
Mental health	10	F5089	471
Mental health	10	F958	65
Mental health	10	F28	74
Mental health	10	F458	221
Mental health	10	F061	104
Mental health	10	F19159	15
Mental health	10	F429	280
Mental health	10	F82	21
Mental health	10	F320	63
Mental health	10	F312	324
Mental health	10	F3289	762
Mental health	10	F3160	191

© 2021 Pelletier et al. *JAMA Network Open.*

Diagnosis	ICD Version	ICD Code	Number of Encounters
Mental health	10	F15951	5
Mental health	10	F302	47
Mental health	10	F12988	108
Mental health	10	F4329	53
Mental health	10	F802	16
Mental health	10	F940	14
Mental health	10	F5002	801
Mental health	10	F430	118
Mental health	10	F15129	20
Mental health	10	F952	93
Mental health	10	F951	14
Mental health	10	F12959	24
Mental health	10	F12951	9
Mental health	10	F509	852
Mental health	10	F251	112
Mental health	10	F2081	86
Mental health	10	F418	251
Mental health	10	F200	58
Mental health	10	F22	85
Mental health	10	F42	82

© 2021 Pelletier et al. *JAMA Network Open.*

Diagnosis	ICD Version	ICD Code	Number of Encounters
Mental health	10	F4312	346
Mental health	10	F12288	22
Mental health	10	F250	119
Mental health	10	F10120	29
Mental health	10	F4324	128
Mental health	10	F984	199
Mental health	10	F842	121
Mental health	10	F515	3
Mental health	10	F989	32
Mental health	10	F12159	24
Mental health	10	F05	33
Mental health	10	F19939	28
Mental health	10	F513	13
Mental health	10	F9821	28
Mental health	10	F819	3
Mental health	10	F14129	5
Mental health	10	F12129	16
Mental health	10	F502	243
Mental health	10	F988	40
Mental health	10	F88	68

© 2021 Pelletier et al. *JAMA Network Open.*

Diagnosis	ICD Version	ICD Code	Number of Encounters
Mental health	10	F950	25
Mental health	10	F324	32
Mental health	10	F5082	759
Mental health	10	F4542	26
Mental health	10	F309	40
Mental health	10	F801	22
Mental health	10	F348	864
Mental health	10	F408	7
Mental health	10	F2089	60
Mental health	10	F15159	5
Mental health	10	F900	50
Mental health	10	F3113	43
Mental health	10	F901	88
Mental health	10	F16129	7
Mental health	10	F809	58
Mental health	10	F8089	20
Mental health	10	F3163	67
Mental health	10	F446	70
Mental health	10	F447	302
Mental health	10	F1523	9

© 2021 Pelletier et al. *JAMA Network Open.*

Diagnosis	ICD Version	ICD Code	Number of Encounters
Mental health	10	F641	3
Mental health	10	F0781	345
Mental health	10	F315	129
Mental health	10	F340	39
Mental health	10	F451	34
Mental health	10	F13239	48
Mental health	10	F10229	9
Mental health	10	F341	251
Mental health	10	F12122	1
Mental health	10	F1914	47
Mental health	10	F508	173
Mental health	10	F1123	126
Mental health	10	F310	24
Mental health	10	F21	4
Mental health	10	F1010	9
Mental health	10	F12929	40
Mental health	10	F19921	8
Mental health	10	F1510	14
Mental health	10	F19951	18
Mental health	10	F068	28

© 2021 Pelletier et al. *JAMA Network Open.*

Diagnosis	ICD Version	ICD Code	Number of Encounters
Mental health	10	F4010	73
Mental health	10	F428	28
Mental health	10	F4322	97
Mental health	10	F3162	72
Mental health	10	F70	13
Mental health	10	F9829	87
Mental health	10	F201	13
Mental health	10	F3164	82
Mental health	10	F609	11
Mental health	10	F438	43
Mental health	10	F1610	5
Mental health	10	F14150	1
Mental health	10	F8081	22
Mental health	10	F1290	15
Mental health	10	F19239	14
Mental health	10	F640	56
Mental health	10	F19151	5
Mental health	10	F12251	7
Mental health	10	F12151	16
Mental health	10	F19250	1

© 2021 Pelletier et al. *JAMA Network Open.*

Diagnosis	ICD Version	ICD Code	Number of Encounters
Mental health	10	F308	16
Mental health	10	F1990	7
Mental health	10	F4311	224
Mental health	10	F4001	10
Mental health	10	F845	33
Mental health	10	F603	43
Mental health	10	F3341	56
Mental health	10	F4522	5
Mental health	10	F1223	2
Mental health	10	F12150	11
Mental health	10	F24	2
Mental health	10	F19259	3
Mental health	10	F19929	4
Mental health	10	F1524	3
Mental health	10	F79	13
Mental health	10	F15950	1
Mental health	10	F16151	4
Mental health	10	F3489	86
Mental health	10	F12921	8
Mental health	10	F19150	3

© 2021 Pelletier et al. *JAMA Network Open.*

Diagnosis	ICD Version	ICD Code	Number of Encounters
Mental health	10	F481	5
Mental health	10	F930	53
Mental health	10	F424	3
Mental health	10	F19231	1
Mental health	10	F10929	21
Mental health	10	F15251	1
Mental health	10	F71	11
Mental health	10	F3010	6
Mental health	10	F16150	2
Mental health	10	F16188	2
Mental health	10	F912	34
Mental health	10	F203	15
Mental health	10	F59	3
Mental health	10	F439	85
Mental health	10	F849	15
Mental health	10	F0630	16
Mental health	10	F89	9
Mental health	10	F4000	4
Mental health	10	F15151	2
Mental health	10	F314	106

© 2021 Pelletier et al. *JAMA Network Open.*

Diagnosis	ICD Version	ICD Code	Number of Encounters
Mental health	10	F15150	1
Mental health	10	F6389	43
Mental health	10	F519	4
Mental health	10	F514	47
Mental health	10	F16950	1
Mental health	10	F16959	3
Mental health	10	F16159	3
Mental health	10	F1594	3
Mental health	10	F18159	1
Mental health	10	F10959	2
Mental health	10	F258	3
Mental health	10	F981	15
Mental health	10	F330	70
Mental health	10	F3110	68
Mental health	10	F73	3
Mental health	10	F12220	1
Mental health	10	F070	4
Mental health	10	F518	19
Mental health	10	F6810	12
Mental health	10	F14929	2

Diagnosis	ICD Version	ICD Code	Number of Encounters
Mental health	10	F12920	6
Mental health	10	F19120	3
Mental health	10	F19932	1
Mental health	10	F15929	3
Mental health	10	F1499	1
Mental health	10	F99	19
Mental health	10	F983	8
Mental health	10	F1220	7
Mental health	10	F1910	27
Mental health	10	F4011	5
Mental health	10	F14288	1
Mental health	10	F3177	9
Mental health	10	F338	64
Mental health	10	F1919	3
Mental health	10	F1920	4
Mental health	10	F908	21
Mental health	10	F53	10
Mental health	10	F8082	2
Mental health	10	F642	18
Mental health	10	F3112	48

© 2021 Pelletier et al. *JAMA Network Open.*

Diagnosis	ICD Version	ICD Code	Number of Encounters
Mental health	10	F11921	1
Mental health	10	F1219	1
Mental health	10	F16988	1
Mental health	10	F15959	5
Mental health	10	F12259	13
Mental health	10	F1210	20
Mental health	10	F10231	1
Mental health	10	F09	27
Mental health	10	F6089	31
Mental health	10	F1094	1
Mental health	10	F0634	3
Mental health	10	F13230	4
Mental health	10	F11151	1
Mental health	10	F1124	1
Mental health	10	F13231	6
Mental health	10	F488	23
Mental health	10	F68A	9
Mental health	10	F1319	1
Mental health	10	F1110	5
Mental health	10	F649	8

© 2021 Pelletier et al. *JAMA Network Open.*

Diagnosis	ICD Version	ICD Code	Number of Encounters
Mental health	10	F6813	4
Mental health	10	F1099	4
Mental health	10	F633	4
Mental health	10	F064	5
Mental health	10	F6812	10
Mental health	10	F1593	5
Mental health	10	F11929	2
Mental health	10	F16951	5
Mental health	10	F1120	7
Mental health	10	F1999	3
Mental health	10	F1924	7
Mental health	10	F325	6
Mental health	10	F1694	1
Mental health	10	F1590	4
Mental health	10	F12120	3
Mental health	10	F1514	6
Mental health	10	F1014	8
Mental health	10	F12229	4
Mental health	10	F16921	1
Mental health	10	F16122	2

© 2021 Pelletier et al. *JAMA Network Open.*

Diagnosis	ICD Version	ICD Code	Number of Encounters
Mental health	10	F1329	1
Mental health	10	F1024	2
Mental health	10	F15259	2
Mental health	10	F16121	4
Mental health	10	F3170	3
Mental health	10	F19950	6
Mental health	10	F843	3
Mental health	10	F4481	6
Mental health	10	F15229	4
Mental health	10	F18988	2
Mental health	10	F13232	2
Mental health	10	F3281	7
Mental health	10	F13129	6
Mental health	10	F1320	4
Mental health	10	F0390	1
Mental health	10	F1894	1
Mental health	10	F1020	3
Mental health	10	F12280	2
Mental health	10	F13929	4
Mental health	10	F5105	2

© 2021 Pelletier et al. *JAMA Network Open.*

Diagnosis	ICD Version	ICD Code	Number of Encounters
Mental health	10	F1410	4
Mental health	10	F19980	2
Mental health	10	F3340	9
Mental health	10	F3131	5
Mental health	10	F3175	10
Mental health	10	F062	4
Mental health	10	F3111	7
Mental health	10	F422	31
Mental health	10	F10180	1
Mental health	10	F5081	6
Mental health	10	F13280	1
Mental health	10	F3342	3
Mental health	10	F12221	1
Mental health	10	F1690	2
Mental health	10	F19221	1
Mental health	10	F11129	4
Mental health	10	F19251	1
Mental health	10	F15982	1
Mental health	10	F205	2
Mental health	10	F19931	1

© 2021 Pelletier et al. *JAMA Network Open.*

Diagnosis	ICD Version	ICD Code	Number of Encounters
Mental health	10	F1293	2
Mental health	10	F409	3
Mental health	10	F3174	1
Mental health	10	F6811	1
Mental health	10	F060	13
Mental health	10	F12250	1
Mental health	10	F13121	2
Mental health	10	F13951	1
Mental health	10	F19188	2
Mental health	10	F19988	2
Mental health	10	F15921	1
Mental health	10	F14121	2
Mental health	10	F0789	4
Mental health	10	F15188	2
Mental health	10	F1614	2
Mental health	10	F14922	1
Mental health	10	F10221	1
Mental health	10	F1114	2
Mental health	10	F1194	2
Mental health	10	F6081	1

© 2021 Pelletier et al. *JAMA Network Open.*

Diagnosis	ICD Version	ICD Code	Number of Encounters
Mental health	10	F650	1
Mental health	10	F1699	1
Mental health	10	F16980	2
Mental health	10	F3173	8
Mental health	10	F631	6
Mental health	10	F948	2
Mental health	10	F3161	7
Mental health	10	F0151	3
Mental health	10	F0633	1
Mental health	10	F942	6
Mental health	10	F413	2
Mental health	10	F1299	2
Mental health	10	F1310	8
Mental health	10	F605	4
Mental health	10	F1190	1
Mental health	10	F602	2
Mental health	10	F5119	1
Mental health	10	F19230	3
Mental health	10	F938	5
Mental health	10	F15122	2

© 2021 Pelletier et al. *JAMA Network Open.*

Diagnosis	ICD Version	ICD Code	Number of Encounters
Mental health	10	F1494	1
Mental health	10	F54	1
Mental health	10	F441	2
Mental health	10	F440	11
Mental health	10	F11188	1
Mental health	10	F12121	4
Mental health	10	F40298	1
Mental health	10	F16929	2
Mental health	10	F72	2
Mental health	10	F10239	5
Mental health	10	F11229	1
Mental health	10	F0150	1
Mental health	10	F1399	1
Mental health	10	F1390	1
Mental health	10	F939	7
Mental health	10	F19229	1
Mental health	10	F19180	1
Mental health	10	F688	1
Mental health	10	F12922	2
Mental health	10	F910	2

© 2021 Pelletier et al. *JAMA Network Open.*

Diagnosis	ICD Version	ICD Code	Number of Encounters
Mental health	10	F442	1
Mental health	10	F528	2
Mental health	10	F78	1
Mental health	10	F13229	2
Mental health	10	F1193	1
Mental health	10	F529	1
Mental health	10	F1011	1
Mental health	10	F810	1
Mental health	10	F12180	1
Mental health	10	F5101	1
Mental health	10	F11288	1
Mental health	10	F13988	1
Mental health	10	F848	4
Mental health	10	F1419	1
Mental health	10	F19280	1
Mental health	10	F19121	2
Mental health	10	F14151	1
Mental health	10	F16920	1
Mental health	10	F04	1
Mental health	10	F11150	1

© 2021 Pelletier et al. *JAMA Network Open.*

Diagnosis	ICD Version	ICD Code	Number of Encounters
Mental health	10	F652	1
Mental health	10	F949	1
Mental health	10	F1424	1
Mental health	10	F13959	2
Mental health	10	F632	1
Mental health	10	F601	1
Mental health	10	F13151	1
Mental health	10	F13939	1
Mental health	10	F0632	1
Mental health	10	F15922	1
Mental health	9	29690	15191
Mental health	9	3064	119
Mental health	9	30746	144
Mental health	9	31400	18
Mental health	9	29620	5343
Mental health	9	2989	2679
Mental health	9	311	8633
Mental health	9	29624	1306
Mental health	9	29530	92
Mental health	9	3013	452

© 2021 Pelletier et al. *JAMA Network Open.*

Diagnosis	ICD Version	ICD Code	Number of Encounters
Mental health	9	30981	2220
Mental health	9	31381	1201
Mental health	9	30000	1113
Mental health	9	3129	2465
Mental health	9	29625	18
Mental health	9	3003	343
Mental health	9	31401	1807
Mental health	9	31234	3168
Mental health	9	29633	4532
Mental health	9	30011	2206
Mental health	9	29634	1329
Mental health	9	3102	750
Mental health	9	29622	673
Mental health	9	29600	30
Mental health	9	29623	4329
Mental health	9	29284	126
Mental health	9	3090	373
Mental health	9	29635	20
Mental health	9	29900	1030
Mental health	9	29632	557

© 2021 Pelletier et al. *JAMA Network Open.*

Diagnosis	ICD Version	ICD Code	Number of Encounters
Mental health	9	30747	121
Mental health	9	30750	1353
Mental health	9	29570	440
Mental health	9	3004	556
Mental health	9	3009	280
Mental health	9	29680	2447
Mental health	9	3071	4259
Mental health	9	29532	18
Mental health	9	2920	321
Mental health	9	30500	678
Mental health	9	30082	145
Mental health	9	29699	948
Mental health	9	3073	209
Mental health	9	3099	190
Mental health	9	30781	156
Mental health	9	30002	526
Mental health	9	30759	529
Mental health	9	2967	1252
Mental health	9	3079	256
Mental health	9	29650	405

© 2021 Pelletier et al. *JAMA Network Open.*

Diagnosis	ICD Version	ICD Code	Number of Encounters
Mental health	9	30720	335
Mental health	9	29990	197
Mental health	9	31389	424
Mental health	9	30722	96
Mental health	9	30751	344
Mental health	9	31200	299
Mental health	9	31230	1418
Mental health	9	316	40
Mental health	9	30009	57
Mental health	9	31531	64
Mental health	9	3139	104
Mental health	9	29540	85
Mental health	9	30001	206
Mental health	9	29689	431
Mental health	9	3159	47
Mental health	9	30721	26
Mental health	9	2929	62
Mental health	9	31289	179
Mental health	9	30753	221
Mental health	9	30928	190

© 2021 Pelletier et al. *JAMA Network Open.*

Diagnosis	ICD Version	ICD Code	Number of Encounters
Mental health	9	29212	119
Mental health	9	3077	31
Mental health	9	30754	30
Mental health	9	3158	81
Mental health	9	29630	1837
Mental health	9	30501	21
Mental health	9	30570	22
Mental health	9	3083	35
Mental health	9	30723	107
Mental health	9	31539	66
Mental health	9	29644	289
Mental health	9	31239	29
Mental health	9	29590	237
Mental health	9	30921	86
Mental health	9	30520	92
Mental health	9	3094	409
Mental health	9	29660	503
Mental health	9	29980	184
Mental health	9	2981	2
Mental health	9	29211	19

© 2021 Pelletier et al. *JAMA Network Open.*

Diagnosis	ICD Version	ICD Code	Number of Encounters
Mental health	9	29664	152
Mental health	9	30300	33
Mental health	9	2973	4
Mental health	9	29289	49
Mental health	9	31210	19
Mental health	9	3069	28
Mental health	9	3093	69
Mental health	9	30590	67
Mental health	9	30481	1
Mental health	9	31282	43
Mental health	9	30430	7
Mental health	9	29281	83
Mental health	9	3076	2
Mental health	9	29640	324
Mental health	9	29189	7
Mental health	9	29580	18
Mental health	9	3060	39
Mental health	9	30924	46
Mental health	9	30029	13
Mental health	9	3026	3

© 2021 Pelletier et al. *JAMA Network Open.*

Diagnosis	ICD Version	ICD Code	Number of Encounters
Mental health	9	30081	44
Mental health	9	3080	13
Mental health	9	3061	44
Mental health	9	3006	3
Mental health	9	29654	133
Mental health	9	30745	4
Mental health	9	29383	39
Mental health	9	30502	23
Mental health	9	2930	10
Mental health	9	319	13
Mental health	9	30592	5
Mental health	9	29621	49
Mental health	9	30740	3
Mental health	9	30021	28
Mental health	9	29663	119
Mental health	9	31281	104
Mental health	9	29652	18
Mental health	9	29510	21
Mental health	9	31532	15
Mental health	9	29631	22

© 2021 Pelletier et al. *JAMA Network Open.*

Diagnosis	ICD Version	ICD Code	Number of Encounters
Mental health	9	3155	8
Mental health	9	29653	117
Mental health	9	3068	28
Mental health	9	29910	6
Mental health	9	3014	2
Mental health	9	30789	6
Mental health	9	29574	6
Mental health	9	3089	52
Mental health	9	30302	2
Mental health	9	29661	3
Mental health	9	29389	8
Mental health	9	30301	10
Mental health	9	29662	18
Mental health	9	2980	28
Mental health	9	29572	8
Mental health	9	29645	6
Mental health	9	29573	2
Mental health	9	29643	62
Mental health	9	29603	4
Mental health	9	31203	12

© 2021 Pelletier et al. *JAMA Network Open.*

Diagnosis	ICD Version	ICD Code	Number of Encounters
Mental health	9	30019	13
Mental health	9	30013	1
Mental health	9	30659	3
Mental health	9	30480	13
Mental health	9	3109	4
Mental health	9	30752	5
Mental health	9	29420	1
Mental health	9	30521	6
Mental health	9	30089	36
Mental health	9	30742	3
Mental health	9	29520	25
Mental health	9	2949	17
Mental health	9	30023	19
Mental health	9	29181	10
Mental health	9	3062	3
Mental health	9	30749	1
Mental health	9	30390	3
Mental health	9	31535	17
Mental health	9	3070	4
Mental health	9	29562	9

© 2021 Pelletier et al. *JAMA Network Open.*

Diagnosis	ICD Version	ICD Code	Number of Encounters
Mental health	9	3133	2
Mental health	9	2971	24
Mental health	9	3101	19
Mental health	9	29642	15
Mental health	9	2988	32
Mental health	9	30400	21
Mental health	9	3180	7
Mental health	9	30410	1
Mental health	9	30015	16
Mental health	9	29544	2
Mental health	9	2979	8
Mental health	9	29560	3
Mental health	9	29594	4
Mental health	9	30780	39
Mental health	9	30183	8
Mental health	9	30530	10
Mental health	9	2910	4
Mental health	9	30113	30
Mental health	9	30550	20
Mental health	9	30012	6

© 2021 Pelletier et al. *JAMA Network Open.*

Diagnosis	ICD Version	ICD Code	Number of Encounters
Mental health	9	30653	2
Mental health	9	30540	3
Mental health	9	30151	15
Mental health	9	3154	8
Mental health	9	30560	7
Mental health	9	29285	10
Mental health	9	30016	1
Mental health	9	30591	4
Mental health	9	29534	8
Mental health	9	29636	1
Mental health	9	29626	2
Mental health	9	30929	5
Mental health	9	30983	2
Mental health	9	30112	1
Mental health	9	29592	2
Mental health	9	29533	2
Mental health	9	29604	10
Mental health	9	31323	10
Mental health	9	30989	30
Mental health	9	30522	3

© 2021 Pelletier et al. *JAMA Network Open.*

Diagnosis	ICD Version	ICD Code	Number of Encounters
Mental health	9	30401	7
Mental health	9	2939	3
Mental health	9	3091	7
Mental health	9	29681	1
Mental health	9	3181	3
Mental health	9	317	3
Mental health	9	30744	2
Mental health	9	3124	2
Mental health	9	29682	4
Mental health	9	3029	1
Mental health	9	31233	3
Mental health	9	30189	5
Mental health	9	31232	1
Mental health	9	3152	6
Mental health	9	3108	1
Mental health	9	30470	8
Mental health	9	30552	1
Mental health	9	30431	5
Mental health	9	30471	3
Mental health	9	3082	8

© 2021 Pelletier et al. *JAMA Network Open.*

Diagnosis	ICD Version	ICD Code	Number of Encounters
Mental health	9	30020	1
Mental health	9	31089	1
Mental health	9	29382	3
Mental health	9	3066	1
Mental health	9	30982	2
Mental health	9	2978	1
Mental health	9	29283	1
Mental health	9	29605	1
Mental health	9	30743	1
Mental health	9	29514	2
Mental health	9	29665	3
Mental health	9	30460	1
Mental health	9	2915	1
Mental health	9	29651	1
Mental health	9	30561	1
Mental health	9	29595	1
Mental health	9	30411	1
Mental health	9	30285	6
Mental health	9	3019	2
Mental health	9	30111	1

© 2021 Pelletier et al. *JAMA Network Open.*

Diagnosis	ICD Version	ICD Code	Number of Encounters
Mental health	9	30532	1
Mental health	9	30541	1
Mental health	9	3022	1
Mental health	9	2948	4
Mental health	9	31081	1
Mental health	9	3007	1
Mental health	9	3081	1
Mental health	9	3084	2
Mental health	9	31500	3
Mental health	9	3149	3
Mental health	9	29593	1
Mental health	9	30159	1
Mental health	9	30748	1
Mental health	9	3100	2
Mental health	9	30182	1
Mental health	9	3067	2
Mental health	9	2913	1
Mental health	9	29901	5
Mental health	9	30270	1
Mental health	9	30289	1

© 2021 Pelletier et al. *JAMA Network Open.*

Diagnosis	ICD Version	ICD Code	Number of Encounters
Mental health	9	31201	1
Mental health	9	31223	2
Mental health	9	31213	1
Mental health	9	29610	1
Mental health	9	3017	3
Mental health	9	29602	1
Mental health	9	29564	2
Mental health	9	2914	1
Mental health	9	30531	1
Mental health	9	30420	1
Mental health	9	30181	1
Mental health	9	29655	1
Mental health	9	29656	1
Mental health	9	31322	1
S. pneumoniae	10	J13	1019
S. pneumoniae	9	481	1799
Sepsis	10	A419	21280
Sepsis	10	A4151	2369
Sepsis	10	A4159	786
Sepsis	10	A021	239

© 2021 Pelletier et al. *JAMA Network Open.*

Diagnosis	ICD Version	ICD Code	Number of Encounters
Sepsis	10	R6521	319
Sepsis	10	I2690	7
Sepsis	10	A400	688
Sepsis	10	A4189	3067
Sepsis	10	A4101	1096
Sepsis	10	A403	742
Sepsis	10	A4152	493
Sepsis	10	A4102	702
Sepsis	10	A4150	311
Sepsis	10	A4181	334
Sepsis	10	A408	295
Sepsis	10	A414	112
Sepsis	10	A411	284
Sepsis	10	A413	249
Sepsis	10	A401	555
Sepsis	10	P369	3933
Sepsis	10	P360	463
Sepsis	10	P368	436
Sepsis	10	P3630	29
Sepsis	10	P362	182

© 2021 Pelletier et al. *JAMA Network Open.*

Diagnosis	ICD Version	ICD Code	Number of Encounters
Sepsis	10	P364	597
Sepsis	10	A409	163
Sepsis	10	P3619	110
Sepsis	10	P3639	90
Sepsis	10	P3610	17
Sepsis	10	T8144XA	41
Sepsis	10	B377	141
Sepsis	10	A4153	71
Sepsis	10	A427	7
Sepsis	10	R6520	51
Sepsis	10	A412	66
Sepsis	10	A5486	4
Sepsis	10	A327	12
Sepsis	10	P365	8
Sepsis	10	A227	2
Sepsis	10	T8112XA	2
Sepsis	10	O0387	10
Sepsis	10	O0337	10
Sepsis	10	O85	27
Sepsis	10	O0882	1

© 2021 Pelletier et al. *JAMA Network Open.*

Diagnosis	ICD Version	ICD Code	Number of Encounters
Sepsis	10	I76	3
Sepsis	10	O8681	1
Sepsis	9	0389	10024
Sepsis	9	03843	437
Sepsis	9	03842	1441
Sepsis	9	03811	839
Sepsis	9	0382	550
Sepsis	9	03849	1139
Sepsis	9	0380	1390
Sepsis	9	03812	597
Sepsis	9	99592	57
Sepsis	9	03840	258
Sepsis	9	77181	6153
Sepsis	9	0031	174
Sepsis	9	0383	108
Sepsis	9	03819	299
Sepsis	9	78552	6
Sepsis	9	03841	130
Sepsis	9	99591	50
Sepsis	9	03844	53

© 2021 Pelletier et al. *JAMA Network Open.*

Diagnosis	ICD Version	ICD Code	Number of Encounters
Sepsis	9	0545	17
Sepsis	9	42292	6
Sepsis	9	03810	83
Sepsis	9	449	3
Sepsis	9	41512	8
Sepsis	9	99802	1
Sepsis	9	67024	16
Sepsis	9	67034	1
TOF	10	Q213	6145
TOF	9	7452	7404
Trauma	10	S42452A	571
Trauma	10	S060X0A	1215
Trauma	10	S064X1A	187
Trauma	10	S02119A	804
Trauma	10	S02602B	32
Trauma	10	S89121A	115
Trauma	10	S82291P	2
Trauma	10	S52202B	87
Trauma	10	S064X9A	521
Trauma	10	S161XXA	51

© 2021 Pelletier et al. *JAMA Network Open.*

Diagnosis	ICD Version	ICD Code	Number of Encounters
Trauma	10	S79012A	295
Trauma	10	S52602B	29
Trauma	10	T1491XA	166
Trauma	10	T188XXA	132
Trauma	10	S91342A	41
Trauma	10	S82252A	146
Trauma	10	S134XXA	67
Trauma	10	S82152A	516
Trauma	10	S065X9A	1449
Trauma	10	S065X1S	1
Trauma	10	S32442A	15
Trauma	10	S023XXA	110
Trauma	10	S52501B	125
Trauma	10	S72451A	58
Trauma	10	S82261A	4
Trauma	10	S0231XA	169
Trauma	10	S83271A	6
Trauma	10	S83512A	130
Trauma	10	S52592A	45
Trauma	10	T18128A	268

© 2021 Pelletier et al. *JAMA Network Open.*

Diagnosis	ICD Version	ICD Code	Number of Encounters
Trauma	10	S59212A	2
Trauma	10	S59222A	67
Trauma	10	S62636B	12
Trauma	10	S02609B	10
Trauma	10	S065X9S	22
Trauma	10	S73015A	28
Trauma	10	S61254A	6
Trauma	10	S83511D	5
Trauma	10	S83512D	6
Trauma	10	S83511A	118
Trauma	10	S82202A	154
Trauma	10	S56127A	1
Trauma	10	S22050A	40
Trauma	10	S064X0A	1242
Trauma	10	S82231A	79
Trauma	10	S0219XA	1471
Trauma	10	S82251B	66
Trauma	10	S82222A	54
Trauma	10	S52202A	45
Trauma	10	S42451A	370

© 2021 Pelletier et al. *JAMA Network Open.*

Diagnosis	ICD Version	ICD Code	Number of Encounters
Trauma	10	S36521A	4
Trauma	10	S72321A	645
Trauma	10	S061X1A	15
Trauma	10	S79121A	213
Trauma	10	S36290A	6
Trauma	10	S72492A	93
Trauma	10	S89042A	31
Trauma	10	S22040A	16
Trauma	10	S82151A	323
Trauma	10	S89132A	33
Trauma	10	S89021A	58
Trauma	10	S0990XA	386
Trauma	10	S301XXA	187
Trauma	10	S22079A	25
Trauma	10	S42351A	17
Trauma	10	S81021A	27
Trauma	10	S43101A	6
Trauma	10	S36032A	609
Trauma	10	S72001A	159
Trauma	10	S82241A	99

© 2021 Pelletier et al. *JAMA Network Open.*

Diagnosis	ICD Version	ICD Code	Number of Encounters
Trauma	10	S0291XA	90
Trauma	10	S82302A	124
Trauma	10	S36115A	388
Trauma	10	S52572A	9
Trauma	10	S82141A	95
Trauma	10	T183XXA	499
Trauma	10	S61326A	1
Trauma	10	S79122A	176
Trauma	10	S0012XA	13
Trauma	10	S42412A	3169
Trauma	10	S42422A	287
Trauma	10	S36031A	667
Trauma	10	S92351A	5
Trauma	10	S0232XA	208
Trauma	10	S82871G	1
Trauma	10	S82871A	37
Trauma	10	S52231B	14
Trauma	10	S27321A	148
Trauma	10	S52132A	68
Trauma	10	S82101A	48

© 2021 Pelletier et al. *JAMA Network Open.*

Diagnosis	ICD Version	ICD Code	Number of Encounters
Trauma	10	S82101D	2
Trauma	10	S32810A	126
Trauma	10	S83095A	3
Trauma	10	S59231A	3
Trauma	10	S52021A	61
Trauma	10	S86822A	13
Trauma	10	S82252B	79
Trauma	10	S61402A	10
Trauma	10	S63269A	1
Trauma	10	S8392XA	5
Trauma	10	S72352A	306
Trauma	10	S01512A	180
Trauma	10	S060X9A	850
Trauma	10	S022XXA	237
Trauma	10	S02611A	60
Trauma	10	S72342A	619
Trauma	10	S062X9A	421
Trauma	10	T18198A	1584
Trauma	10	S82301A	112
Trauma	10	S92311A	18

© 2021 Pelletier et al. *JAMA Network Open.*

Diagnosis	ICD Version	ICD Code	Number of Encounters
Trauma	10	S82202C	18
Trauma	10	S82202J	2
Trauma	10	S52301B	92
Trauma	10	S066X9A	746
Trauma	10	S060X1A	718
Trauma	10	S3609XA	7
Trauma	10	S42411A	2093
Trauma	10	S8251XA	64
Trauma	10	S72491A	86
Trauma	10	T189XXA	230
Trauma	10	S299XXA	11
Trauma	10	S52301A	100
Trauma	10	S52201A	28
Trauma	10	S42302A	54
Trauma	10	S52302B	142
Trauma	10	S32421A	47
Trauma	10	S51812A	59
Trauma	10	S72422A	18
Trauma	10	S21112A	10
Trauma	10	S62616A	12

© 2021 Pelletier et al. *JAMA Network Open.*

Diagnosis	ICD Version	ICD Code	Number of Encounters
Trauma	10	S0242XA	66
Trauma	10	S82852A	57
Trauma	10	S42442A	97
Trauma	10	S065X0A	1899
Trauma	10	S82832A	33
Trauma	10	T1490XA	15
Trauma	10	S52502B	136
Trauma	10	S92251A	5
Trauma	10	S72322A	627
Trauma	10	S81011S	1
Trauma	10	S020XXA	4635
Trauma	10	S0240CB	6
Trauma	10	S02672A	12
Trauma	10	S72332A	490
Trauma	10	S37019A	3
Trauma	10	S72324A	29
Trauma	10	S72391A	96
Trauma	10	S41151A	14
Trauma	10	T182XXA	814
Trauma	10	S42441A	79

© 2021 Pelletier et al. *JAMA Network Open.*

Diagnosis	ICD Version	ICD Code	Number of Encounters
Trauma	10	S52571A	9
Trauma	10	S0282XA	89
Trauma	10	S41112A	31
Trauma	10	T17990A	225
Trauma	10	S42351B	15
Trauma	10	S82201A	158
Trauma	10	S5292XE	1
Trauma	10	S52601A	18
Trauma	10	S0102XA	28
Trauma	10	S52302A	102
Trauma	10	S82831A	44
Trauma	10	S42422B	26
Trauma	10	S86021A	14
Trauma	10	S82292A	48
Trauma	10	S1083XA	13
Trauma	10	S0265XA	43
Trauma	10	S82841A	145
Trauma	10	S89129A	1
Trauma	10	S82851A	53
Trauma	10	S82011A	12

© 2021 Pelletier et al. *JAMA Network Open.*

Diagnosis	ICD Version	ICD Code	Number of Encounters
Trauma	10	S02622A	43
Trauma	10	S32041A	15
Trauma	10	S62620B	9
Trauma	10	S6411XA	7
Trauma	10	S270XXA	538
Trauma	10	S52031A	31
Trauma	10	S0261XA	45
Trauma	10	S36030A	63
Trauma	10	S39012A	33
Trauma	10	S065X1A	334
Trauma	10	S52502A	168
Trauma	10	S89041A	24
Trauma	10	S83014A	9
Trauma	10	S3981XA	47
Trauma	10	S72402A	135
Trauma	10	S61220A	2
Trauma	10	S92511B	18
Trauma	10	S0181XA	327
Trauma	10	S83281A	20
Trauma	10	S6732XA	1

© 2021 Pelletier et al. *JAMA Network Open.*

**Diagnosis**	**ICD Version**	**ICD Code**	**Number of Encounters**
Trauma	10	S82891A	36
Trauma	10	S52351B	20
Trauma	10	S82201B	62
Trauma	10	S89142A	68
Trauma	10	S36116A	567
Trauma	10	S42021A	61
Trauma	10	S32592A	87
Trauma	10	S0101XA	197
Trauma	10	S30811A	48
Trauma	10	S31135A	1
Trauma	10	S89141A	85
Trauma	10	S89192A	4
Trauma	10	S32434A	2
Trauma	10	S0266XA	125
Trauma	10	S85171A	5
Trauma	10	S82251A	125
Trauma	10	S066X0A	985
Trauma	10	S72002A	193
Trauma	10	S52551B	6
Trauma	10	S02652B	27

© 2021 Pelletier et al. *JAMA Network Open.*

Diagnosis	ICD Version	ICD Code	Number of Encounters
Trauma	10	S3722XA	6
Trauma	10	S06329A	25
Trauma	10	S21341A	12
Trauma	10	S06319A	16
Trauma	10	S52201B	70
Trauma	10	S01111A	162
Trauma	10	S82842A	120
Trauma	10	S3141XA	310
Trauma	10	S36039A	172
Trauma	10	S42411B	74
Trauma	10	S36400A	5
Trauma	10	T184XXA	352
Trauma	10	S41142A	3
Trauma	10	S5401XA	7
Trauma	10	S76012A	7
Trauma	10	S22019A	17
Trauma	10	S36260A	12
Trauma	10	T17590A	93
Trauma	10	S062X0A	177
Trauma	10	S53005A	1

© 2021 Pelletier et al. *JAMA Network Open.*

Diagnosis	ICD Version	ICD Code	Number of Encounters
Trauma	10	S32492A	16
Trauma	10	S42421A	209
Trauma	10	S0081XA	123
Trauma	10	S63261A	4
Trauma	10	S82042A	17
Trauma	10	S82221A	82
Trauma	10	S82242A	68
Trauma	10	S82232B	23
Trauma	10	S72331A	468
Trauma	10	S42291A	25
Trauma	10	S82142B	2
Trauma	10	S128XXA	27
Trauma	10	S72141A	71
Trauma	10	S020XXB	547
Trauma	10	S62635B	16
Trauma	10	S2231XA	35
Trauma	10	S86221A	5
Trauma	10	S72041A	19
Trauma	10	S92062D	1
Trauma	10	S89131A	37

© 2021 Pelletier et al. *JAMA Network Open.*

Diagnosis	ICD Version	ICD Code	Number of Encounters
Trauma	10	S3131XA	21
Trauma	10	S0083XA	261
Trauma	10	S7001XA	19
Trauma	10	S36114A	110
Trauma	10	S52502P	7
Trauma	10	S42492A	45
Trauma	10	S42447A	18
Trauma	10	S0033XA	27
Trauma	10	S89092A	5
Trauma	10	S42321A	20
Trauma	10	S89122A	127
Trauma	10	S1989XA	25
Trauma	10	S42491B	17
Trauma	10	S73192A	20
Trauma	10	S31113A	25
Trauma	10	S81011A	69
Trauma	10	S42202A	33
Trauma	10	S72401A	164
Trauma	10	S01412A	58
Trauma	10	S36528A	5

© 2021 Pelletier et al. *JAMA Network Open.*

Diagnosis	ICD Version	ICD Code	Number of Encounters
Trauma	10	S83282A	17
Trauma	10	S06320A	26
Trauma	10	S0003XA	274
Trauma	10	S81821A	15
Trauma	10	S82001A	13
Trauma	10	S02609A	46
Trauma	10	S3663XA	68
Trauma	10	S79131A	17
Trauma	10	S59221A	49
Trauma	10	S52252B	12
Trauma	10	S71111A	38
Trauma	10	S92341A	2
Trauma	10	S8262XB	6
Trauma	10	S52591B	26
Trauma	10	S31124A	1
Trauma	10	S82154A	7
Trauma	10	S93401A	7
Trauma	10	S270XXD	3
Trauma	10	T17920A	313
Trauma	10	S37042A	16

© 2021 Pelletier et al. *JAMA Network Open.*

Diagnosis	ICD Version	ICD Code	Number of Encounters
Trauma	10	S52501A	133
Trauma	10	S82391A	89
Trauma	10	S37041A	12
Trauma	10	S3994XD	1
Trauma	10	S3991XA	67
Trauma	10	S52102B	13
Trauma	10	S0542XA	16
Trauma	10	S062X6A	42
Trauma	10	S1193XA	6
Trauma	10	S41039A	1
Trauma	10	S0262XA	24
Trauma	10	S7222XA	379
Trauma	10	S42461A	23
Trauma	10	S0269XB	19
Trauma	10	S37062A	134
Trauma	10	S37022A	7
Trauma	10	S59122A	6
Trauma	10	S93602A	1
Trauma	10	S066X7A	76
Trauma	10	S52232B	9

© 2021 Pelletier et al. *JAMA Network Open.*

Diagnosis	ICD Version	ICD Code	Number of Encounters
Trauma	10	S02401A	46
Trauma	10	S61041A	2
Trauma	10	S02651B	30
Trauma	10	S81851A	36
Trauma	10	S86222A	3
Trauma	10	S82202B	76
Trauma	10	S02101A	15
Trauma	10	S42415A	34
Trauma	10	S27818A	14
Trauma	10	S52131A	50
Trauma	10	S91352A	3
Trauma	10	S838X1A	3
Trauma	10	S42032A	22
Trauma	10	S52592B	50
Trauma	10	S01501A	3
Trauma	10	S82232A	73
Trauma	10	S38002A	1
Trauma	10	S61521A	4
Trauma	10	S89022A	67
Trauma	10	S66321A	5

© 2021 Pelletier et al. *JAMA Network Open.*

Diagnosis	ICD Version	ICD Code	Number of Encounters
Trauma	10	S42302B	11
Trauma	10	S20419A	2
Trauma	10	S6722XA	27
Trauma	10	S52022A	73
Trauma	10	S52181B	4
Trauma	10	S82892A	24
Trauma	10	S49021P	1
Trauma	10	S31114A	25
Trauma	10	S066X1A	242
Trauma	10	S52352B	23
Trauma	10	S36420A	60
Trauma	10	S72092A	48
Trauma	10	S42491A	43
Trauma	10	S0033XD	1
Trauma	10	S52551A	6
Trauma	10	T17900A	80
Trauma	10	S60552A	8
Trauma	10	S20211A	15
Trauma	10	S79011A	226
Trauma	10	S6721XA	26

© 2021 Pelletier et al. *JAMA Network Open.*

Diagnosis	ICD Version	ICD Code	Number of Encounters
Trauma	10	S82392A	102
Trauma	10	S52272B	54
Trauma	10	S42342K	1
Trauma	10	S76312A	3
Trauma	10	S42412B	108
Trauma	10	S42413A	19
Trauma	10	S90552A	3
Trauma	10	S01522A	10
Trauma	10	S0105XA	56
Trauma	10	S82431A	12
Trauma	10	S72031A	40
Trauma	10	S065X4A	4
Trauma	10	S62631B	7
Trauma	10	S50872A	1
Trauma	10	S42442B	12
Trauma	10	S49021A	15
Trauma	10	S0521XA	125
Trauma	10	S62321A	2
Trauma	10	S0185XA	113
Trauma	10	S37012A	32

© 2021 Pelletier et al. *JAMA Network Open.*

Diagnosis	ICD Version	ICD Code	Number of Encounters
Trauma	10	S02109A	18
Trauma	10	S52231A	4
Trauma	10	S71011A	2
Trauma	10	S72452A	63
Trauma	10	S2242XA	86
Trauma	10	S01511A	98
Trauma	10	S42462A	39
Trauma	10	S22058A	21
Trauma	10	S82041A	10
Trauma	10	S02118A	104
Trauma	10	S72421A	17
Trauma	10	S72351A	356
Trauma	10	S948X2A	2
Trauma	10	S7221XA	322
Trauma	10	S52552A	9
Trauma	10	S82391B	23
Trauma	10	S61422A	8
Trauma	10	S67194A	7
Trauma	10	T17908A	138
Trauma	10	S22010A	7

© 2021 Pelletier et al. *JAMA Network Open.*

Diagnosis	ICD Version	ICD Code	Number of Encounters
Trauma	10	S83004A	10
Trauma	10	S36113A	246
Trauma	10	S82875A	2
Trauma	10	S82031A	11
Trauma	10	S52552P	1
Trauma	10	S335XXA	12
Trauma	10	S36531A	21
Trauma	10	S66821A	13
Trauma	10	S52271A	79
Trauma	10	S36229A	15
Trauma	10	S06357A	5
Trauma	10	S82224A	6
Trauma	10	S72341A	523
Trauma	10	T185XXA	74
Trauma	10	S42292A	18
Trauma	10	S82244A	11
Trauma	10	S81812A	49
Trauma	10	S838X2A	7
Trauma	10	S52531A	3
Trauma	10	S8001XA	12

© 2021 Pelletier et al. *JAMA Network Open.*

Diagnosis	ICD Version	ICD Code	Number of Encounters
Trauma	10	S028XXA	73
Trauma	10	S2222XA	4
Trauma	10	S73001A	129
Trauma	10	S53124A	4
Trauma	10	S62351B	2
Trauma	10	S36029A	11
Trauma	10	S82302B	34
Trauma	10	S199XXA	63
Trauma	10	S49002A	2
Trauma	10	S61244A	1
Trauma	10	S42448A	26
Trauma	10	S2223XA	5
Trauma	10	S02413A	12
Trauma	10	S82191A	29
Trauma	10	S92351B	4
Trauma	10	S83104A	5
Trauma	10	S01452A	123
Trauma	10	S06300A	67
Trauma	10	S32050A	7
Trauma	10	S92532B	3

© 2021 Pelletier et al. *JAMA Network Open.*

Diagnosis	ICD Version	ICD Code	Number of Encounters
Trauma	10	S0264XA	14
Trauma	10	S89002A	13
Trauma	10	S022XXB	43
Trauma	10	S61219A	1
Trauma	10	S52122A	31
Trauma	10	S52302C	7
Trauma	10	S72321B	18
Trauma	10	S3023XA	25
Trauma	10	S02402A	40
Trauma	10	S02119B	31
Trauma	10	S52272A	101
Trauma	10	T162XXA	5
Trauma	10	S31040A	6
Trauma	10	S0242XB	34
Trauma	10	S72325A	23
Trauma	10	S3512XA	2
Trauma	10	S069X9D	61
Trauma	10	S22021A	2
Trauma	10	S63114A	4
Trauma	10	S00532A	10

© 2021 Pelletier et al. *JAMA Network Open.*

Diagnosis	ICD Version	ICD Code	Number of Encounters
Trauma	10	S065X0D	102
Trauma	10	S72301A	503
Trauma	10	S82241D	2
Trauma	10	T17890A	41
Trauma	10	S06330A	10
Trauma	10	S0921XA	7
Trauma	10	S37812A	15
Trauma	10	S72351B	33
Trauma	10	S0240DB	5
Trauma	10	S91002A	16
Trauma	10	S72011A	14
Trauma	10	S8992XA	10
Trauma	10	S92422B	30
Trauma	10	S42201A	42
Trauma	10	S02652A	84
Trauma	10	S86912A	3
Trauma	10	S0993XA	16
Trauma	10	S72471A	11
Trauma	10	S069X5D	3
Trauma	10	S82102A	61

© 2021 Pelletier et al. *JAMA Network Open.*

Diagnosis	ICD Version	ICD Code	Number of Encounters
Trauma	10	S1195XA	10
Trauma	10	S13150A	3
Trauma	10	S76111A	13
Trauma	10	S72042A	34
Trauma	10	S22048A	6
Trauma	10	S272XXA	223
Trauma	10	S065X2S	1
Trauma	10	S73005D	7
Trauma	10	S36232A	19
Trauma	10	S2241XA	70
Trauma	10	S06341A	18
Trauma	10	S31143A	6
Trauma	10	S62647B	1
Trauma	10	S52032A	27
Trauma	10	S01452S	4
Trauma	10	S72361A	17
Trauma	10	S22020A	10
Trauma	10	S82002A	22
Trauma	10	S0210XA	16
Trauma	10	S90851A	20

© 2021 Pelletier et al. *JAMA Network Open.*

Diagnosis	ICD Version	ICD Code	Number of Encounters
Trauma	10	S72345A	68
Trauma	10	S53105A	6
Trauma	10	T17528A	207
Trauma	10	S42022A	78
Trauma	10	S82112A	14
Trauma	10	S01112A	162
Trauma	10	S62637B	9
Trauma	10	S0512XA	73
Trauma	10	T17998A	128
Trauma	10	S31119A	43
Trauma	10	S96191A	2
Trauma	10	S0100XA	16
Trauma	10	S0912XA	26
Trauma	10	S42414A	32
Trauma	10	S81012A	76
Trauma	10	S06339A	23
Trauma	10	S065X9D	206
Trauma	10	S62623B	5
Trauma	10	S92512B	11
Trauma	10	S62347B	1

© 2021 Pelletier et al. *JAMA Network Open.*

Diagnosis	ICD Version	ICD Code	Number of Encounters
Trauma	10	T18108A	113
Trauma	10	S59222P	2
Trauma	10	S62633B	21
Trauma	10	T17228A	42
Trauma	10	S82142A	79
Trauma	10	S82192A	32
Trauma	10	S66022A	2
Trauma	10	S92212A	4
Trauma	10	S06372A	2
Trauma	10	S062X1D	7
Trauma	10	S98121A	38
Trauma	10	T18120A	15
Trauma	10	S300XXA	42
Trauma	10	S80862A	11
Trauma	10	S92211A	6
Trauma	10	S066X0S	3
Trauma	10	S29011A	3
Trauma	10	S062X9D	175
Trauma	10	T25222A	105
Trauma	10	S83211A	3

© 2021 Pelletier et al. *JAMA Network Open.*

Diagnosis	ICD Version	ICD Code	Number of Encounters
Trauma	10	S06340A	79
Trauma	10	S02611B	9
Trauma	10	S92412B	15
Trauma	10	S72302A	489
Trauma	10	S070XXA	1
Trauma	10	S52271B	41
Trauma	10	S1121XA	31
Trauma	10	S069X9A	320
Trauma	10	S96122A	8
Trauma	10	S032XXA	20
Trauma	10	S21119A	6
Trauma	10	S42442K	4
Trauma	10	S21211A	6
Trauma	10	S36251A	4
Trauma	10	S62634P	1
Trauma	10	S42453A	5
Trauma	10	S72045A	7
Trauma	10	S069X1A	189
Trauma	10	S5422XA	2
Trauma	10	S49122A	6

© 2021 Pelletier et al. *JAMA Network Open.*

Diagnosis	ICD Version	ICD Code	Number of Encounters
Trauma	10	S62626B	3
Trauma	10	T17520A	201
Trauma	10	S0122XA	1
Trauma	10	S62640B	1
Trauma	10	S065X6A	25
Trauma	10	S79192A	20
Trauma	10	S01122A	5
Trauma	10	S52002B	5
Trauma	10	S8262XC	2
Trauma	10	S45111A	18
Trauma	10	S37052A	105
Trauma	10	S37002A	7
Trauma	10	S62522A	1
Trauma	10	S91321A	19
Trauma	10	S12110A	40
Trauma	10	S12110K	6
Trauma	10	S92321A	13
Trauma	10	T17598A	131
Trauma	10	S0303XA	8
Trauma	10	T17428A	21

© 2021 Pelletier et al. *JAMA Network Open.*

Diagnosis	ICD Version	ICD Code	Number of Encounters
Trauma	10	S06331A	4
Trauma	10	T17220A	18
Trauma	10	S1191XA	32
Trauma	10	S13111A	44
Trauma	10	S49121A	10
Trauma	10	S0219XB	117
Trauma	10	S42412P	10
Trauma	10	S62630B	17
Trauma	10	S72392A	94
Trauma	10	S97121A	2
Trauma	10	S67190A	11
Trauma	10	S01532A	19
Trauma	10	S20429A	1
Trauma	10	S0240DA	38
Trauma	10	S62512B	9
Trauma	10	S82161A	3
Trauma	10	T17928A	103
Trauma	10	S0889XA	2
Trauma	10	S70351A	3
Trauma	10	S59212P	1

© 2021 Pelletier et al. *JAMA Network Open.*

Diagnosis	ICD Version	ICD Code	Number of Encounters
Trauma	10	S066X9D	78
Trauma	10	S32391A	20
Trauma	10	S40011A	8
Trauma	10	S162XXA	9
Trauma	10	S29012A	4
Trauma	10	S62647A	1
Trauma	10	S98122A	27
Trauma	10	S80861A	13
Trauma	10	T17320A	25
Trauma	10	S0452XS	3
Trauma	10	S140XXA	24
Trauma	10	T17898A	29
Trauma	10	S35292A	2
Trauma	10	T17298A	52
Trauma	10	S36261A	27
Trauma	10	S0240FS	1
Trauma	10	S62614A	3
Trauma	10	S82441A	5
Trauma	10	S82241K	1
Trauma	10	T2121XA	440

© 2021 Pelletier et al. *JAMA Network Open.*

Diagnosis	ICD Version	ICD Code	Number of Encounters
Trauma	10	S68110A	11
Trauma	10	S83251A	5
Trauma	10	S065X3A	11
Trauma	10	S065X3D	2
Trauma	10	S0211GA	102
Trauma	10	S36438A	337
Trauma	10	T17910A	30
Trauma	10	S0182XA	25
Trauma	10	S41111A	12
Trauma	10	S82155A	10
Trauma	10	S01422A	5
Trauma	10	S8264XB	4
Trauma	10	S86011A	2
Trauma	10	S52601B	13
Trauma	10	S90852A	21
Trauma	10	S73004A	72
Trauma	10	S27322A	143
Trauma	10	S3219XA	19
Trauma	10	S023XXB	2
Trauma	10	S02401B	7

© 2021 Pelletier et al. *JAMA Network Open.*

Diagnosis	ICD Version	ICD Code	Number of Encounters
Trauma	10	S72344A	58
Trauma	10	S52352A	10
Trauma	10	S73002A	122
Trauma	10	S36112A	31
Trauma	10	S00461A	5
Trauma	10	S066X1D	7
Trauma	10	S7224XA	9
Trauma	10	S42024A	14
Trauma	10	S06891D	4
Trauma	10	S0121XA	29
Trauma	10	S53014A	2
Trauma	10	S32038A	35
Trauma	10	S90444A	9
Trauma	10	S62632B	18
Trauma	10	S92114A	2
Trauma	10	S62602B	1
Trauma	10	S42412D	4
Trauma	10	T1582XA	3
Trauma	10	S065X7A	91
Trauma	10	S3120XA	4

© 2021 Pelletier et al. *JAMA Network Open.*

Diagnosis	ICD Version	ICD Code	Number of Encounters
Trauma	10	S68122A	13
Trauma	10	S49191A	6
Trauma	10	S81042A	24
Trauma	10	S06350A	58
Trauma	10	S748X2A	1
Trauma	10	S1181XA	24
Trauma	10	S9032XA	5
Trauma	10	S52321B	44
Trauma	10	S6421XA	5
Trauma	10	S62625B	12
Trauma	10	S32512A	66
Trauma	10	S42451K	3
Trauma	10	S32048A	25
Trauma	10	S00552A	1
Trauma	10	S52512B	3
Trauma	10	S20212A	15
Trauma	10	S62301B	3
Trauma	10	S63115A	6
Trauma	10	S5292XB	43
Trauma	10	S63285A	1

© 2021 Pelletier et al. *JAMA Network Open.*

Diagnosis	ICD Version	ICD Code	Number of Encounters
Trauma	10	S72142A	88
Trauma	10	S1123XA	21
Trauma	10	S0265XB	15
Trauma	10	S1122XA	7
Trauma	10	S06379A	21
Trauma	10	S76121A	18
Trauma	10	S82111A	22
Trauma	10	S52322A	70
Trauma	10	S92214A	2
Trauma	10	S72455A	5
Trauma	10	T17918A	52
Trauma	10	S06387A	5
Trauma	10	S6742XA	3
Trauma	10	S01552A	12
Trauma	10	S82451A	21
Trauma	10	S42142B	1
Trauma	10	S82432A	12
Trauma	10	S52322B	55
Trauma	10	S66322A	3
Trauma	10	S32048B	2

© 2021 Pelletier et al. *JAMA Network Open.*

Diagnosis	ICD Version	ICD Code	Number of Encounters
Trauma	10	S06899A	35
Trauma	10	S82291A	36
Trauma	10	S2002XA	2
Trauma	10	S5291XA	23
Trauma	10	S42344A	11
Trauma	10	S36118A	7
Trauma	10	S82301B	40
Trauma	10	S68125A	7
Trauma	10	S22068A	19
Trauma	10	S0291XD	8
Trauma	10	S062X0D	41
Trauma	10	S20369A	1
Trauma	10	S31831A	46
Trauma	10	S52222B	14
Trauma	10	S62613A	6
Trauma	10	S62627B	3
Trauma	10	T17490A	57
Trauma	10	S39848A	19
Trauma	10	S73001D	9
Trauma	10	S00462A	2

© 2021 Pelletier et al. *JAMA Network Open.*

Diagnosis	ICD Version	ICD Code	Number of Encounters
Trauma	10	S52391B	13
Trauma	10	T171XXA	27
Trauma	10	S62621B	7
Trauma	10	S79141A	19
Trauma	10	S72352B	45
Trauma	10	S72472A	18
Trauma	10	S2611XA	5
Trauma	10	S73191A	29
Trauma	10	S080XXA	61
Trauma	10	S36490A	7
Trauma	10	S32445A	3
Trauma	10	S0522XA	103
Trauma	10	S72044A	5
Trauma	10	S62612A	4
Trauma	10	S56421A	1
Trauma	10	S73101A	8
Trauma	10	S01419A	3
Trauma	10	S01451A	116
Trauma	10	T17820A	63
Trauma	10	S68111A	14

© 2021 Pelletier et al. *JAMA Network Open.*

Diagnosis	ICD Version	ICD Code	Number of Encounters
Trauma	10	S67192A	14
Trauma	10	S27391A	5
Trauma	10	S86022A	16
Trauma	10	S73034A	3
Trauma	10	S062X7A	63
Trauma	10	S98222A	6
Trauma	10	S728X1A	24
Trauma	10	S52232M	2
Trauma	10	S5291XP	2
Trauma	10	S52591A	26
Trauma	10	S06323A	1
Trauma	10	S37051A	70
Trauma	10	S39021A	5
Trauma	10	S92215A	3
Trauma	10	S61212A	10
Trauma	10	S6702XA	7
Trauma	10	S0911XA	1
Trauma	10	S92324A	2
Trauma	10	S63286A	3
Trauma	10	S72335A	34

© 2021 Pelletier et al. *JAMA Network Open.*

Diagnosis	ICD Version	ICD Code	Number of Encounters
Trauma	10	S8782XA	9
Trauma	10	S32029A	56
Trauma	10	S01411A	60
Trauma	10	S3633XA	14
Trauma	10	S0281XA	98
Trauma	10	S72334A	25
Trauma	10	S61451A	83
Trauma	10	S32301A	31
Trauma	10	S91341A	39
Trauma	10	S73005A	79
Trauma	10	S89032A	17
Trauma	10	S7002XA	16
Trauma	10	T22231A	56
Trauma	10	S90862A	4
Trauma	10	S82225A	6
Trauma	10	T17500A	6
Trauma	10	S52031C	1
Trauma	10	T192XXA	24
Trauma	10	S52502C	5
Trauma	10	S62635A	4

© 2021 Pelletier et al. *JAMA Network Open.*

Diagnosis	ICD Version	ICD Code	Number of Encounters
Trauma	10	S68620A	12
Trauma	10	S83212A	2
Trauma	10	S60361A	2
Trauma	10	S06820A	3
Trauma	10	S62326A	4
Trauma	10	S60424A	1
Trauma	10	S062X5A	11
Trauma	10	S062X5S	1
Trauma	10	S53144A	1
Trauma	10	S01151A	55
Trauma	10	S21111A	13
Trauma	10	S32311A	11
Trauma	10	S82402B	3
Trauma	10	T17498A	26
Trauma	10	S42025A	16
Trauma	10	S240XXA	7
Trauma	10	S70352A	1
Trauma	10	S12500A	45
Trauma	10	S0266XB	84
Trauma	10	S062X1A	96

© 2021 Pelletier et al. *JAMA Network Open.*

Diagnosis	ICD Version	ICD Code	Number of Encounters
Trauma	10	S37011A	24
Trauma	10	S12100A	43
Trauma	10	S31622A	1
Trauma	10	S82422A	4
Trauma	10	S52291B	12
Trauma	10	S89302A	2
Trauma	10	S63015A	2
Trauma	10	S8252XA	44
Trauma	10	S83521A	4
Trauma	10	S01351A	11
Trauma	10	S68625A	6
Trauma	10	S13110A	10
Trauma	10	S52031B	3
Trauma	10	S53031A	2
Trauma	10	S90445A	6
Trauma	10	S68126A	16
Trauma	10	S36231A	23
Trauma	10	S62515B	2
Trauma	10	S67191A	6
Trauma	10	S49111A	3

© 2021 Pelletier et al. *JAMA Network Open.*

Diagnosis	ICD Version	ICD Code	Number of Encounters
Trauma	10	S62512A	7
Trauma	10	S62656B	1
Trauma	10	S62632A	5
Trauma	10	S20319A	6
Trauma	10	S82201C	8
Trauma	10	S37031A	74
Trauma	10	T280XXA	7
Trauma	10	S37032A	78
Trauma	10	S82872A	31
Trauma	10	S36892A	53
Trauma	10	S0240CA	48
Trauma	10	S12601A	12
Trauma	10	S52235B	1
Trauma	10	S7011XA	45
Trauma	10	S72352K	2
Trauma	10	S31821A	15
Trauma	10	S42424A	4
Trauma	10	S22018A	12
Trauma	10	S42431A	12
Trauma	10	S42411D	4

© 2021 Pelletier et al. *JAMA Network Open.*

Diagnosis	ICD Version	ICD Code	Number of Encounters
Trauma	10	S72362C	3
Trauma	10	S68113A	5
Trauma	10	T17308A	15
Trauma	10	S52522A	20
Trauma	10	S82302D	4
Trauma	10	S82392B	35
Trauma	10	S72354A	9
Trauma	10	S52272P	6
Trauma	10	S0001XA	17
Trauma	10	S14109A	27
Trauma	10	S98111A	10
Trauma	10	S42342A	17
Trauma	10	S02671B	3
Trauma	10	S92322B	8
Trauma	10	S9304XD	1
Trauma	10	S24112A	24
Trauma	10	S22031A	2
Trauma	10	S76021A	1
Trauma	10	S72411A	2
Trauma	10	S6710XA	1

© 2021 Pelletier et al. *JAMA Network Open.*

Diagnosis	ICD Version	ICD Code	Number of Encounters
Trauma	10	S76222A	3
Trauma	10	S82842B	16
Trauma	10	S02601B	22
Trauma	10	S81811A	58
Trauma	10	S52322P	3
Trauma	10	T18190A	54
Trauma	10	S8262XA	30
Trauma	10	S68120A	15
Trauma	10	S82891P	1
Trauma	10	S36252A	6
Trauma	10	S02612A	101
Trauma	10	S8002XA	13
Trauma	10	S56221A	5
Trauma	10	S68626A	8
Trauma	10	S79111A	25
Trauma	10	S82301C	13
Trauma	10	S64490A	9
Trauma	10	S62330B	1
Trauma	10	S62626A	2
Trauma	10	S53125A	7

© 2021 Pelletier et al. *JAMA Network Open.*

Diagnosis	ICD Version	ICD Code	Number of Encounters
Trauma	10	S62511B	6
Trauma	10	S29021A	2
Trauma	10	S20312A	9
Trauma	10	S52532B	4
Trauma	10	S72125A	1
Trauma	10	S46921A	1
Trauma	10	S82092A	30
Trauma	10	S62610B	13
Trauma	10	T25292A	25
Trauma	10	S42472A	17
Trauma	10	S27803A	19
Trauma	10	S62634B	20
Trauma	10	T25331A	3
Trauma	10	S2243XA	39
Trauma	10	S31611A	4
Trauma	10	S92115A	1
Trauma	10	S32018A	45
Trauma	10	S82011P	1
Trauma	10	S73014A	43
Trauma	10	S12001A	6

© 2021 Pelletier et al. *JAMA Network Open.*

Diagnosis	ICD Version	ICD Code	Number of Encounters
Trauma	10	S72111A	7
Trauma	10	S72101A	6
Trauma	10	S82234A	6
Trauma	10	S3210XA	45
Trauma	10	S81051A	5
Trauma	10	S31643A	13
Trauma	10	S06309A	34
Trauma	10	S61250A	18
Trauma	10	S0011XA	19
Trauma	10	S0232XB	17
Trauma	10	S52021C	2
Trauma	10	S02121A	14
Trauma	10	S01451D	4
Trauma	10	S82251C	30
Trauma	10	S36898A	16
Trauma	10	S82421A	8
Trauma	10	T2162XA	2
Trauma	10	S52352C	4
Trauma	10	S42031A	14
Trauma	10	S9001XA	4

© 2021 Pelletier et al. *JAMA Network Open.*

Diagnosis	ICD Version	ICD Code	Number of Encounters
Trauma	10	S3282XA	50
Trauma	10	S62326B	2
Trauma	10	S100XXA	13
Trauma	10	S06360A	45
Trauma	10	S36428A	21
Trauma	10	S06361A	15
Trauma	10	S42425A	3
Trauma	10	S52572P	1
Trauma	10	S32042A	7
Trauma	10	S82151D	2
Trauma	10	S82091B	4
Trauma	10	S0501XA	21
Trauma	10	S7012XA	46
Trauma	10	S82142P	2
Trauma	10	S098XXA	80
Trauma	10	S51822A	13
Trauma	10	S42201P	1
Trauma	10	S00432A	11
Trauma	10	S90415A	1
Trauma	10	S86392A	1

© 2021 Pelletier et al. *JAMA Network Open.*

Diagnosis	ICD Version	ICD Code	Number of Encounters
Trauma	10	S63650A	1
Trauma	10	S62364B	2
Trauma	10	S066X0D	34
Trauma	10	S83411A	2
Trauma	10	S13141A	5
Trauma	10	S82232K	2
Trauma	10	S52561A	5
Trauma	10	S065X6D	8
Trauma	10	S59241A	2
Trauma	10	S0269XA	31
Trauma	10	S93492A	4
Trauma	10	S83421A	6
Trauma	10	S92312A	14
Trauma	10	T1490	9
Trauma	10	S062X0S	7
Trauma	10	S83272A	2
Trauma	10	S81832A	8
Trauma	10	S62332A	1
Trauma	10	S82012A	13
Trauma	10	S51022A	5

© 2021 Pelletier et al. *JAMA Network Open.*

Diagnosis	ICD Version	ICD Code	Number of Encounters
Trauma	10	S0240FA	34
Trauma	10	S62366B	1
Trauma	10	S82891B	8
Trauma	10	S83012A	4
Trauma	10	S92015A	1
Trauma	10	S83195A	10
Trauma	10	S3802XA	2
Trauma	10	S76911A	3
Trauma	10	S72362A	11
Trauma	10	S62314A	2
Trauma	10	S12000D	6
Trauma	10	S12100D	2
Trauma	10	S52371A	7
Trauma	10	S52102P	2
Trauma	10	S62011A	2
Trauma	10	S72431A	15
Trauma	10	S52392B	18
Trauma	10	S52125A	4
Trauma	10	S330XXA	1
Trauma	10	S82252K	5

© 2021 Pelletier et al. *JAMA Network Open.*

Diagnosis	ICD Version	ICD Code	Number of Encounters
Trauma	10	S86892A	3
Trauma	10	S025XXB	5
Trauma	10	S42352A	24
Trauma	10	S31812A	3
Trauma	10	S02642A	21
Trauma	10	S89321A	17
Trauma	10	S3739XA	23
Trauma	10	S39013A	2
Trauma	10	S92352K	1
Trauma	10	S75012A	8
Trauma	10	S66325A	4
Trauma	10	S62321B	5
Trauma	10	S32454A	3
Trauma	10	S83241A	8
Trauma	10	S59221P	1
Trauma	10	S42112A	8
Trauma	10	S68624A	5
Trauma	10	S72409A	3
Trauma	10	S62662B	3
Trauma	10	S93411A	1

© 2021 Pelletier et al. *JAMA Network Open.*

Diagnosis	ICD Version	ICD Code	Number of Encounters
Trauma	10	S56522A	3
Trauma	10	S52101A	14
Trauma	10	S62232A	5
Trauma	10	S52521A	14
Trauma	10	S8411XD	1
Trauma	10	S89111A	4
Trauma	10	S73192D	2
Trauma	10	S43432A	3
Trauma	10	S43225A	9
Trauma	10	S92421B	31
Trauma	10	S52602A	15
Trauma	10	S82291H	2
Trauma	10	S37061A	137
Trauma	10	S32032A	8
Trauma	10	S069X1D	7
Trauma	10	S79001A	8
Trauma	10	S32392A	26
Trauma	10	S72351C	17
Trauma	10	S21332A	5
Trauma	10	S066X4A	4

© 2021 Pelletier et al. *JAMA Network Open.*

Diagnosis	ICD Version	ICD Code	Number of Encounters
Trauma	10	T17408A	13
Trauma	10	S02670A	4
Trauma	10	S52692A	5
Trauma	10	S065X5A	17
Trauma	10	S79102A	20
Trauma	10	S06891A	16
Trauma	10	S21141A	8
Trauma	10	S33111A	1
Trauma	10	S5291XB	24
Trauma	10	S52324B	1
Trauma	10	S069X0D	30
Trauma	10	S73002S	4
Trauma	10	S82392C	9
Trauma	10	S1185XA	14
Trauma	10	S02600A	11
Trauma	10	T2102XA	27
Trauma	10	S79101A	29
Trauma	10	S32302A	32
Trauma	10	S82831B	8
Trauma	10	S20309A	3

© 2021 Pelletier et al. *JAMA Network Open.*

Diagnosis	ICD Version	ICD Code	Number of Encounters
Trauma	10	S68115A	7
Trauma	10	S68124A	10
Trauma	10	S32811A	49
Trauma	10	S12110B	1
Trauma	10	S12111G	1
Trauma	10	S22038A	7
Trauma	10	S14123A	4
Trauma	10	S51831A	2
Trauma	10	S82841F	1
Trauma	10	S0531XA	90
Trauma	10	S73004D	4
Trauma	10	S91012A	13
Trauma	10	S32120A	6
Trauma	10	S32599A	5
Trauma	10	S32049A	20
Trauma	10	S82202P	5
Trauma	10	T24312A	92
Trauma	10	S85122A	1
Trauma	10	T22351A	16
Trauma	10	T2033XA	4

© 2021 Pelletier et al. *JAMA Network Open.*

Diagnosis	ICD Version	ICD Code	Number of Encounters
Trauma	10	T2037XA	29
Trauma	10	S72442A	15
Trauma	10	S83005A	9
Trauma	10	S72051A	11
Trauma	10	S22059A	32
Trauma	10	S32039A	36
Trauma	10	S32129A	5
Trauma	10	S52042A	5
Trauma	10	S36430A	55
Trauma	10	S36291A	5
Trauma	10	T2132XA	92
Trauma	10	S72051G	1
Trauma	10	S72001D	5
Trauma	10	S52212A	4
Trauma	10	S42001A	32
Trauma	10	S22088A	35
Trauma	10	S49131A	4
Trauma	10	S82122A	7
Trauma	10	S061X9A	76
Trauma	10	S71132A	27

© 2021 Pelletier et al. *JAMA Network Open.*

Diagnosis	ICD Version	ICD Code	Number of Encounters
Trauma	10	T24212A	147
Trauma	10	S06319D	4
Trauma	10	S0219XD	33
Trauma	10	S52101B	11
Trauma	10	S06310A	32
Trauma	10	S92101B	1
Trauma	10	S7291XA	63
Trauma	10	T24031A	2
Trauma	10	S72401B	11
Trauma	10	S72012A	14
Trauma	10	S92325B	1
Trauma	10	S72422C	4
Trauma	10	T2122XA	213
Trauma	10	S92342A	6
Trauma	10	S06349A	50
Trauma	10	S7292XA	57
Trauma	10	S02411A	24
Trauma	10	S52321A	30
Trauma	10	S069X0A	192
Trauma	10	T2000XA	36

© 2021 Pelletier et al. *JAMA Network Open.*

Diagnosis	ICD Version	ICD Code	Number of Encounters
Trauma	10	S62316B	3
Trauma	10	S22060A	28
Trauma	10	S3613XA	4
Trauma	10	T17828A	23
Trauma	10	S42212A	12
Trauma	10	T24311A	91
Trauma	10	S91322A	24
Trauma	10	T2026XA	188
Trauma	10	S1120XA	2
Trauma	10	S81842A	9
Trauma	10	S52592D	1
Trauma	10	S65012A	6
Trauma	10	S06346A	3
Trauma	10	S51811A	31
Trauma	10	S68522A	4
Trauma	10	S82831D	1
Trauma	10	S01511D	2
Trauma	10	S91001A	9
Trauma	10	S381XXA	26
Trauma	10	S27331A	34

© 2021 Pelletier et al. *JAMA Network Open.*

Diagnosis	ICD Version	ICD Code	Number of Encounters
Trauma	10	S40812A	4
Trauma	10	S14103A	4
Trauma	10	S12390A	12
Trauma	10	S41141A	4
Trauma	10	T24012A	14
Trauma	10	S14141A	1
Trauma	10	S72144A	12
Trauma	10	S3723XA	9
Trauma	10	S12112G	2
Trauma	10	S72462A	7
Trauma	10	T2126XA	41
Trauma	10	S22080A	36
Trauma	10	S098XXD	3
Trauma	10	S36532A	8
Trauma	10	S31633A	7
Trauma	10	S75022A	10
Trauma	10	T2134XA	64
Trauma	10	S064X1D	3
Trauma	10	S01551A	46
Trauma	10	S32511A	67

© 2021 Pelletier et al. *JAMA Network Open.*

Diagnosis	ICD Version	ICD Code	Number of Encounters
Trauma	10	S064X0S	3
Trauma	10	S62611B	10
Trauma	10	T22031A	2
Trauma	10	S0195XA	3
Trauma	10	T22332S	1
Trauma	10	S71122D	2
Trauma	10	S025XXA	29
Trauma	10	S06310D	1
Trauma	10	S48121A	3
Trauma	10	S76821A	5
Trauma	10	T180XXA	27
Trauma	10	S45101A	1
Trauma	10	S72302D	14
Trauma	10	S02412A	28
Trauma	10	S061X7A	68
Trauma	10	S52372A	10
Trauma	10	S02642B	12
Trauma	10	S01452D	5
Trauma	10	T25311A	13
Trauma	10	S22051A	12

© 2021 Pelletier et al. *JAMA Network Open.*

Diagnosis	ICD Version	ICD Code	Number of Encounters
Trauma	10	S82291K	2
Trauma	10	T2029XA	128
Trauma	10	T24211A	111
Trauma	10	S81022A	17
Trauma	10	T2601XA	5
Trauma	10	T23201A	58
Trauma	10	S0211HA	115
Trauma	10	T23351A	17
Trauma	10	S73014D	3
Trauma	10	T23391A	14
Trauma	10	T2109XA	1
Trauma	10	T2135XD	2
Trauma	10	S41152A	21
Trauma	10	S51841A	3
Trauma	10	T23361A	29
Trauma	10	T2009XA	18
Trauma	10	S0532XA	92
Trauma	10	T17420A	41
Trauma	10	T22012A	17
Trauma	10	S02602A	32

© 2021 Pelletier et al. *JAMA Network Open.*

Diagnosis	ICD Version	ICD Code	Number of Encounters
Trauma	10	S71151A	20
Trauma	10	T24332A	62
Trauma	10	T2101XA	47
Trauma	10	S32011A	59
Trauma	10	S12040A	7
Trauma	10	T23291A	22
Trauma	10	S36899A	19
Trauma	10	S34103D	4
Trauma	10	T2023XA	39
Trauma	10	S298XXA	10
Trauma	10	S32422A	39
Trauma	10	T17300A	4
Trauma	10	S24154A	3
Trauma	10	S14106A	7
Trauma	10	S383XXA	1
Trauma	10	S064X5A	4
Trauma	10	S72343A	3
Trauma	10	T17920D	7
Trauma	10	S14155A	9
Trauma	10	T2037XS	1

© 2021 Pelletier et al. *JAMA Network Open.*

Diagnosis	ICD Version	ICD Code	Number of Encounters
Trauma	10	S15312A	1
Trauma	10	T23272A	6
Trauma	10	T2200XA	9
Trauma	10	S75021A	8
Trauma	10	S12101A	15
Trauma	10	S61401A	6
Trauma	10	T1590XA	1
Trauma	10	T33821A	3
Trauma	10	S36222A	4
Trauma	10	S42221A	2
Trauma	10	S72091A	30
Trauma	10	T2230XA	44
Trauma	10	T23262A	32
Trauma	10	S92002B	14
Trauma	10	T17398A	24
Trauma	10	S32019A	63
Trauma	10	S020XXS	7
Trauma	10	S3140XA	3
Trauma	10	S42401A	44
Trauma	10	S32010A	44

© 2021 Pelletier et al. *JAMA Network Open.*

Diagnosis	ICD Version	ICD Code	Number of Encounters
Trauma	10	T23021A	1
Trauma	10	T2005XA	6
Trauma	10	T2020XA	202
Trauma	10	T17208A	6
Trauma	10	S72302C	5
Trauma	10	S72454A	2
Trauma	10	T2129XA	7
Trauma	10	S31640A	1
Trauma	10	T286XXA	15
Trauma	10	S72002D	4
Trauma	10	S42211A	10
Trauma	10	S98221A	13
Trauma	10	S36439A	29
Trauma	10	S72032A	39
Trauma	10	S27813A	2
Trauma	10	S32414A	3
Trauma	10	S42202B	8
Trauma	10	T2133XA	31
Trauma	10	T22332A	36
Trauma	10	T281XXA	6

© 2021 Pelletier et al. *JAMA Network Open.*

Diagnosis	ICD Version	ICD Code	Number of Encounters
Trauma	10	S86821A	8
Trauma	10	T2131XA	175
Trauma	10	S01152A	45
Trauma	10	S31824A	7
Trauma	10	S71102A	18
Trauma	10	T24391A	10
Trauma	10	T2030XA	26
Trauma	10	T23061A	3
Trauma	10	T22312A	47
Trauma	10	T23302D	1
Trauma	10	S68114A	4
Trauma	10	T2039XA	34
Trauma	10	S14135A	3
Trauma	10	T25291A	21
Trauma	10	T25321A	68
Trauma	10	S2610XA	2
Trauma	10	S52562A	3
Trauma	10	S92252B	4
Trauma	10	S36538A	19
Trauma	10	S02112A	2

© 2021 Pelletier et al. *JAMA Network Open.*

Diagnosis	ICD Version	ICD Code	Number of Encounters
Trauma	10	S32022A	12
Trauma	10	S82844A	4
Trauma	10	S020XXG	10
Trauma	10	S71112A	35
Trauma	10	S01352A	21
Trauma	10	S52692B	19
Trauma	10	T270XXD	2
Trauma	10	T22311A	56
Trauma	10	S73012A	8
Trauma	10	S42002A	49
Trauma	10	T273XXA	13
Trauma	10	S14101A	9
Trauma	10	S52221A	8
Trauma	10	T2125XA	71
Trauma	10	S0552XD	1
Trauma	10	S14105A	13
Trauma	10	T22251A	43
Trauma	10	S066X3A	11
Trauma	10	S6992XA	1
Trauma	10	S14121A	2

© 2021 Pelletier et al. *JAMA Network Open.*

Diagnosis	ICD Version	ICD Code	Number of Encounters
Trauma	10	S32502A	9
Trauma	10	T23051A	10
Trauma	10	S22070A	17
Trauma	10	S79002A	8
Trauma	10	S13120A	58
Trauma	10	S22049A	15
Trauma	10	S82452B	6
Trauma	10	S22089A	46
Trauma	10	S81032A	7
Trauma	10	T23322A	7
Trauma	10	T23351D	2
Trauma	10	T2007XA	11
Trauma	10	S06321A	12
Trauma	10	S82002B	4
Trauma	10	S71112D	5
Trauma	10	T22051A	5
Trauma	10	S98012A	2
Trauma	10	S12600A	45
Trauma	10	S36498A	51
Trauma	10	S92811B	1

© 2021 Pelletier et al. *JAMA Network Open.*

Diagnosis	ICD Version	ICD Code	Number of Encounters
Trauma	10	T23241A	10
Trauma	10	S00431A	17
Trauma	10	S45192A	11
Trauma	10	S066X5A	12
Trauma	10	T2104XA	9
Trauma	10	S62603A	2
Trauma	10	T23251A	50
Trauma	10	S06316A	1
Trauma	10	S24152A	9
Trauma	10	T24011A	4
Trauma	10	T23232A	12
Trauma	10	T2106XA	7
Trauma	10	T22211A	91
Trauma	10	S52612A	3
Trauma	10	S02621B	6
Trauma	10	T20212A	7
Trauma	10	S1124XA	5
Trauma	10	S08811A	1
Trauma	10	S52371B	2
Trauma	10	T24331A	58

© 2021 Pelletier et al. *JAMA Network Open.*

Diagnosis	ICD Version	ICD Code	Number of Encounters
Trauma	10	T22311D	1
Trauma	10	S06359A	51
Trauma	10	S72001K	13
Trauma	10	S61431A	9
Trauma	10	S72341B	2
Trauma	10	T2220XA	53
Trauma	10	S72122B	1
Trauma	10	S25191A	2
Trauma	10	S08111A	6
Trauma	10	S02113A	54
Trauma	10	S12690A	22
Trauma	10	T24211D	2
Trauma	10	S069X7A	11
Trauma	10	S42402A	51
Trauma	10	S1981XA	8
Trauma	10	S72052B	1
Trauma	10	T17908D	3
Trauma	10	S72432A	12
Trauma	10	S2609XA	7
Trauma	10	S42325A	2

© 2021 Pelletier et al. *JAMA Network Open.*

Diagnosis	ICD Version	ICD Code	Number of Encounters
Trauma	10	T22041D	1
Trauma	10	T23352A	22
Trauma	10	S0125XA	21
Trauma	10	S06311A	14
Trauma	10	S72402B	8
Trauma	10	T270XXA	3
Trauma	10	T25321D	1
Trauma	10	S83094A	4
Trauma	10	T2006XA	13
Trauma	10	S91311A	36
Trauma	10	S22030A	16
Trauma	10	S066X6A	15
Trauma	10	T2036XD	1
Trauma	10	S02601A	37
Trauma	10	S08122A	9
Trauma	10	S02612B	9
Trauma	10	S52021B	6
Trauma	10	S14151A	8
Trauma	10	T2027XA	76
Trauma	10	S22069A	22

© 2021 Pelletier et al. *JAMA Network Open.*

Diagnosis	ICD Version	ICD Code	Number of Encounters
Trauma	10	T2124XA	95
Trauma	10	S76322A	3
Trauma	10	S12491A	6
Trauma	10	S79012D	2
Trauma	10	T2120XA	3
Trauma	10	S79011D	3
Trauma	10	S3669XD	1
Trauma	10	S0087XA	11
Trauma	10	S42301A	39
Trauma	10	S065X5D	9
Trauma	10	S12400A	52
Trauma	10	S34111A	1
Trauma	10	S32012A	20
Trauma	10	T23372A	5
Trauma	10	S32462A	10
Trauma	10	S72401C	8
Trauma	10	T2105XA	12
Trauma	10	S2522XA	1
Trauma	10	S61411A	20
Trauma	10	T20011A	2

© 2021 Pelletier et al. *JAMA Network Open.*

Diagnosis	ICD Version	ICD Code	Number of Encounters
Trauma	10	S32058A	13
Trauma	10	S52601C	1
Trauma	10	S72092G	1
Trauma	10	T23001A	9
Trauma	10	S14156A	3
Trauma	10	S06370A	37
Trauma	10	S92344A	1
Trauma	10	S36221A	7
Trauma	10	S52392M	1
Trauma	10	S32811B	5
Trauma	10	S08121A	9
Trauma	10	S40022A	13
Trauma	10	T2103XA	6
Trauma	10	S31813A	5
Trauma	10	S52271C	3
Trauma	10	S42332A	15
Trauma	10	S92314A	1
Trauma	10	S85511A	2
Trauma	10	S52251B	7
Trauma	10	T22321A	7

© 2021 Pelletier et al. *JAMA Network Open.*

Diagnosis	ICD Version	ICD Code	Number of Encounters
Trauma	10	S062X6D	19
Trauma	10	S0231XB	10
Trauma	10	S0184XA	14
Trauma	10	S02671A	11
Trauma	10	S27332A	1
Trauma	10	T22232A	34
Trauma	10	S42014A	13
Trauma	10	T2642XA	1
Trauma	10	T24221A	8
Trauma	10	S72052A	9
Trauma	10	S82402A	19
Trauma	10	S52031K	2
Trauma	10	S8011XA	15
Trauma	10	S27329A	40
Trauma	10	S3130XA	7
Trauma	10	S5782XA	8
Trauma	10	S51802D	3
Trauma	10	T1491	144
Trauma	10	S3660XA	7
Trauma	10	S82872D	2

© 2021 Pelletier et al. *JAMA Network Open.*

Diagnosis	ICD Version	ICD Code	Number of Encounters
Trauma	10	T25221D	2
Trauma	10	S39011A	10
Trauma	10	S42255A	1
Trauma	10	S91309A	2
Trauma	10	S32402A	24
Trauma	10	S42391A	4
Trauma	10	S12600K	1
Trauma	10	S1183XA	10
Trauma	10	S12130A	3
Trauma	10	S61512A	40
Trauma	10	S32030A	15
Trauma	10	S064X9D	20
Trauma	10	S36115D	1
Trauma	10	S24103A	12
Trauma	10	S72412A	5
Trauma	10	S71131D	1
Trauma	10	S14104A	11
Trauma	10	S42392B	3
Trauma	10	S82201K	16
Trauma	10	S143XXA	65

© 2021 Pelletier et al. *JAMA Network Open.*

Diagnosis	ICD Version	ICD Code	Number of Encounters
Trauma	10	S37001A	4
Trauma	10	S069X5A	2
Trauma	10	S85152A	1
Trauma	10	S12112A	8
Trauma	10	S2500XD	1
Trauma	10	T25322A	67
Trauma	10	T25332A	5
Trauma	10	S59022A	7
Trauma	10	S3729XA	46
Trauma	10	S72022A	9
Trauma	10	S72002B	3
Trauma	10	T24231A	34
Trauma	10	S06351A	17
Trauma	10	S36539A	3
Trauma	10	S51821A	8
Trauma	10	S81002A	6
Trauma	10	T148XXA	17
Trauma	10	S82891K	2
Trauma	10	S9304XA	7
Trauma	10	S82851Q	1

© 2021 Pelletier et al. *JAMA Network Open.*

Diagnosis	ICD Version	ICD Code	Number of Encounters
Trauma	10	S2249XA	6
Trauma	10	S0511XA	56
Trauma	10	S72042B	1
Trauma	10	S32432A	7
Trauma	10	S27808A	12
Trauma	10	S82874A	3
Trauma	10	S12300A	31
Trauma	10	S42402B	15
Trauma	10	S82222B	31
Trauma	10	S3339XD	1
Trauma	10	S92414B	2
Trauma	10	S61452A	63
Trauma	10	S79142A	20
Trauma	10	S062X9S	20
Trauma	10	T24232A	37
Trauma	10	T22291A	13
Trauma	10	S36533A	26
Trauma	10	S12490A	21
Trauma	10	S42492B	15
Trauma	10	S89311A	3

© 2021 Pelletier et al. *JAMA Network Open.*

Diagnosis	ICD Version	ICD Code	Number of Encounters
Trauma	10	S62614B	6
Trauma	10	S52221C	1
Trauma	10	S52001B	5
Trauma	10	T25211A	8
Trauma	10	S52515A	1
Trauma	10	S81001A	10
Trauma	10	S81011D	1
Trauma	10	S42475B	2
Trauma	10	S22019B	1
Trauma	10	S148XXA	5
Trauma	10	S41132A	1
Trauma	10	S92102A	8
Trauma	10	S41052A	7
Trauma	10	S49102A	7
Trauma	10	S31634A	6
Trauma	10	S32591A	81
Trauma	10	S11021A	11
Trauma	10	S52202P	4
Trauma	10	T20112A	1
Trauma	10	S68617A	1

© 2021 Pelletier et al. *JAMA Network Open.*

Diagnosis	ICD Version	ICD Code	Number of Encounters
Trauma	10	S42341A	25
Trauma	10	S30862A	3
Trauma	10	S79132A	21
Trauma	10	S68012A	9
Trauma	10	S61031A	1
Trauma	10	S06369A	83
Trauma	10	S42302G	1
Trauma	10	S61022D	1
Trauma	10	S96891A	2
Trauma	10	S60222A	5
Trauma	10	S36220A	10
Trauma	10	S32431A	7
Trauma	10	S39023A	1
Trauma	10	S72322C	6
Trauma	10	S42321B	10
Trauma	10	S3681XA	6
Trauma	10	S68623A	10
Trauma	10	S59202A	12
Trauma	10	S81012D	3
Trauma	10	S59201A	11

© 2021 Pelletier et al. *JAMA Network Open.*

Diagnosis	ICD Version	ICD Code	Number of Encounters
Trauma	10	T3120	7
Trauma	10	S52312A	2
Trauma	10	T3130	2
Trauma	10	T2005XD	1
Trauma	10	T2121XD	2
Trauma	10	S12591A	4
Trauma	10	T2024XA	16
Trauma	10	T23242A	12
Trauma	10	S62663B	1
Trauma	10	S81041A	17
Trauma	10	T17290A	10
Trauma	10	S8012XA	22
Trauma	10	T310	33
Trauma	10	T23202A	65
Trauma	10	S8261XA	26
Trauma	10	S81802A	29
Trauma	10	S31631A	6
Trauma	10	S80211A	10
Trauma	10	S82841B	16
Trauma	10	S82462A	1

© 2021 Pelletier et al. *JAMA Network Open.*

Diagnosis	ICD Version	ICD Code	Number of Encounters
Trauma	10	S82001B	8
Trauma	10	S42491S	1
Trauma	10	T23231A	13
Trauma	10	T22212A	86
Trauma	10	S1093XA	24
Trauma	10	S0291XS	4
Trauma	10	T23261A	29
Trauma	10	T3110	23
Trauma	10	T23041A	1
Trauma	10	S89031A	11
Trauma	10	T17808A	9
Trauma	10	S12590A	19
Trauma	10	T2025XA	35
Trauma	10	S3135XA	3
Trauma	10	S31001A	1
Trauma	10	S06381A	1
Trauma	10	T22292A	23
Trauma	10	S82221B	28
Trauma	10	S06370S	1
Trauma	10	S36119A	5

© 2021 Pelletier et al. *JAMA Network Open.*

Diagnosis	ICD Version	ICD Code	Number of Encounters
Trauma	10	T2035XA	26
Trauma	10	S71121A	6
Trauma	10	S42441B	6
Trauma	10	S71131A	18
Trauma	10	S72434B	1
Trauma	10	S62231B	1
Trauma	10	S31104A	10
Trauma	10	S51801A	4
Trauma	10	T23252A	57
Trauma	10	S14102A	9
Trauma	10	S329XXA	9
Trauma	10	T25221A	59
Trauma	10	S36021A	6
Trauma	10	S061X0A	15
Trauma	10	T17508A	28
Trauma	10	S0104XA	8
Trauma	10	S22078A	7
Trauma	10	T2123XA	48
Trauma	10	S51832A	5
Trauma	10	T23292A	23

© 2021 Pelletier et al. *JAMA Network Open.*

Diagnosis	ICD Version	ICD Code	Number of Encounters
Trauma	10	S4401XA	3
Trauma	10	T2127XA	33
Trauma	10	S61531A	1
Trauma	10	T3111	11
Trauma	10	T2122XS	1
Trauma	10	S3142XA	4
Trauma	10	S82152B	4
Trauma	10	S0240FB	4
Trauma	10	S12111A	8
Trauma	10	S3639XA	12
Trauma	10	S82102B	8
Trauma	10	S41102A	8
Trauma	10	S62391A	2
Trauma	10	T22222A	10
Trauma	10	S3121XA	21
Trauma	10	T184XXD	1
Trauma	10	S08112A	3
Trauma	10	S81801A	28
Trauma	10	S14102D	1
Trauma	10	S82245A	9

© 2021 Pelletier et al. *JAMA Network Open.*

Diagnosis	ICD Version	ICD Code	Number of Encounters
Trauma	10	S91312A	30
Trauma	10	S12691A	6
Trauma	10	S3219XB	1
Trauma	10	S02621A	30
Trauma	10	S9305XA	3
Trauma	10	S52201C	1
Trauma	10	S31000A	3
Trauma	10	S061X0S	2
Trauma	10	S02102A	10
Trauma	10	S72422B	4
Trauma	10	S8252XB	14
Trauma	10	S21342A	14
Trauma	10	S21349A	2
Trauma	10	S06367A	9
Trauma	10	T2169XA	1
Trauma	10	S6701XA	4
Trauma	10	T24712A	2
Trauma	10	S34139A	3
Trauma	10	S62302B	3
Trauma	10	T25212A	12

© 2021 Pelletier et al. *JAMA Network Open.*

Diagnosis	ICD Version	ICD Code	Number of Encounters
Trauma	10	S1214XA	2
Trauma	10	S72352C	11
Trauma	10	T24202A	42
Trauma	10	S92222B	2
Trauma	10	T2053XA	1
Trauma	10	S22081A	20
Trauma	10	S92421A	9
Trauma	10	S49101A	5
Trauma	10	T23302A	40
Trauma	10	T23362A	19
Trauma	10	T22252A	33
Trauma	10	S13170A	4
Trauma	10	S98322A	15
Trauma	10	T24292A	12
Trauma	10	T22331A	37
Trauma	10	T17390A	14
Trauma	10	S91302A	27
Trauma	10	T23341A	3
Trauma	10	S92411B	16
Trauma	10	S14115A	6

© 2021 Pelletier et al. *JAMA Network Open.*

Diagnosis	ICD Version	ICD Code	Number of Encounters
Trauma	10	S92331B	9
Trauma	10	S24114D	2
Trauma	10	S01312A	24
Trauma	10	S42015A	14
Trauma	10	S82243A	1
Trauma	10	S49001A	4
Trauma	10	S82841C	6
Trauma	10	S92041A	1
Trauma	10	S88112A	4
Trauma	10	S98141A	18
Trauma	10	S332XXA	6
Trauma	10	S82291B	20
Trauma	10	S12041A	3
Trauma	10	S67196A	5
Trauma	10	T2135XA	44
Trauma	10	S3422XA	2
Trauma	10	S91111A	5
Trauma	10	S32119A	13
Trauma	10	S32412A	4
Trauma	10	S02118B	4

© 2021 Pelletier et al. *JAMA Network Open.*

Diagnosis	ICD Version	ICD Code	Number of Encounters
Trauma	10	S6402XA	6
Trauma	10	T1502XA	5
Trauma	10	S064X6A	4
Trauma	10	S30810A	15
Trauma	10	S32031A	25
Trauma	10	S82115S	1
Trauma	10	S79129A	3
Trauma	10	S066X9S	13
Trauma	10	S58111A	2
Trauma	10	S82311A	4
Trauma	10	S06366A	2
Trauma	10	S31130A	4
Trauma	10	S8251XB	15
Trauma	10	S42352B	12
Trauma	10	S0291XB	16
Trauma	10	S01121A	9
Trauma	10	S0183XA	7
Trauma	10	S5402XA	8
Trauma	10	S68112A	4
Trauma	10	T22392A	17

© 2021 Pelletier et al. *JAMA Network Open.*

Diagnosis	ICD Version	ICD Code	Number of Encounters
Trauma	10	S41101S	1
Trauma	10	S42011A	5
Trauma	10	S32110A	8
Trauma	10	T276XXA	2
Trauma	10	S72302E	1
Trauma	10	S72034A	8
Trauma	10	S3022XA	26
Trauma	10	S42301B	10
Trauma	10	S36590A	7
Trauma	10	S72002K	8
Trauma	10	S24114A	10
Trauma	10	S00512A	8
Trauma	10	S92412A	5
Trauma	10	S42012A	8
Trauma	10	S37092A	7
Trauma	10	S7402XA	2
Trauma	10	S98131A	9
Trauma	10	S82862A	1
Trauma	10	S06336A	1
Trauma	10	S12301A	6

© 2021 Pelletier et al. *JAMA Network Open.*

Diagnosis	ICD Version	ICD Code	Number of Encounters
Trauma	10	S32302B	2
Trauma	10	S79011K	2
Trauma	10	S32028A	26
Trauma	10	S12150A	1
Trauma	10	S42111A	2
Trauma	10	S83422A	3
Trauma	10	S72042K	1
Trauma	10	S91142A	4
Trauma	10	S62334A	2
Trauma	10	S82302C	9
Trauma	10	S46222A	5
Trauma	10	S36292A	2
Trauma	10	S51852S	1
Trauma	10	S31109A	9
Trauma	10	S32020A	26
Trauma	10	S82452A	14
Trauma	10	S42451B	5
Trauma	10	S31639A	2
Trauma	10	S92314B	1
Trauma	10	S82042B	12

© 2021 Pelletier et al. *JAMA Network Open.*

Diagnosis	ICD Version	ICD Code	Number of Encounters
Trauma	10	S42445B	2
Trauma	10	S81851D	2
Trauma	10	S31600A	2
Trauma	10	S52692C	1
Trauma	10	S51021A	8
Trauma	10	S96121A	8
Trauma	10	S7222XB	5
Trauma	10	S92002A	11
Trauma	10	S32059A	20
Trauma	10	S91351A	7
Trauma	10	S61401D	1
Trauma	10	S56292D	1
Trauma	10	S62392B	2
Trauma	10	S3421XA	5
Trauma	10	S42201B	7
Trauma	10	S72451B	2
Trauma	10	S92311B	20
Trauma	10	S82101B	5
Trauma	10	S31134A	10
Trauma	10	S42391B	3

© 2021 Pelletier et al. *JAMA Network Open.*

Diagnosis	ICD Version	ICD Code	Number of Encounters
Trauma	10	S82034A	1
Trauma	10	S82209A	5
Trauma	10	T24392A	18
Trauma	10	S8255XA	6
Trauma	10	S149XXA	3
Trauma	10	T23332A	9
Trauma	10	S72491B	10
Trauma	10	S12391A	3
Trauma	10	T2022XA	9
Trauma	10	S40861A	4
Trauma	10	S52532A	5
Trauma	10	S20219A	17
Trauma	10	S42402K	2
Trauma	10	S92322A	8
Trauma	10	S271XXA	38
Trauma	10	S36509A	5
Trauma	10	T1501XA	7
Trauma	10	S37062D	1
Trauma	10	S52351A	12
Trauma	10	S49022A	14

© 2021 Pelletier et al. *JAMA Network Open.*

Diagnosis	ICD Version	ICD Code	Number of Encounters
Trauma	10	S92001B	13
Trauma	10	S2239XA	2
Trauma	10	S0240EA	36
Trauma	10	S3994XA	3
Trauma	10	S53401A	1
Trauma	10	S22061A	13
Trauma	10	S92402A	2
Trauma	10	S72335B	1
Trauma	10	S50312A	6
Trauma	10	S80812A	7
Trauma	10	S82041B	7
Trauma	10	S02122A	7
Trauma	10	S71101A	21
Trauma	10	S02832A	7
Trauma	10	S42322A	29
Trauma	10	S0551XA	16
Trauma	10	S32422D	2
Trauma	10	S31100A	2
Trauma	10	S82401D	1
Trauma	10	T22352A	25

© 2021 Pelletier et al. *JAMA Network Open.*

Diagnosis	ICD Version	ICD Code	Number of Encounters
Trauma	10	S728X2A	25
Trauma	10	S51852A	25
Trauma	10	S55112A	4
Trauma	10	S36409A	11
Trauma	10	S62622B	3
Trauma	10	S72309A	9
Trauma	10	S20351A	2
Trauma	10	S12200A	18
Trauma	10	S31814A	5
Trauma	10	S43221A	1
Trauma	10	S21241A	4
Trauma	10	T2019XA	4
Trauma	10	S32051A	27
Trauma	10	S3669XA	11
Trauma	10	S5292XA	22
Trauma	10	S91331A	19
Trauma	10	S68123A	10
Trauma	10	S12100K	1
Trauma	10	S30842A	4
Trauma	10	S42001K	1

© 2021 Pelletier et al. *JAMA Network Open.*

Diagnosis	ICD Version	ICD Code	Number of Encounters
Trauma	10	S02651A	48
Trauma	10	S43222A	1
Trauma	10	T23331A	22
Trauma	10	S12120A	9
Trauma	10	S24102A	10
Trauma	10	T17328A	6
Trauma	10	T23079A	1
Trauma	10	S72302B	15
Trauma	10	S13121A	18
Trauma	10	S01101A	1
Trauma	10	S0086XA	8
Trauma	10	S7711XA	4
Trauma	10	S2220XA	26
Trauma	10	T22341A	3
Trauma	10	S81852A	26
Trauma	10	S129XXA	5
Trauma	10	S42102A	7
Trauma	10	S0285XB	3
Trauma	10	S68521A	7
Trauma	10	S89322A	14

© 2021 Pelletier et al. *JAMA Network Open.*

Diagnosis	ICD Version	ICD Code	Number of Encounters
Trauma	10	T2036XA	21
Trauma	10	S92401B	2
Trauma	10	S98212A	3
Trauma	10	T23062A	2
Trauma	10	T24001A	7
Trauma	10	T22221A	10
Trauma	10	S32443K	1
Trauma	10	S51051A	5
Trauma	10	T20311A	5
Trauma	10	T23301A	21
Trauma	10	T23039A	1
Trauma	10	S79121S	1
Trauma	10	S42291G	1
Trauma	10	T1511XA	4
Trauma	10	S9781XS	1
Trauma	10	S36209A	8
Trauma	10	S61511A	19
Trauma	10	S32028D	1
Trauma	10	S12000A	25
Trauma	10	S36599A	1

© 2021 Pelletier et al. *JAMA Network Open.*

Diagnosis	ICD Version	ICD Code	Number of Encounters
Trauma	10	S92492A	1
Trauma	10	S02402B	6
Trauma	10	T23032A	3
Trauma	10	T25221S	1
Trauma	10	S329XXB	3
Trauma	10	S98142A	8
Trauma	10	S91302D	8
Trauma	10	S82851C	3
Trauma	10	S85091A	4
Trauma	10	T22361A	2
Trauma	10	S7292XB	1
Trauma	10	S92111B	4
Trauma	10	T22359A	1
Trauma	10	S72402C	5
Trauma	10	S7292XF	1
Trauma	10	T23022A	2
Trauma	10	S82151C	1
Trauma	10	S92022A	1
Trauma	10	T24322A	11
Trauma	10	S70362A	4

© 2021 Pelletier et al. *JAMA Network Open.*

Diagnosis	ICD Version	ICD Code	Number of Encounters
Trauma	10	T2029XD	1
Trauma	10	T2020XD	5
Trauma	10	S96022A	4
Trauma	10	S32511B	1
Trauma	10	S13171A	3
Trauma	10	S42413B	4
Trauma	10	S21212A	6
Trauma	10	T23002A	8
Trauma	10	S36518A	1
Trauma	10	T22092A	4
Trauma	10	T2107XA	4
Trauma	10	S7290XA	9
Trauma	10	S52001A	2
Trauma	10	T24131A	2
Trauma	10	S52334A	2
Trauma	10	T22219A	1
Trauma	10	S37069A	3
Trauma	10	S32121A	4
Trauma	10	S06890A	57
Trauma	10	S2191XA	1

© 2021 Pelletier et al. *JAMA Network Open.*

Diagnosis	ICD Version	ICD Code	Number of Encounters
Trauma	10	S028XXB	7
Trauma	10	T24201A	27
Trauma	10	T24291A	11
Trauma	10	S91301A	29
Trauma	10	S31811A	12
Trauma	10	S42421B	8
Trauma	10	S06389A	6
Trauma	10	S0081XD	1
Trauma	10	S82232C	3
Trauma	10	S82292H	1
Trauma	10	S82202Q	2
Trauma	10	S82202N	6
Trauma	10	T2016XA	1
Trauma	10	S00411A	1
Trauma	10	S52292B	10
Trauma	10	S82491A	6
Trauma	10	S0451XA	10
Trauma	10	S0105XD	2
Trauma	10	S062X8A	4
Trauma	10	T23209A	2

© 2021 Pelletier et al. *JAMA Network Open.*

Diagnosis	ICD Version	ICD Code	Number of Encounters
Trauma	10	T22329A	1
Trauma	10	S2221XA	12
Trauma	10	S0180XA	13
Trauma	10	S06301A	24
Trauma	10	T23331D	1
Trauma	10	S62522B	7
Trauma	10	S51002A	3
Trauma	10	S30861A	6
Trauma	10	T24302A	31
Trauma	10	S92021B	1
Trauma	10	S42402D	1
Trauma	10	S61051A	8
Trauma	10	S71002A	5
Trauma	10	S01321A	1
Trauma	10	S0211BA	2
Trauma	10	S42001B	1
Trauma	10	S0281XB	16
Trauma	10	S62131B	2
Trauma	10	S45112A	7
Trauma	10	S31609A	4

© 2021 Pelletier et al. *JAMA Network Open.*

Diagnosis	ICD Version	ICD Code	Number of Encounters
Trauma	10	S71122A	8
Trauma	10	S62613B	7
Trauma	10	T24222A	12
Trauma	10	S15311A	2
Trauma	10	S36408A	7
Trauma	10	S64496A	2
Trauma	10	S32441A	14
Trauma	10	S065X8A	4
Trauma	10	S42192A	2
Trauma	10	S31809A	4
Trauma	10	S36530A	25
Trauma	10	T22019A	1
Trauma	10	S13100A	2
Trauma	10	S15321A	3
Trauma	10	S0280XA	16
Trauma	10	S40811A	2
Trauma	10	S82109A	2
Trauma	10	S72302K	5
Trauma	10	S92332B	2
Trauma	10	T24321A	9

© 2021 Pelletier et al. *JAMA Network Open.*

Diagnosis	ICD Version	ICD Code	Number of Encounters
Trauma	10	S41022A	1
Trauma	10	S82401A	17
Trauma	10	S72492C	6
Trauma	10	T23221A	3
Trauma	10	S46821A	4
Trauma	10	S20311A	5
Trauma	10	S83112A	1
Trauma	10	S72332B	9
Trauma	10	T23321A	6
Trauma	10	S75092A	4
Trauma	10	S21151A	3
Trauma	10	S8252XC	3
Trauma	10	S55101A	1
Trauma	10	S92141B	2
Trauma	10	S24109A	9
Trauma	10	S32501A	8
Trauma	10	S31821S	1
Trauma	10	S06355A	2
Trauma	10	S40852A	2
Trauma	10	S42434A	1

© 2021 Pelletier et al. *JAMA Network Open.*

Diagnosis	ICD Version	ICD Code	Number of Encounters
Trauma	10	S01311A	19
Trauma	10	S145XXA	1
Trauma	10	S01542A	5
Trauma	10	T24301A	46
Trauma	10	S43005A	2
Trauma	10	S3661XA	1
Trauma	10	T17410A	1
Trauma	10	S82261C	3
Trauma	10	S31149A	7
Trauma	10	S1184XA	16
Trauma	10	T2012XA	2
Trauma	10	S51001A	3
Trauma	10	S98321A	16
Trauma	10	S2619XA	8
Trauma	10	S61522A	4
Trauma	10	S42401B	11
Trauma	10	S3289XD	3
Trauma	10	S82252C	25
Trauma	10	T22322A	8
Trauma	10	S61541A	1

© 2021 Pelletier et al. *JAMA Network Open.*

Diagnosis	ICD Version	ICD Code	Number of Encounters
Trauma	10	S22000A	3
Trauma	10	S65011A	7
Trauma	10	S01402A	1
Trauma	10	S68622A	5
Trauma	10	S3600XA	4
Trauma	10	T25122A	1
Trauma	10	T20312A	5
Trauma	10	S1985XA	2
Trauma	10	S31010A	8
Trauma	10	S68116A	10
Trauma	10	S3133XA	3
Trauma	10	S85001A	1
Trauma	10	S90511A	1
Trauma	10	S0285XA	12
Trauma	10	S62336A	6
Trauma	10	S62607B	1
Trauma	10	S85092A	6
Trauma	10	S12090A	15
Trauma	10	S42251A	3
Trauma	10	S66328A	1

© 2021 Pelletier et al. *JAMA Network Open.*

Diagnosis	ICD Version	ICD Code	Number of Encounters
Trauma	10	S40021A	4
Trauma	10	S728X2G	4
Trauma	10	S72492B	8
Trauma	10	S36113D	3
Trauma	10	S72052K	1
Trauma	10	S49011A	5
Trauma	10	S76812A	3
Trauma	10	S00212A	4
Trauma	10	S7010XA	2
Trauma	10	S52332B	10
Trauma	10	S36591A	6
Trauma	10	S49021S	1
Trauma	10	S1190XA	4
Trauma	10	S75912A	1
Trauma	10	S79812A	3
Trauma	10	S36239A	16
Trauma	10	S22079B	1
Trauma	10	T191XXA	6
Trauma	10	S31111A	5
Trauma	10	S68121A	19

© 2021 Pelletier et al. *JAMA Network Open.*

Diagnosis	ICD Version	ICD Code	Number of Encounters
Trauma	10	S82044B	1
Trauma	10	S82424A	1
Trauma	10	S70211A	2
Trauma	10	S92142A	5
Trauma	10	S52255B	1
Trauma	10	S89011A	8
Trauma	10	S32464A	1
Trauma	10	S82872B	3
Trauma	10	S81822A	11
Trauma	10	S83015A	6
Trauma	10	S52032B	1
Trauma	10	S82091A	21
Trauma	10	S96821A	6
Trauma	10	S72062A	7
Trauma	10	S14125A	4
Trauma	10	S36299A	7
Trauma	10	S51012A	15
Trauma	10	S00211A	6
Trauma	10	S92334B	3
Trauma	10	S21311A	5

© 2021 Pelletier et al. *JAMA Network Open.*

Diagnosis	ICD Version	ICD Code	Number of Encounters
Trauma	10	S32452A	16
Trauma	10	S49132A	7
Trauma	10	S80872A	2
Trauma	10	S62615B	6
Trauma	10	S80252A	3
Trauma	10	S61311A	3
Trauma	10	S97112A	9
Trauma	10	S72341G	2
Trauma	10	S5291XD	1
Trauma	10	S82292B	19
Trauma	10	S82292E	1
Trauma	10	S82092C	1
Trauma	10	S75011A	1
Trauma	10	T24299A	1
Trauma	10	S82032A	11
Trauma	10	S91332A	19
Trauma	10	S73041A	1
Trauma	10	S66822A	8
Trauma	10	S82132A	4
Trauma	10	S79191A	22

© 2021 Pelletier et al. *JAMA Network Open.*

Diagnosis	ICD Version	ICD Code	Number of Encounters
Trauma	10	S62631A	3
Trauma	10	S42432A	26
Trauma	10	S3710XA	3
Trauma	10	T22391A	12
Trauma	10	S064X4A	2
Trauma	10	S62324B	3
Trauma	10	S82202K	7
Trauma	10	S0191XA	3
Trauma	10	T1581XA	5
Trauma	10	S3289XA	5
Trauma	10	S61241A	3
Trauma	10	S90859A	1
Trauma	10	S8265XB	2
Trauma	10	S63592A	1
Trauma	10	S72092K	3
Trauma	10	S062X4A	4
Trauma	10	T3144	2
Trauma	10	S62606B	1
Trauma	10	S0005XA	1
Trauma	10	T20211A	9

© 2021 Pelletier et al. *JAMA Network Open.*

Diagnosis	ICD Version	ICD Code	Number of Encounters
Trauma	10	S02119G	3
Trauma	10	S62664B	2
Trauma	10	T18100A	2
Trauma	10	S36259A	3
Trauma	10	S3692XA	4
Trauma	10	S73003A	3
Trauma	10	S2232XA	41
Trauma	10	S32512B	2
Trauma	10	S92001A	10
Trauma	10	S41131A	3
Trauma	10	S72309D	1
Trauma	10	S62393B	1
Trauma	10	S46991A	1
Trauma	10	S73006A	4
Trauma	10	S43224A	25
Trauma	10	S49001P	2
Trauma	10	S06347A	9
Trauma	10	S2242XD	2
Trauma	10	S82292K	1
Trauma	10	S36116S	1

© 2021 Pelletier et al. *JAMA Network Open.*

Diagnosis	ICD Version	ICD Code	Number of Encounters
Trauma	10	S0211GB	10
Trauma	10	S3992XA	26
Trauma	10	S0502XA	27
Trauma	10	S72355A	9
Trauma	10	S30871A	1
Trauma	10	T2131XD	4
Trauma	10	S52331B	8
Trauma	10	S32000G	1
Trauma	10	S1989XS	1
Trauma	10	S72441A	18
Trauma	10	S22009A	6
Trauma	10	S3141XD	4
Trauma	10	S62612B	8
Trauma	10	T300	5
Trauma	10	S0267XA	6
Trauma	10	S065X2A	11
Trauma	10	S52134A	1
Trauma	10	S3732XA	3
Trauma	10	T2105XD	2
Trauma	10	S42331A	14

© 2021 Pelletier et al. *JAMA Network Open.*

Diagnosis	ICD Version	ICD Code	Number of Encounters
Trauma	10	S61253A	5
Trauma	10	S40211A	8
Trauma	10	S31829A	4
Trauma	10	S42252A	4
Trauma	10	S61412A	13
Trauma	10	S3093XA	1
Trauma	10	S06364A	2
Trauma	10	T25091A	4
Trauma	10	S61551A	7
Trauma	10	S72452H	1
Trauma	10	S83242A	5
Trauma	10	S37021A	3
Trauma	10	S90822A	3
Trauma	10	T23309A	1
Trauma	10	S42391G	1
Trauma	10	S59291A	4
Trauma	10	T2122XD	3
Trauma	10	S32009A	7
Trauma	10	S22008A	3
Trauma	10	S020XXK	6

© 2021 Pelletier et al. *JAMA Network Open.*

Diagnosis	ICD Version	ICD Code	Number of Encounters
Trauma	10	S00262A	7
Trauma	10	S82231B	26
Trauma	10	S0291XG	4
Trauma	10	S52232A	8
Trauma	10	S52041A	4
Trauma	10	S14136A	1
Trauma	10	T198XXA	1
Trauma	10	T17310A	2
Trauma	10	T22091A	2
Trauma	10	S82235A	6
Trauma	10	S92191B	2
Trauma	10	S52035A	1
Trauma	10	S76122A	12
Trauma	10	S72021A	10
Trauma	10	S064X3A	5
Trauma	10	S82134A	2
Trauma	10	S37022S	1
Trauma	10	S32451A	5
Trauma	10	S86812A	6
Trauma	10	T25092D	1

© 2021 Pelletier et al. *JAMA Network Open.*

Diagnosis	ICD Version	ICD Code	Number of Encounters
Trauma	10	S069X6A	3
Trauma	10	S22071A	5
Trauma	10	S1980XA	9
Trauma	10	S49141A	2
Trauma	10	S31011A	1
Trauma	10	S51052A	6
Trauma	10	S42309A	1
Trauma	10	S31613A	7
Trauma	10	S92031A	2
Trauma	10	S98112A	20
Trauma	10	T2003XA	4
Trauma	10	S81819A	1
Trauma	10	S92491B	1
Trauma	10	S36262A	8
Trauma	10	S5290XA	4
Trauma	10	S33121A	1
Trauma	10	T25232A	7
Trauma	10	T25022A	12
Trauma	10	T2010XA	6
Trauma	10	S80811A	5

© 2021 Pelletier et al. *JAMA Network Open.*

Diagnosis	ICD Version	ICD Code	Number of Encounters
Trauma	10	S92253A	2
Trauma	10	S02841A	7
Trauma	10	S82153A	4
Trauma	10	S15091A	5
Trauma	10	S98211A	7
Trauma	10	S04012A	1
Trauma	10	S15392A	1
Trauma	10	S42302D	3
Trauma	10	S91032A	3
Trauma	10	S52235A	2
Trauma	10	S061X6A	8
Trauma	10	S78122D	1
Trauma	10	S14103D	1
Trauma	10	S21339A	1
Trauma	10	S92422A	6
Trauma	10	S79112A	16
Trauma	10	S33141A	2
Trauma	10	S31112A	5
Trauma	10	S82202D	5
Trauma	10	S0101XD	3

© 2021 Pelletier et al. *JAMA Network Open.*

Diagnosis	ICD Version	ICD Code	Number of Encounters
Trauma	10	S020XXD	55
Trauma	10	S42472B	2
Trauma	10	S92252K	2
Trauma	10	S0301XA	2
Trauma	10	S7291XD	10
Trauma	10	S01432A	5
Trauma	10	S93129A	1
Trauma	10	S92414A	1
Trauma	10	S92212B	2
Trauma	10	S1180XA	3
Trauma	10	S31115A	1
Trauma	10	S064X0D	15
Trauma	10	T07	1
Trauma	10	S7292XD	12
Trauma	10	S71141A	16
Trauma	10	S82221C	4
Trauma	10	S02641A	19
Trauma	10	S82291E	1
Trauma	10	S82201E	4
Trauma	10	S8491XA	1

© 2021 Pelletier et al. *JAMA Network Open.*

Diagnosis	ICD Version	ICD Code	Number of Encounters
Trauma	10	S96021A	2
Trauma	10	S8411XA	8
Trauma	10	S71119A	1
Trauma	10	S14154D	6
Trauma	10	S32810D	11
Trauma	10	S50811A	7
Trauma	10	S12190A	20
Trauma	10	S0093XA	18
Trauma	10	S89101A	11
Trauma	10	S83001A	5
Trauma	10	S12501A	12
Trauma	10	T2104XS	2
Trauma	10	T2134XS	1
Trauma	10	S91011A	15
Trauma	10	S61210D	2
Trauma	10	S68610D	1
Trauma	10	S20229A	5
Trauma	10	S42335A	3
Trauma	10	S3982XA	8
Trauma	10	S42322B	17

© 2021 Pelletier et al. *JAMA Network Open.*

Diagnosis	ICD Version	ICD Code	Number of Encounters
Trauma	10	S06890D	13
Trauma	10	S06340D	9
Trauma	10	S62643A	1
Trauma	10	S61432A	4
Trauma	10	S83201A	2
Trauma	10	S62317A	4
Trauma	10	S61259A	1
Trauma	10	S82121A	5
Trauma	10	S51851A	21
Trauma	10	S3094XA	1
Trauma	10	S32021D	3
Trauma	10	S51852D	4
Trauma	10	S9781XA	19
Trauma	10	S59012A	2
Trauma	10	S52541B	1
Trauma	10	S0592XA	3
Trauma	10	S51842A	6
Trauma	10	S91301D	4
Trauma	10	S31142A	1
Trauma	10	T272XXA	3

© 2021 Pelletier et al. *JAMA Network Open.*

Diagnosis	ICD Version	ICD Code	Number of Encounters
Trauma	10	S82202F	1
Trauma	10	S3681XD	1
Trauma	10	S53004A	2
Trauma	10	S91021A	4
Trauma	10	S52262B	1
Trauma	10	S61452D	3
Trauma	10	S61251A	12
Trauma	10	S52691B	8
Trauma	10	S98132A	4
Trauma	10	S02411K	3
Trauma	10	S61052A	4
Trauma	10	S72491P	2
Trauma	10	S52309B	1
Trauma	10	S86121A	7
Trauma	10	S60462A	2
Trauma	10	S9031XA	4
Trauma	10	S62630A	6
Trauma	10	S83114A	2
Trauma	10	S31109D	2
Trauma	10	S52091B	3

© 2021 Pelletier et al. *JAMA Network Open.*

Diagnosis	ICD Version	ICD Code	Number of Encounters
Trauma	10	S62615A	4
Trauma	10	S9782XA	17
Trauma	10	S46321A	3
Trauma	10	S060X9D	9
Trauma	10	S72322D	2
Trauma	10	S32040A	5
Trauma	10	S37818A	5
Trauma	10	S82144A	5
Trauma	10	S6412XA	5
Trauma	10	S1182XA	4
Trauma	10	S7292XP	2
Trauma	10	S20221A	2
Trauma	10	S52292A	5
Trauma	10	S8391XA	4
Trauma	10	S79822A	2
Trauma	10	S14126A	4
Trauma	10	S64494A	3
Trauma	10	S82841D	1
Trauma	10	S48112A	3
Trauma	10	S0992XD	2

© 2021 Pelletier et al. *JAMA Network Open.*

Diagnosis	ICD Version	ICD Code	Number of Encounters
Trauma	10	S82843A	1
Trauma	10	S92352A	5
Trauma	10	S7225XA	15
Trauma	10	S82312A	4
Trauma	10	S34113A	5
Trauma	10	S5420XA	1
Trauma	10	S88122A	3
Trauma	10	S08121D	2
Trauma	10	S62646B	1
Trauma	10	S68421A	3
Trauma	10	S065X0S	18
Trauma	10	S72323A	1
Trauma	10	S11021S	1
Trauma	10	S76011A	5
Trauma	10	S32444A	2
Trauma	10	S41121A	4
Trauma	10	S071XXA	8
Trauma	10	S90112A	1
Trauma	10	S11023A	2
Trauma	10	S68021A	14

© 2021 Pelletier et al. *JAMA Network Open.*

Diagnosis	ICD Version	ICD Code	Number of Encounters
Trauma	10	S68117A	5
Trauma	10	S0282XD	2
Trauma	10	S52022B	8
Trauma	10	S72145A	12
Trauma	10	S68411A	4
Trauma	10	S31110A	7
Trauma	10	S0422XA	1
Trauma	10	S65511A	1
Trauma	10	S08812A	8
Trauma	10	S0292XA	7
Trauma	10	S168XXA	2
Trauma	10	S22082A	10
Trauma	10	S22081D	2
Trauma	10	S92062A	5
Trauma	10	S52552B	3
Trauma	10	S82401G	1
Trauma	10	S32312A	6
Trauma	10	S92522B	2
Trauma	10	S72092D	1
Trauma	10	S82832G	1

© 2021 Pelletier et al. *JAMA Network Open.*

Diagnosis	ICD Version	ICD Code	Number of Encounters
Trauma	10	S060X0D	6
Trauma	10	S062X2D	3
Trauma	10	S02621S	1
Trauma	10	S43102A	1
Trauma	10	S52612B	4
Trauma	10	S24104A	8
Trauma	10	S24113D	8
Trauma	10	S02632A	2
Trauma	10	S76922A	1
Trauma	10	S72352D	7
Trauma	10	S82202M	2
Trauma	10	S32691A	3
Trauma	10	S76101A	1
Trauma	10	S31141A	1
Trauma	10	S62327B	3
Trauma	10	S36520A	9
Trauma	10	S3401XA	4
Trauma	10	S90512A	5
Trauma	10	S83001S	1
Trauma	10	S59232A	1

© 2021 Pelletier et al. *JAMA Network Open.*

Diagnosis	ICD Version	ICD Code	Number of Encounters
Trauma	10	S72452C	3
Trauma	10	S72302P	3
Trauma	10	S34101A	10
Trauma	10	S92122B	2
Trauma	10	S61212D	1
Trauma	10	S55011A	10
Trauma	10	S76191A	6
Trauma	10	S83002A	2
Trauma	10	S86391D	1
Trauma	10	S72001B	6
Trauma	10	S83012D	1
Trauma	10	S06359D	8
Trauma	10	S52362A	4
Trauma	10	S060X1D	8
Trauma	10	S82145A	5
Trauma	10	S82891D	2
Trauma	10	S31133A	6
Trauma	10	S92352B	6
Trauma	10	S66126A	2
Trauma	10	S51812D	2

© 2021 Pelletier et al. *JAMA Network Open.*

Diagnosis	ICD Version	ICD Code	Number of Encounters
Trauma	10	S92351K	1
Trauma	10	S72301D	8
Trauma	10	S8982XA	6
Trauma	10	S72324G	1
Trauma	10	S06356A	2
Trauma	10	S55111A	6
Trauma	10	S86112A	2
Trauma	10	S52611A	2
Trauma	10	S72322B	13
Trauma	10	S82142R	1
Trauma	10	S36893A	24
Trauma	10	S82192B	8
Trauma	10	S60211A	2
Trauma	10	S61213A	2
Trauma	10	S82392K	1
Trauma	10	S32512D	3
Trauma	10	S32029D	5
Trauma	10	S3733XA	14
Trauma	10	S34109D	1
Trauma	10	S21202A	2

© 2021 Pelletier et al. *JAMA Network Open.*

Diagnosis	ICD Version	ICD Code	Number of Encounters
Trauma	10	S24154D	5
Trauma	10	S92312B	10
Trauma	10	S42452B	4
Trauma	10	S065X1D	11
Trauma	10	S8781XA	14
Trauma	10	S91022A	4
Trauma	10	S91002D	1
Trauma	10	S33131A	2
Trauma	10	S0181XD	5
Trauma	10	S82291S	1
Trauma	10	S81021S	1
Trauma	10	S73003D	1
Trauma	10	S11011A	3
Trauma	10	S61441A	7
Trauma	10	S73002D	8
Trauma	10	S0093XD	1
Trauma	10	S81019A	1
Trauma	10	S36122A	1
Trauma	10	T17908S	2
Trauma	10	S71101D	1

© 2021 Pelletier et al. *JAMA Network Open.*

Diagnosis	ICD Version	ICD Code	Number of Encounters
Trauma	10	S5421XA	2
Trauma	10	S31621A	3
Trauma	10	S32491B	2
Trauma	10	S72331C	2
Trauma	10	S82202G	4
Trauma	10	S0451XD	3
Trauma	10	S70311A	6
Trauma	10	S89102A	10
Trauma	10	S48011A	1
Trauma	10	S058X1A	9
Trauma	10	S85141A	2
Trauma	10	S51011A	15
Trauma	10	S52002A	4
Trauma	10	S143XXD	9
Trauma	10	S32519A	2
Trauma	10	S61211A	7
Trauma	10	S30814A	6
Trauma	10	S79011G	6
Trauma	10	S0889XS	1
Trauma	10	S0889XD	1

© 2021 Pelletier et al. *JAMA Network Open.*

Diagnosis	ICD Version	ICD Code	Number of Encounters
Trauma	10	S92911B	6
Trauma	10	S22039A	8
Trauma	10	S82002C	1
Trauma	10	S0452XA	10
Trauma	10	S41051A	4
Trauma	10	S37813A	3
Trauma	10	S27399A	4
Trauma	10	S14142A	4
Trauma	10	S08112D	1
Trauma	10	S7401XD	3
Trauma	10	S72145D	1
Trauma	10	S21142A	8
Trauma	10	S72402G	2
Trauma	10	S82162A	2
Trauma	10	S32411A	2
Trauma	10	S01142A	3
Trauma	10	S91031A	2
Trauma	10	S0993XS	1
Trauma	10	S31020A	2
Trauma	10	S49192A	6

© 2021 Pelletier et al. *JAMA Network Open.*

Diagnosis	ICD Version	ICD Code	Number of Encounters
Trauma	10	S78112A	1
Trauma	10	S49112A	3
Trauma	10	S0281XD	1
Trauma	10	S42101A	6
Trauma	10	S45102A	2
Trauma	10	S92061A	5
Trauma	10	S72435A	1
Trauma	10	S51822D	1
Trauma	10	S82101K	2
Trauma	10	S62322B	4
Trauma	10	S62024K	1
Trauma	10	S0552XA	10
Trauma	10	S43004A	3
Trauma	10	S67193A	6
Trauma	10	S5412XA	4
Trauma	10	S5012XA	7
Trauma	10	S42011B	1
Trauma	10	S21312A	8
Trauma	10	T24231D	1
Trauma	10	S32021A	23

© 2021 Pelletier et al. *JAMA Network Open.*

Diagnosis	ICD Version	ICD Code	Number of Encounters
Trauma	10	S82141B	5
Trauma	10	S40822A	1
Trauma	10	S02411B	9
Trauma	10	S49091A	4
Trauma	10	S72114A	1
Trauma	10	S90452A	4
Trauma	10	S92314D	1
Trauma	10	S82222C	3
Trauma	10	S31103A	4
Trauma	10	S92111A	11
Trauma	10	S92144A	2
Trauma	10	T23371A	3
Trauma	10	S62331B	3
Trauma	10	S81841A	9
Trauma	10	T25392A	21
Trauma	10	S36269A	2
Trauma	10	S61152A	1
Trauma	10	S0291XK	1
Trauma	10	S7221XB	2
Trauma	10	T25391A	12

Diagnosis	ICD Version	ICD Code	Number of Encounters
Trauma	10	S11022A	2
Trauma	10	S37091A	6
Trauma	10	S72365A	1
Trauma	10	S82092B	3
Trauma	10	T23311A	3
Trauma	10	S50311A	6
Trauma	10	S42411G	5
Trauma	10	S60472A	1
Trauma	10	S82202E	2
Trauma	10	S42031B	3
Trauma	10	S71152A	15
Trauma	10	S34103A	5
Trauma	10	S61256A	4
Trauma	10	S91102A	2
Trauma	10	S02610A	2
Trauma	10	S1194XA	2
Trauma	10	T22011A	8
Trauma	10	S66190A	1
Trauma	10	T23392A	14
Trauma	10	S52221B	12

© 2021 Pelletier et al. *JAMA Network Open.*

Diagnosis	ICD Version	ICD Code	Number of Encounters
Trauma	10	S92302B	1
Trauma	10	S179XXA	1
Trauma	10	S82292P	2
Trauma	10	S92342B	7
Trauma	10	T2139XA	8
Trauma	10	S8261XC	1
Trauma	10	T2115XA	2
Trauma	10	S68022A	7
Trauma	10	T33532A	4
Trauma	10	S06377A	1
Trauma	10	T23312A	3
Trauma	10	S14154A	8
Trauma	10	S66221A	3
Trauma	10	T2611XA	5
Trauma	10	T2136XA	2
Trauma	10	T22242A	2
Trauma	10	S82871C	1
Trauma	10	T22342A	4
Trauma	10	T2114XA	3
Trauma	10	S61203A	3

© 2021 Pelletier et al. *JAMA Network Open.*

Diagnosis	ICD Version	ICD Code	Number of Encounters
Trauma	10	S42291B	4
Trauma	10	S21441A	4
Trauma	10	S069X9S	7
Trauma	10	T25311D	1
Trauma	10	S2699XA	4
Trauma	10	T25312D	1
Trauma	10	T25312A	12
Trauma	10	T25331D	1
Trauma	10	T22241A	4
Trauma	10	T2154XA	1
Trauma	10	T22332D	2
Trauma	10	S68621A	11
Trauma	10	S62353B	1
Trauma	10	T22052A	5
Trauma	10	S91052A	1
Trauma	10	T148	15
Trauma	10	S72414A	3
Trauma	10	S92112A	12
Trauma	10	T22391D	1
Trauma	10	T2034XA	2

© 2021 Pelletier et al. *JAMA Network Open.*

Diagnosis	ICD Version	ICD Code	Number of Encounters
Trauma	10	T24722A	1
Trauma	10	T2602XA	7
Trauma	10	S90811A	3
Trauma	10	T24632A	1
Trauma	10	S72301C	3
Trauma	10	T25721A	2
Trauma	10	T33532D	2
Trauma	10	T33832A	4
Trauma	10	T33822A	4
Trauma	10	S62012A	3
Trauma	10	T3170	2
Trauma	10	T22392S	1
Trauma	10	S36499A	13
Trauma	10	S069X4A	2
Trauma	10	T3371XA	1
Trauma	10	S72432B	6
Trauma	10	T24702A	2
Trauma	10	S52181A	4
Trauma	10	T2066XA	2
Trauma	10	S32435A	4

© 2021 Pelletier et al. *JAMA Network Open.*

Diagnosis	ICD Version	ICD Code	Number of Encounters
Trauma	10	T25231A	3
Trauma	10	T23342A	5
Trauma	10	S42402P	3
Trauma	10	S93325A	2
Trauma	10	S2341XA	1
Trauma	10	S70212A	2
Trauma	10	S06380A	4
Trauma	10	S91109A	1
Trauma	10	S52355B	1
Trauma	10	S36299D	1
Trauma	10	S82832B	13
Trauma	10	S91311S	1
Trauma	10	S62610A	2
Trauma	10	S72021B	1
Trauma	10	S89201A	1
Trauma	10	S3720XA	3
Trauma	10	S46322A	3
Trauma	10	S51811D	1
Trauma	10	S0922XA	4
Trauma	10	S91012D	1

© 2021 Pelletier et al. *JAMA Network Open.*

Diagnosis	ICD Version	ICD Code	Number of Encounters
Trauma	10	S0210XD	2
Trauma	10	S52302K	2
Trauma	10	S060X2A	3
Trauma	10	S52301P	3
Trauma	10	S52341K	1
Trauma	10	S92421K	1
Trauma	10	S24103D	4
Trauma	10	S069X6S	4
Trauma	10	S92315B	3
Trauma	10	S42331B	3
Trauma	10	S31154A	2
Trauma	10	T23052A	6
Trauma	10	S60512A	5
Trauma	10	S50812A	4
Trauma	10	S80212A	8
Trauma	10	S24102D	5
Trauma	10	S60463A	1
Trauma	10	S72424K	1
Trauma	10	S72302S	1
Trauma	10	S43492A	4

© 2021 Pelletier et al. *JAMA Network Open.*

Diagnosis	ICD Version	ICD Code	Number of Encounters
Trauma	10	T182XXD	4
Trauma	10	S069X3A	2
Trauma	10	T1512XA	2
Trauma	10	S06366D	1
Trauma	10	S343XXD	1
Trauma	10	S72402D	2
Trauma	10	S62322A	3
Trauma	10	S062X6S	2
Trauma	10	S72455E	1
Trauma	10	S36539D	1
Trauma	10	S52309A	3
Trauma	10	S02113D	5
Trauma	10	S52201P	2
Trauma	10	S066X5D	3
Trauma	10	S02413B	4
Trauma	10	S60551A	3
Trauma	10	S06897A	11
Trauma	10	S061X9D	4
Trauma	10	S55102A	1
Trauma	10	S0990XS	3

© 2021 Pelletier et al. *JAMA Network Open.*

Diagnosis	ICD Version	ICD Code	Number of Encounters
Trauma	10	S22018D	1
Trauma	10	S06389D	1
Trauma	10	S92521B	1
Trauma	10	S01521A	6
Trauma	10	S89022S	1
Trauma	10	S79091A	5
Trauma	10	S14106D	2
Trauma	10	S62617B	6
Trauma	10	S61245A	1
Trauma	10	S72112A	10
Trauma	10	S58011A	4
Trauma	10	S0261XB	5
Trauma	10	S52251A	2
Trauma	10	S24153D	8
Trauma	10	S82854A	1
Trauma	10	S12090D	1
Trauma	10	S069X6D	2
Trauma	10	S68110D	2
Trauma	10	S2329XA	2
Trauma	10	S32402D	6

© 2021 Pelletier et al. *JAMA Network Open.*

Diagnosis	ICD Version	ICD Code	Number of Encounters
Trauma	10	S61011A	3
Trauma	10	S82291C	4
Trauma	10	S064X6D	1
Trauma	10	S82845A	1
Trauma	10	S81001D	3
Trauma	10	S066X7D	1
Trauma	10	S12430A	3
Trauma	10	S066X8A	4
Trauma	10	S97111A	2
Trauma	10	S280XXA	18
Trauma	10	T25292D	1
Trauma	10	S02609D	3
Trauma	10	S42412K	1
Trauma	10	S01512D	3
Trauma	10	S52131P	2
Trauma	10	S62622A	2
Trauma	10	S42409A	3
Trauma	10	S42411S	1
Trauma	10	S134XXD	6
Trauma	10	S066X1S	1

© 2021 Pelletier et al. *JAMA Network Open.*

Diagnosis	ICD Version	ICD Code	Number of Encounters
Trauma	10	S3719XA	6
Trauma	10	S92501B	1
Trauma	10	S32018D	3
Trauma	10	S06324D	1
Trauma	10	S62521B	12
Trauma	10	S61112A	2
Trauma	10	S32058D	2
Trauma	10	S06899D	19
Trauma	10	S066X6S	1
Trauma	10	S06329D	3
Trauma	10	S062X5D	6
Trauma	10	S090XXA	5
Trauma	10	S13120D	5
Trauma	10	S33131S	1
Trauma	10	S02119D	3
Trauma	10	S02109D	2
Trauma	10	S61252A	5
Trauma	10	S064X5D	1
Trauma	10	S72302J	1
Trauma	10	S72302H	2

© 2021 Pelletier et al. *JAMA Network Open.*

Diagnosis	ICD Version	ICD Code	Number of Encounters
Trauma	10	S25101A	1
Trauma	10	S52614A	1
Trauma	10	T24392D	2
Trauma	10	S66123A	3
Trauma	10	S82262C	3
Trauma	10	S24153A	9
Trauma	10	S00261A	7
Trauma	10	S85011D	1
Trauma	10	S3719XS	1
Trauma	10	S9421XA	1
Trauma	10	S76192A	3
Trauma	10	S0990XD	2
Trauma	10	S14104D	3
Trauma	10	S14117D	1
Trauma	10	S02101B	2
Trauma	10	S66115D	1
Trauma	10	S06375A	1
Trauma	10	S6412XD	1
Trauma	10	S14156D	6
Trauma	10	S0211HB	9

© 2021 Pelletier et al. *JAMA Network Open.*

Diagnosis	ICD Version	ICD Code	Number of Encounters
Trauma	10	S27322D	1
Trauma	10	S62313B	3
Trauma	10	S62021K	1
Trauma	10	S32511D	3
Trauma	10	S6432XA	2
Trauma	10	S14107A	3
Trauma	10	S62324A	2
Trauma	10	T286XXS	2
Trauma	10	S64492A	4
Trauma	10	S12600D	3
Trauma	10	S32049D	3
Trauma	10	S12110D	1
Trauma	10	S37021S	1
Trauma	10	S12490D	3
Trauma	10	S06356D	2
Trauma	10	S92242A	1
Trauma	10	S31819A	4
Trauma	10	S14155D	5
Trauma	10	S92131A	1
Trauma	10	S06369D	14

© 2021 Pelletier et al. *JAMA Network Open.*

Diagnosis	ICD Version	ICD Code	Number of Encounters
Trauma	10	S62304K	1
Trauma	10	S22029D	1
Trauma	10	S52502E	1
Trauma	10	S32592D	5
Trauma	10	S92202A	1
Trauma	10	S62330A	2
Trauma	10	S7292XK	5
Trauma	10	S7292XE	2
Trauma	10	S73004S	3
Trauma	10	S83412A	4
Trauma	10	S61214A	5
Trauma	10	S22039D	1
Trauma	10	S61442A	7
Trauma	10	S22068D	1
Trauma	10	S49012A	4
Trauma	10	S12431A	1
Trauma	10	S59021A	4
Trauma	10	S12690D	4
Trauma	10	S2243XD	2
Trauma	10	S91042A	6

© 2021 Pelletier et al. *JAMA Network Open.*

Diagnosis	ICD Version	ICD Code	Number of Encounters
Trauma	10	S24101A	6
Trauma	10	S24101D	1
Trauma	10	S82254B	3
Trauma	10	S76811A	2
Trauma	10	S93121A	1
Trauma	10	S42461B	5
Trauma	10	S52592P	1
Trauma	10	S02119S	2
Trauma	10	S0100XS	2
Trauma	10	S52501P	4
Trauma	10	S72111F	1
Trauma	10	S52325B	2
Trauma	10	S8012XD	1
Trauma	10	S82025A	1
Trauma	10	S0541XA	16
Trauma	10	S82191B	3
Trauma	10	S83105A	6
Trauma	10	S02412D	1
Trauma	10	S06379D	3
Trauma	10	S12091A	6

© 2021 Pelletier et al. *JAMA Network Open.*

Diagnosis	ICD Version	ICD Code	Number of Encounters
Trauma	10	S22061D	1
Trauma	10	S8265XA	3
Trauma	10	S6982XA	5
Trauma	10	S56123A	1
Trauma	10	S81031A	4
Trauma	10	S82242D	1
Trauma	10	S62636A	2
Trauma	10	S82222K	1
Trauma	10	S12291A	2
Trauma	10	S92332A	2
Trauma	10	S62012K	2
Trauma	10	S24151D	3
Trauma	10	S33131D	1
Trauma	10	S52222A	14
Trauma	10	S7222XE	1
Trauma	10	S52372B	7
Trauma	10	T2030XD	2
Trauma	10	S73035A	8
Trauma	10	S71102D	2
Trauma	10	S72141E	2

© 2021 Pelletier et al. *JAMA Network Open.*

Diagnosis	ICD Version	ICD Code	Number of Encounters
Trauma	10	S40212A	7
Trauma	10	S25121A	1
Trauma	10	S0267XB	1
Trauma	10	S22069D	1
Trauma	10	S42202D	1
Trauma	10	S060X9S	2
Trauma	10	S61451S	3
Trauma	10	S51042A	5
Trauma	10	S06360D	4
Trauma	10	S62231P	1
Trauma	10	S72392B	1
Trauma	10	S72321P	1
Trauma	10	S37039A	3
Trauma	10	S52354A	1
Trauma	10	S50862A	8
Trauma	10	S62634A	4
Trauma	10	S41012A	4
Trauma	10	S61231A	7
Trauma	10	S83092A	1
Trauma	10	S82192S	1

© 2021 Pelletier et al. *JAMA Network Open.*

Diagnosis	ICD Version	ICD Code	Number of Encounters
Trauma	10	S0190XD	1
Trauma	10	S32612A	7
Trauma	10	S92515A	1
Trauma	10	S3730XA	15
Trauma	10	S31604A	4
Trauma	10	T2112XA	4
Trauma	10	S71121D	1
Trauma	10	S72325S	1
Trauma	10	S66320A	7
Trauma	10	S80262A	2
Trauma	10	S61552A	7
Trauma	10	S0282XB	10
Trauma	10	S058X1S	1
Trauma	10	S32611A	3
Trauma	10	S36592A	3
Trauma	10	T24092A	2
Trauma	10	T24291D	2
Trauma	10	S02672B	5
Trauma	10	S32811D	2
Trauma	10	S728X1B	1

© 2021 Pelletier et al. *JAMA Network Open.*

Diagnosis	ICD Version	ICD Code	Number of Encounters
Trauma	10	S50861A	3
Trauma	10	S40862A	2
Trauma	10	S31610A	2
Trauma	10	S62171B	1
Trauma	10	S81831A	7
Trauma	10	T23211A	2
Trauma	10	S01151S	1
Trauma	10	T23392D	1
Trauma	10	S42035B	2
Trauma	10	S76112A	7
Trauma	10	S49092A	4
Trauma	10	S49142A	5
Trauma	10	S72112B	2
Trauma	10	S60410A	1
Trauma	10	T23222A	3
Trauma	10	S61230A	2
Trauma	10	S32611D	1
Trauma	10	S60541A	1
Trauma	10	S90562A	5
Trauma	10	S36116D	3

© 2021 Pelletier et al. *JAMA Network Open.*

Diagnosis	ICD Version	ICD Code	Number of Encounters
Trauma	10	S60561A	6
Trauma	10	S52382B	2
Trauma	10	T23271A	5
Trauma	10	S1081XA	12
Trauma	10	S32491A	6
Trauma	10	S52392A	12
Trauma	10	T161XXA	10
Trauma	10	S93692A	1
Trauma	10	S66892A	2
Trauma	10	S60352A	1
Trauma	10	S50361A	1
Trauma	10	S06346D	1
Trauma	10	S92401A	1
Trauma	10	S82114C	1
Trauma	10	S069X0S	4
Trauma	10	S35511A	5
Trauma	10	S55012A	5
Trauma	10	S31832A	1
Trauma	10	S88011A	2
Trauma	10	S88011D	1

© 2021 Pelletier et al. *JAMA Network Open.*

Diagnosis	ICD Version	ICD Code	Number of Encounters
Trauma	10	S62665B	1
Trauma	10	S43402A	1
Trauma	10	T274XXS	1
Trauma	10	S92415B	1
Trauma	10	T2167XA	4
Trauma	10	S61215A	5
Trauma	10	S52354C	1
Trauma	10	S59811A	1
Trauma	10	S60440A	1
Trauma	10	S058X2A	7
Trauma	10	S91115A	2
Trauma	10	S72132A	3
Trauma	10	S064X7A	5
Trauma	10	S14122A	4
Trauma	10	S72442B	3
Trauma	10	S42461P	1
Trauma	10	S72332K	2
Trauma	10	S42295A	5
Trauma	10	S40012A	3
Trauma	10	T24312D	2

© 2021 Pelletier et al. *JAMA Network Open.*

Diagnosis	ICD Version	ICD Code	Number of Encounters
Trauma	10	S42401D	1
Trauma	10	S81801D	7
Trauma	10	S61421A	6
Trauma	10	S0120XA	2
Trauma	10	S72401P	3
Trauma	10	T23151A	2
Trauma	10	S61232A	3
Trauma	10	S79019A	11
Trauma	10	S68612A	4
Trauma	10	S08811D	1
Trauma	10	S92012B	1
Trauma	10	S76822A	6
Trauma	10	S96192A	1
Trauma	10	S92331A	4
Trauma	10	T24301D	2
Trauma	10	S71142A	15
Trauma	10	S06899S	7
Trauma	10	S62625A	2
Trauma	10	S82233A	1
Trauma	10	S01322A	1

© 2021 Pelletier et al. *JAMA Network Open.*

Diagnosis	ICD Version	ICD Code	Number of Encounters
Trauma	10	S42473A	1
Trauma	10	S92041B	1
Trauma	10	S13161A	4
Trauma	10	S83519A	1
Trauma	10	S35411A	2
Trauma	10	S62650B	2
Trauma	10	S32312B	1
Trauma	10	S0211CA	3
Trauma	10	S5011XA	3
Trauma	10	S92251B	2
Trauma	10	S36593A	8
Trauma	10	S31614A	6
Trauma	10	S89132P	1
Trauma	10	S1097XA	3
Trauma	10	S14112A	2
Trauma	10	S52124A	4
Trauma	10	S32111A	3
Trauma	10	S14117A	2
Trauma	10	S14152A	11
Trauma	10	S0512XD	3

© 2021 Pelletier et al. *JAMA Network Open.*

Diagnosis	ICD Version	ICD Code	Number of Encounters
Trauma	10	S14105S	2
Trauma	10	S32059K	2
Trauma	10	S32052A	5
Trauma	10	S72041K	2
Trauma	10	S79092A	5
Trauma	10	S72024A	2
Trauma	10	S82455A	2
Trauma	10	T183XXD	2
Trauma	10	S32391B	5
Trauma	10	S01351D	2
Trauma	10	S8261XB	4
Trauma	10	S62232B	3
Trauma	10	S36230A	10
Trauma	10	S31129A	1
Trauma	10	S2690XA	4
Trauma	10	S91321S	1
Trauma	10	S12191A	7
Trauma	10	S91312D	4
Trauma	10	T25291S	1
Trauma	10	S68019A	1

© 2021 Pelletier et al. *JAMA Network Open.*

Diagnosis	ICD Version	ICD Code	Number of Encounters
Trauma	10	S72491G	2
Trauma	10	S139XXA	19
Trauma	10	T285XXA	3
Trauma	10	S52309C	1
Trauma	10	S1201XA	3
Trauma	10	S72321C	5
Trauma	10	S42311A	1
Trauma	10	T1580XA	2
Trauma	10	T24622A	1
Trauma	10	T3188	1
Trauma	10	T277XXA	1
Trauma	10	S43121A	1
Trauma	10	S32401A	9
Trauma	10	S0185XD	5
Trauma	10	T320	1
Trauma	10	T2172XA	4
Trauma	10	S300XXD	1
Trauma	10	S85002A	1
Trauma	10	S21102A	3
Trauma	10	S31101A	2

© 2021 Pelletier et al. *JAMA Network Open.*

Diagnosis	ICD Version	ICD Code	Number of Encounters
Trauma	10	S13130A	3
Trauma	10	S86891A	1
Trauma	10	T24002A	3
Trauma	10	S59102A	2
Trauma	10	S31645A	1
Trauma	10	S3501XA	4
Trauma	10	T33522A	2
Trauma	10	T23229A	1
Trauma	10	S0211FA	5
Trauma	10	T22399A	2
Trauma	10	S82422B	3
Trauma	10	S36522A	5
Trauma	10	T2130XA	1
Trauma	10	S82202S	1
Trauma	10	T3199	2
Trauma	10	S91155A	1
Trauma	10	T24701A	1
Trauma	10	T3190	1
Trauma	10	S3509XA	7
Trauma	10	S2600XA	1

© 2021 Pelletier et al. *JAMA Network Open.*

Diagnosis	ICD Version	ICD Code	Number of Encounters
Trauma	10	S81812S	4
Trauma	10	S34112A	1
Trauma	10	S0193XA	7
Trauma	10	S32028B	2
Trauma	10	T24301S	1
Trauma	10	T25012A	4
Trauma	10	T23602A	2
Trauma	10	S58012A	2
Trauma	10	S52231C	1
Trauma	10	T271XXA	5
Trauma	10	S37062S	1
Trauma	10	S62172B	2
Trauma	10	S66922A	2
Trauma	10	T22032A	3
Trauma	10	S20219S	1
Trauma	10	S85011A	4
Trauma	10	S52302H	1
Trauma	10	S72425B	1
Trauma	10	S0211DA	2
Trauma	10	S52511B	3

© 2021 Pelletier et al. *JAMA Network Open.*

Diagnosis	ICD Version	ICD Code	Number of Encounters
Trauma	10	S061X2A	2
Trauma	10	S0280XB	2
Trauma	10	T2166XA	1
Trauma	10	S02412B	4
Trauma	10	S82451B	5
Trauma	10	S86012A	1
Trauma	10	S21131A	9
Trauma	10	S62253A	1
Trauma	10	T25722A	2
Trauma	10	S92425B	4
Trauma	10	S06819A	1
Trauma	10	S82402C	2
Trauma	10	S0103XA	4
Trauma	10	S6741XA	6
Trauma	10	S82391C	6
Trauma	10	S72121A	5
Trauma	10	S32031B	1
Trauma	10	S62319A	1
Trauma	10	S21101A	5
Trauma	10	S00572A	1

© 2021 Pelletier et al. *JAMA Network Open.*

Diagnosis	ICD Version	ICD Code	Number of Encounters
Trauma	10	S36523A	2
Trauma	10	S34121A	5
Trauma	10	S62211B	1
Trauma	10	S92211B	3
Trauma	10	S68118A	1
Trauma	10	S93402A	10
Trauma	10	S36128A	1
Trauma	10	S02622B	6
Trauma	10	S52501C	10
Trauma	10	S21132A	8
Trauma	10	S61412S	1
Trauma	10	S42115A	2
Trauma	10	S32461A	7
Trauma	10	S82041C	1
Trauma	10	S81811S	2
Trauma	10	S62292B	1
Trauma	10	S91105A	2
Trauma	10	S62313A	3
Trauma	10	T25692A	1
Trauma	10	S52132D	3

© 2021 Pelletier et al. *JAMA Network Open.*

Diagnosis	ICD Version	ICD Code	Number of Encounters
Trauma	10	S82431B	1
Trauma	10	S0232XK	1
Trauma	10	S1982XA	5
Trauma	10	S42009A	2
Trauma	10	T24311D	2
Trauma	10	S3120XD	1
Trauma	10	S06811A	1
Trauma	10	S62601B	2
Trauma	10	S82302S	1
Trauma	10	T2063XD	1
Trauma	10	S92355A	2
Trauma	10	S66121A	4
Trauma	10	S42452G	2
Trauma	10	S72001C	1
Trauma	10	S96901A	1
Trauma	10	S46212A	1
Trauma	10	S61210A	4
Trauma	10	S142XXA	6
Trauma	10	S79921A	1
Trauma	10	T2612XA	3

© 2021 Pelletier et al. *JAMA Network Open.*

Diagnosis	ICD Version	ICD Code	Number of Encounters
Trauma	10	S81841D	1
Trauma	10	S06890S	10
Trauma	10	S72492P	1
Trauma	10	T07XXXA	1
Trauma	10	T190XXA	5
Trauma	10	S01442A	2
Trauma	10	S0240EB	6
Trauma	10	S92424A	1
Trauma	10	S62617A	8
Trauma	10	S1011XA	2
Trauma	10	S39840A	3
Trauma	10	S02110A	3
Trauma	10	S81801S	2
Trauma	10	S82442A	1
Trauma	10	S3132XA	3
Trauma	10	S81811D	4
Trauma	10	S42471A	3
Trauma	10	S89191A	15
Trauma	10	T2159XA	1
Trauma	10	S72452B	2

© 2021 Pelletier et al. *JAMA Network Open.*

Diagnosis	ICD Version	ICD Code	Number of Encounters
Trauma	10	S72102A	8
Trauma	10	S34109A	6
Trauma	10	S0030XA	1
Trauma	10	S52391A	9
Trauma	10	S52331A	8
Trauma	10	S66125A	4
Trauma	10	S62015A	1
Trauma	10	S01301A	4
Trauma	10	S42421G	2
Trauma	10	S01132A	2
Trauma	10	S42312A	1
Trauma	10	S2232XD	1
Trauma	10	S3983XA	5
Trauma	10	T17800A	5
Trauma	10	S90422A	1
Trauma	10	S90861A	2
Trauma	10	S62314B	1
Trauma	10	S00571A	1
Trauma	10	S1984XA	1
Trauma	10	S51819A	1

© 2021 Pelletier et al. *JAMA Network Open.*

Diagnosis	ICD Version	ICD Code	Number of Encounters
Trauma	10	S68127A	10
Trauma	10	S60444A	1
Trauma	10	S59121A	5
Trauma	10	S00272A	1
Trauma	10	S62627A	1
Trauma	10	S36438D	1
Trauma	10	S70361A	5
Trauma	10	S93315A	2
Trauma	10	S41001A	5
Trauma	10	T17590D	1
Trauma	10	S31139A	7
Trauma	10	S069X2A	2
Trauma	10	S60221A	3
Trauma	10	S41101A	5
Trauma	10	S82145P	1
Trauma	10	S37009A	2
Trauma	10	S68422A	3
Trauma	10	S29001A	1
Trauma	10	S82425A	2
Trauma	10	S91122A	1

© 2021 Pelletier et al. *JAMA Network Open.*

Diagnosis	ICD Version	ICD Code	Number of Encounters
Trauma	10	S93324A	2
Trauma	10	S62316A	2
Trauma	10	S32425A	2
Trauma	10	S90821A	5
Trauma	10	S72444A	3
Trauma	10	S82841P	1
Trauma	10	S53005D	1
Trauma	10	S42342B	1
Trauma	10	S82454A	1
Trauma	10	S82124A	1
Trauma	10	S21002A	1
Trauma	10	S61012A	4
Trauma	10	S56521A	4
Trauma	10	S82871D	3
Trauma	10	S60562A	8
Trauma	10	S82864A	1
Trauma	10	S66324A	4
Trauma	10	S61419A	1
Trauma	10	S27892A	2
Trauma	10	S62501B	1

© 2021 Pelletier et al. *JAMA Network Open.*

Diagnosis	ICD Version	ICD Code	Number of Encounters
Trauma	10	S83200A	1
Trauma	10	S9701XA	2
Trauma	10	S92402B	2
Trauma	10	S31834A	1
Trauma	10	T2161XA	2
Trauma	10	S73011A	4
Trauma	10	S82401K	1
Trauma	10	S82032B	4
Trauma	10	S42411P	4
Trauma	10	S60852A	1
Trauma	10	S52202E	1
Trauma	10	S52362B	1
Trauma	10	S62304A	2
Trauma	10	S92335A	1
Trauma	10	S52512A	5
Trauma	10	S12030A	7
Trauma	10	S12401A	8
Trauma	10	S0100XD	1
Trauma	10	S13110S	2
Trauma	10	S52302G	4

© 2021 Pelletier et al. *JAMA Network Open.*

Diagnosis	ICD Version	ICD Code	Number of Encounters
Trauma	10	S82141P	2
Trauma	10	S50851A	2
Trauma	10	S82141C	1
Trauma	10	S62616B	2
Trauma	10	S62640A	1
Trauma	10	S8422XA	1
Trauma	10	S31050A	1
Trauma	10	S51802A	5
Trauma	10	S43014A	2
Trauma	10	S32512G	1
Trauma	10	S72141D	1
Trauma	10	S4431XA	1
Trauma	10	S81852D	3
Trauma	10	S31603A	5
Trauma	10	S92191A	1
Trauma	10	S0240BA	2
Trauma	10	S02600B	9
Trauma	10	S1190XD	1
Trauma	10	S42021B	4
Trauma	10	S21309A	1

© 2021 Pelletier et al. *JAMA Network Open.*

Diagnosis	ICD Version	ICD Code	Number of Encounters
Trauma	10	S35412A	2
Trauma	10	S24104D	4
Trauma	10	T24092D	1
Trauma	10	S3289XB	3
Trauma	10	S06896A	4
Trauma	10	S14159A	5
Trauma	10	T25621A	1
Trauma	10	S12121A	2
Trauma	10	S37022D	1
Trauma	10	S92155B	1
Trauma	10	S92912A	1
Trauma	10	S06312A	1
Trauma	10	S56222A	6
Trauma	10	S0211EA	5
Trauma	10	S3134XA	5
Trauma	10	S82852B	2
Trauma	10	S82201D	5
Trauma	10	S92121B	2
Trauma	10	S62315A	2
Trauma	10	S86122A	4

© 2021 Pelletier et al. *JAMA Network Open.*

Diagnosis	ICD Version	ICD Code	Number of Encounters
Trauma	10	S21242A	2
Trauma	10	S82242B	3
Trauma	10	S62201A	2
Trauma	10	S7291XK	4
Trauma	10	S45211A	3
Trauma	10	S62633G	3
Trauma	10	T22112A	1
Trauma	10	S7011XD	2
Trauma	10	S34123S	1
Trauma	10	T25021A	6
Trauma	10	T1491XD	1
Trauma	10	S52202C	4
Trauma	10	S96822A	3
Trauma	10	T2075XA	2
Trauma	10	S01411D	2
Trauma	10	S82102K	3
Trauma	10	S42114A	4
Trauma	10	S0571XA	3
Trauma	10	S37822A	1
Trauma	10	S52542B	1

© 2021 Pelletier et al. *JAMA Network Open.*

Diagnosis	ICD Version	ICD Code	Number of Encounters
Trauma	10	S41122S	1
Trauma	10	S225XXA	6
Trauma	10	S34132A	1
Trauma	10	S12690B	1
Trauma	10	T22611A	2
Trauma	10	S40261A	1
Trauma	10	S31104D	1
Trauma	10	S89102P	1
Trauma	10	S0190XA	3
Trauma	10	S31030A	2
Trauma	10	S61217A	2
Trauma	10	S52121A	19
Trauma	10	S0240DG	1
Trauma	10	S52602C	1
Trauma	10	S60522D	1
Trauma	10	T23011A	1
Trauma	10	S62511A	9
Trauma	10	S52511A	8
Trauma	10	S52541A	1
Trauma	10	S35331A	2

© 2021 Pelletier et al. *JAMA Network Open.*

Diagnosis	ICD Version	ICD Code	Number of Encounters
Trauma	10	S92411A	5
Trauma	10	S81052A	8
Trauma	10	S68412A	1
Trauma	10	S92912B	2
Trauma	10	S82231C	6
Trauma	10	T2032XA	3
Trauma	10	S21152A	1
Trauma	10	S2691XA	9
Trauma	10	S31159A	2
Trauma	10	S62291B	1
Trauma	10	S81851S	2
Trauma	10	S71111D	1
Trauma	10	S65111A	3
Trauma	10	S91051D	1
Trauma	10	T24611A	1
Trauma	10	S60572A	3
Trauma	10	S42035A	1
Trauma	10	S2223XK	2
Trauma	10	S14152D	2
Trauma	10	S85911A	1

© 2021 Pelletier et al. *JAMA Network Open.*

Diagnosis	ICD Version	ICD Code	Number of Encounters
Trauma	10	S31619A	8
Trauma	10	S72142B	3
Trauma	10	T2165XA	6
Trauma	10	T24212D	1
Trauma	10	S4422XA	3
Trauma	10	S62311B	1
Trauma	10	S31144A	6
Trauma	10	S0531XS	1
Trauma	10	S42448B	2
Trauma	10	S66120A	3
Trauma	10	S42292B	1
Trauma	10	S3021XA	9
Trauma	10	S9782XD	1
Trauma	10	T22331D	2
Trauma	10	S72424C	1
Trauma	10	S90454A	1
Trauma	10	T2111XA	3
Trauma	10	S72122A	7
Trauma	10	S7222XS	1
Trauma	10	T23102A	1

© 2021 Pelletier et al. *JAMA Network Open.*

Diagnosis	ICD Version	ICD Code	Number of Encounters
Trauma	10	S30863A	2
Trauma	10	S0450XA	1
Trauma	10	T23031A	2
Trauma	10	S51851S	2
Trauma	10	S41041A	2
Trauma	10	S68614A	3
Trauma	10	S76212A	1
Trauma	10	S75091A	3
Trauma	10	T25212D	1
Trauma	10	S22072A	1
Trauma	10	S42122B	2
Trauma	10	S42033A	1
Trauma	10	S12031A	5
Trauma	10	S82445A	1
Trauma	10	S06893A	1
Trauma	10	S81802D	4
Trauma	10	T2200XD	4
Trauma	10	S31612A	3
Trauma	10	S062X2S	1
Trauma	10	S70321A	1

© 2021 Pelletier et al. *JAMA Network Open.*

Diagnosis	ICD Version	ICD Code	Number of Encounters
Trauma	10	S82391P	1
Trauma	10	S50871A	1
Trauma	10	T22612A	1
Trauma	10	S069X8A	2
Trauma	10	T170XXA	7
Trauma	10	T2040XA	1
Trauma	10	S68011A	14
Trauma	10	S71151S	2
Trauma	10	T3150	1
Trauma	10	S1091XA	23
Trauma	10	S62637A	3
Trauma	10	S13131D	1
Trauma	10	S82492B	2
Trauma	10	S21322A	1
Trauma	10	T23201D	2
Trauma	10	S91134A	1
Trauma	10	T23501A	1
Trauma	10	S90425A	2
Trauma	10	S62251B	1
Trauma	10	T2220XD	1

© 2021 Pelletier et al. *JAMA Network Open.*

Diagnosis	ICD Version	ICD Code	Number of Encounters
Trauma	10	T2067XA	1
Trauma	10	S14157A	5
Trauma	10	S2509XA	7
Trauma	10	T22021A	3
Trauma	10	S31815A	3
Trauma	10	S062X1S	3
Trauma	10	S82891C	4
Trauma	10	S45012A	2
Trauma	10	S32401D	1
Trauma	10	S472XXA	2
Trauma	10	T2060XA	2
Trauma	10	S52281B	1
Trauma	10	S31152A	1
Trauma	10	S82222N	1
Trauma	10	T2120XD	1
Trauma	10	S20212D	1
Trauma	10	S98022A	3
Trauma	10	S72324C	1
Trauma	10	S83005S	1
Trauma	10	S31605A	1

© 2021 Pelletier et al. *JAMA Network Open.*

Diagnosis	ICD Version	ICD Code	Number of Encounters
Trauma	10	S0240AA	1
Trauma	10	S86921A	4
Trauma	10	S13151A	4
Trauma	10	S12290A	5
Trauma	10	S82241B	2
Trauma	10	S63035A	1
Trauma	10	S32415A	2
Trauma	10	S32455A	2
Trauma	10	S6401XA	5
Trauma	10	S45091A	2
Trauma	10	S98922A	2
Trauma	10	S46221A	3
Trauma	10	S52332A	6
Trauma	10	S52324A	2
Trauma	10	S3125XA	1
Trauma	10	S3690XA	1
Trauma	10	S72461A	4
Trauma	10	S72421C	2
Trauma	10	S52122B	3
Trauma	10	S061X5A	1

© 2021 Pelletier et al. *JAMA Network Open.*

Diagnosis	ICD Version	ICD Code	Number of Encounters
Trauma	10	S21331A	4
Trauma	10	S72352N	2
Trauma	10	S02601K	2
Trauma	10	S82841M	1
Trauma	10	S00412A	3
Trauma	10	S7712XA	2
Trauma	10	S062X3A	4
Trauma	10	S32392B	4
Trauma	10	S30822A	1
Trauma	10	S31642A	3
Trauma	10	S13131A	1
Trauma	10	S32810B	4
Trauma	10	S42022B	4
Trauma	10	S13121D	1
Trauma	10	S3739XD	1
Trauma	10	S27432A	2
Trauma	10	S52182B	3
Trauma	10	S42034A	3
Trauma	10	S72035A	5
Trauma	10	S8251XC	3

© 2021 Pelletier et al. *JAMA Network Open.*

Diagnosis	ICD Version	ICD Code	Number of Encounters
Trauma	10	S32591D	4
Trauma	10	S06349D	4
Trauma	10	S129XXD	2
Trauma	10	S86322A	1
Trauma	10	S61223A	1
Trauma	10	S72421B	1
Trauma	10	S21011A	1
Trauma	10	S93401D	1
Trauma	10	S12491S	1
Trauma	10	S72451C	1
Trauma	10	S82201M	3
Trauma	10	S72092C	1
Trauma	10	S06320D	1
Trauma	10	S79912A	6
Trauma	10	S90561A	1
Trauma	10	S35514A	1
Trauma	10	S138XXA	1
Trauma	10	S93114A	2
Trauma	10	S76921A	2
Trauma	10	S72346A	1

© 2021 Pelletier et al. *JAMA Network Open.*

Diagnosis	ICD Version	ICD Code	Number of Encounters
Trauma	10	S06350D	3
Trauma	10	S06810A	3
Trauma	10	S02112D	1
Trauma	10	S06337A	2
Trauma	10	S92061B	2
Trauma	10	S34102A	5
Trauma	10	S72091B	1
Trauma	10	S24112D	9
Trauma	10	S32019D	3
Trauma	10	S92352G	1
Trauma	10	S83011A	2
Trauma	10	S82201P	1
Trauma	10	S14153A	4
Trauma	10	S92321D	1
Trauma	10	S93409A	1
Trauma	10	S8263XA	1
Trauma	10	S31105A	1
Trauma	10	S01421A	8
Trauma	10	S82491B	2
Trauma	10	S60470A	1

© 2021 Pelletier et al. *JAMA Network Open.*

Diagnosis	ICD Version	ICD Code	Number of Encounters
Trauma	10	S20362A	2
Trauma	10	T1591XA	2
Trauma	10	S21209A	3
Trauma	10	S83003A	1
Trauma	10	S32601A	2
Trauma	10	S42302K	5
Trauma	10	S22041A	5
Trauma	10	S31140A	2
Trauma	10	S71021A	1
Trauma	10	S89012A	6
Trauma	10	S31131A	1
Trauma	10	S4432XA	1
Trauma	10	S0561XA	3
Trauma	10	S52002C	1
Trauma	10	S62301A	2
Trauma	10	S21121A	1
Trauma	10	S02841B	1
Trauma	10	S92341B	3
Trauma	10	S32301D	1
Trauma	10	T22259A	1

© 2021 Pelletier et al. *JAMA Network Open.*

Diagnosis	ICD Version	ICD Code	Number of Encounters
Trauma	10	S72002G	2
Trauma	10	S5291XK	1
Trauma	10	S82151K	2
Trauma	10	S96091A	1
Trauma	10	S80852A	2
Trauma	10	S91041A	4
Trauma	10	S72301E	2
Trauma	10	S32122A	1
Trauma	10	S88121A	2
Trauma	10	S72462B	2
Trauma	10	S62304B	1
Trauma	10	S59802A	1
Trauma	10	S42451P	3
Trauma	10	T22249A	1
Trauma	10	S00511A	4
Trauma	10	S028XXK	1
Trauma	10	S00542A	1
Trauma	10	S82492A	10
Trauma	10	T23242S	1
Trauma	10	S86211A	2

© 2021 Pelletier et al. *JAMA Network Open.*

Diagnosis	ICD Version	ICD Code	Number of Encounters
Trauma	10	S80251A	5
Trauma	10	S060X5A	1
Trauma	10	S42142A	4
Trauma	10	S31123A	1
Trauma	10	S42432K	1
Trauma	10	S12400B	1
Trauma	10	S06825A	1
Trauma	10	S82255A	5
Trauma	10	T24219A	2
Trauma	10	S3713XA	2
Trauma	10	S89292A	1
Trauma	10	S48111A	2
Trauma	10	S31644A	1
Trauma	10	S11025A	1
Trauma	10	S31659A	1
Trauma	10	S52509A	2
Trauma	10	T25322D	2
Trauma	10	S56991A	1
Trauma	10	T17210A	2
Trauma	10	S31641A	2

© 2021 Pelletier et al. *JAMA Network Open.*

Diagnosis	ICD Version	ICD Code	Number of Encounters
Trauma	10	S2619XS	1
Trauma	10	S62032A	1
Trauma	10	S32409A	2
Trauma	10	S52591C	1
Trauma	10	S36209D	1
Trauma	10	S72442C	1
Trauma	10	T2055XA	1
Trauma	10	S85172A	2
Trauma	10	S81852S	1
Trauma	10	S0003XD	2
Trauma	10	S01431A	2
Trauma	10	S82251N	1
Trauma	10	S13140A	3
Trauma	10	S62305A	2
Trauma	10	S52271P	8
Trauma	10	S22052A	3
Trauma	10	S82892B	3
Trauma	10	S21139A	3
Trauma	10	S52301C	4
Trauma	10	S79119A	1

© 2021 Pelletier et al. *JAMA Network Open.*

Diagnosis	ICD Version	ICD Code	Number of Encounters
Trauma	10	S72301B	15
Trauma	10	T22152A	1
Trauma	10	S0091XA	5
Trauma	10	S12490B	1
Trauma	10	S728X2D	3
Trauma	10	S3210XD	4
Trauma	10	T1592XA	3
Trauma	10	S68610A	7
Trauma	10	S8255XB	1
Trauma	10	S12201A	3
Trauma	10	S82401B	4
Trauma	10	S31103S	1
Trauma	10	S36598A	6
Trauma	10	S24152D	6
Trauma	10	S62304P	1
Trauma	10	S72351D	3
Trauma	10	S21012A	3
Trauma	10	S60451A	2
Trauma	10	S31825A	2
Trauma	10	S82021B	1

© 2021 Pelletier et al. *JAMA Network Open.*

Diagnosis	ICD Version	ICD Code	Number of Encounters
Trauma	10	S25511A	1
Trauma	10	S06371A	6
Trauma	10	S06328A	1
Trauma	10	S36418A	2
Trauma	10	S91051A	4
Trauma	10	S27893A	1
Trauma	10	S42301K	5
Trauma	10	S72431B	4
Trauma	10	T23121A	1
Trauma	10	S42361B	1
Trauma	10	S4412XA	4
Trauma	10	S06344A	1
Trauma	10	S061X8A	1
Trauma	10	S66127A	2
Trauma	10	S35515A	1
Trauma	10	T194XXA	1
Trauma	10	S43204A	1
Trauma	10	S52252C	2
Trauma	10	S90424A	3
Trauma	10	S1010XA	1

© 2021 Pelletier et al. *JAMA Network Open.*

Diagnosis	ICD Version	ICD Code	Number of Encounters
Trauma	10	S41122A	1
Trauma	10	S31041A	1
Trauma	10	S00531A	4
Trauma	10	T20111A	2
Trauma	10	S72331B	5
Trauma	10	S22011A	5
Trauma	10	S92531B	2
Trauma	10	S21219A	3
Trauma	10	S72331M	1
Trauma	10	S62623A	2
Trauma	10	S8412XA	6
Trauma	10	S82444A	2
Trauma	10	S40872A	1
Trauma	10	S72491D	1
Trauma	10	S72401D	1
Trauma	10	S92911A	1
Trauma	10	S82121B	1
Trauma	10	S7221XD	3
Trauma	10	S82235G	1
Trauma	10	S40821A	1

© 2021 Pelletier et al. *JAMA Network Open.*

Diagnosis	ICD Version	ICD Code	Number of Encounters
Trauma	10	S21051A	1
Trauma	10	S71022A	1
Trauma	10	S1189XD	1
Trauma	10	S0006XA	2
Trauma	10	S52225K	1
Trauma	10	S49109A	1
Trauma	10	S061X3A	1
Trauma	10	S73011D	1
Trauma	10	S42122A	2
Trauma	10	S3502XA	1
Trauma	10	S82822A	2
Trauma	10	S41011A	6
Trauma	10	S066X2A	5
Trauma	10	S85181A	3
Trauma	10	S82832C	2
Trauma	10	T24332D	1
Trauma	10	S27301A	1
Trauma	10	T3141	1
Trauma	10	S32392D	1
Trauma	10	T2652XA	1

© 2021 Pelletier et al. *JAMA Network Open.*

Diagnosis	ICD Version	ICD Code	Number of Encounters
Trauma	10	S2692XA	3
Trauma	10	S2502XA	3
Trauma	10	S98311A	5
Trauma	10	S82812A	2
Trauma	10	T22022A	2
Trauma	10	S72362B	1
Trauma	10	S82201H	1
Trauma	10	S7222XP	2
Trauma	10	S52222C	1
Trauma	10	S36508A	3
Trauma	10	S45811A	1
Trauma	10	S45191A	3
Trauma	10	T22411A	1
Trauma	10	S75112A	3
Trauma	10	S98921A	4
Trauma	10	S62201B	1
Trauma	10	S31021A	1
Trauma	10	S13160A	2
Trauma	10	S31615A	3
Trauma	10	S72332C	3

© 2021 Pelletier et al. *JAMA Network Open.*

Diagnosis	ICD Version	ICD Code	Number of Encounters
Trauma	10	S45011A	2
Trauma	10	S71102S	1
Trauma	10	S70921A	1
Trauma	10	S6291XA	1
Trauma	10	S52351C	3
Trauma	10	S72441K	1
Trauma	10	S52371C	1
Trauma	10	S88111S	1
Trauma	10	S89312A	6
Trauma	10	S82251D	1
Trauma	10	S52102A	7
Trauma	10	S01122D	1
Trauma	10	S9411XA	1
Trauma	10	S52331C	2
Trauma	10	S85512A	1
Trauma	10	S71132D	1
Trauma	10	S72354G	1
Trauma	10	S82309A	5
Trauma	10	S36239D	1
Trauma	10	S22029A	8

© 2021 Pelletier et al. *JAMA Network Open.*

Diagnosis	ICD Version	ICD Code	Number of Encounters
Trauma	10	S52101C	1
Trauma	10	S24152S	3
Trauma	10	S52024A	4
Trauma	10	S72051B	2
Trauma	10	S82224K	1
Trauma	10	S79012K	1
Trauma	10	S72002P	4
Trauma	10	S36201A	2
Trauma	10	S0097XA	1
Trauma	10	S92154B	1
Trauma	10	S82401C	1
Trauma	10	S76009A	1
Trauma	10	S59292A	8
Trauma	10	S27818S	1
Trauma	10	S0510XA	1
Trauma	10	S0281XG	1
Trauma	10	S30860A	3
Trauma	10	S25499A	1
Trauma	10	S06895S	1
Trauma	10	S62300B	1

© 2021 Pelletier et al. *JAMA Network Open.*

Diagnosis	ICD Version	ICD Code	Number of Encounters
Trauma	10	S82421C	1
Trauma	10	S89091A	4
Trauma	10	S01102A	1
Trauma	10	S92321B	5
Trauma	10	S82142D	1
Trauma	10	S21109A	2
Trauma	10	S1111XA	3
Trauma	10	S7222XD	3
Trauma	10	S0031XA	4
Trauma	10	T17510A	2
Trauma	10	S322XXA	2
Trauma	10	S42345A	4
Trauma	10	S2759XA	1
Trauma	10	S62525A	1
Trauma	10	S42392A	7
Trauma	10	S0193XS	1
Trauma	10	T17818A	2
Trauma	10	T24791A	1
Trauma	10	S60371A	1
Trauma	10	S62666B	2

© 2021 Pelletier et al. *JAMA Network Open.*

Diagnosis	ICD Version	ICD Code	Number of Encounters
Trauma	10	S62391B	1
Trauma	10	S098XXS	1
Trauma	10	S36429A	4
Trauma	10	S52391C	3
Trauma	10	S5291XJ	1
Trauma	10	S42324A	2
Trauma	10	S91211A	1
Trauma	10	S32452B	1
Trauma	10	S52691A	4
Trauma	10	S82042C	2
Trauma	10	S31104S	1
Trauma	10	S8401XA	3
Trauma	10	S58112A	2
Trauma	10	S62325B	2
Trauma	10	S82451C	1
Trauma	10	S80261A	2
Trauma	10	T24111A	1
Trauma	10	S82842C	5
Trauma	10	S42002B	1
Trauma	10	S72421D	1

© 2021 Pelletier et al. *JAMA Network Open.*

Diagnosis	ICD Version	ICD Code	Number of Encounters
Trauma	10	S40222A	1
Trauma	10	S11031A	2
Trauma	10	S91154A	1
Trauma	10	S2222XK	2
Trauma	10	S8254XA	5
Trauma	10	S62322G	1
Trauma	10	S15012A	1
Trauma	10	S42415K	1
Trauma	10	S92121A	2
Trauma	10	S82151B	4
Trauma	10	S24102S	3
Trauma	10	S62337A	1
Trauma	10	S79012P	1
Trauma	10	S72432C	2
Trauma	10	S72342E	1
Trauma	10	S62611D	1
Trauma	10	S3092XA	1
Trauma	10	S4992XA	4
Trauma	10	S52202K	1
Trauma	10	S26020A	1

© 2021 Pelletier et al. *JAMA Network Open.*

Diagnosis	ICD Version	ICD Code	Number of Encounters
Trauma	10	S53024A	1
Trauma	10	S82871B	2
Trauma	10	S72462C	1
Trauma	10	S0230XA	1
Trauma	10	S01421D	1
Trauma	10	S83206A	1
Trauma	10	S72142K	2
Trauma	10	S72092B	2
Trauma	10	S42012B	1
Trauma	10	S61235A	2
Trauma	10	S71001A	1
Trauma	10	S334XXA	2
Trauma	10	S92122A	3
Trauma	10	S62305K	1
Trauma	10	S21301A	2
Trauma	10	S1192XA	4
Trauma	10	S82201N	2
Trauma	10	S36029D	1
Trauma	10	S82842D	1
Trauma	10	S70219A	1

© 2021 Pelletier et al. *JAMA Network Open.*

Diagnosis	ICD Version	ICD Code	Number of Encounters
Trauma	10	S3282XD	4
Trauma	10	S8264XA	3
Trauma	10	S14124A	1
Trauma	10	S53194A	2
Trauma	10	S030XXD	1
Trauma	10	S21412A	1
Trauma	10	S62001K	3
Trauma	10	S0550XA	1
Trauma	10	S62603B	1
Trauma	10	S89202A	1
Trauma	10	S89122D	3
Trauma	10	S72401E	1
Trauma	10	S56425A	1
Trauma	10	S32316A	1
Trauma	10	S02831A	4
Trauma	10	S82302H	1
Trauma	10	S82832H	1
Trauma	10	S71031A	2
Trauma	10	S36500A	1
Trauma	10	S8251XG	1

© 2021 Pelletier et al. *JAMA Network Open.*

Diagnosis	ICD Version	ICD Code	Number of Encounters
Trauma	10	S88111A	3
Trauma	10	S14111A	2
Trauma	10	S82102D	1
Trauma	10	S81812D	3
Trauma	10	S21302A	7
Trauma	10	S83232A	2
Trauma	10	S39091A	6
Trauma	10	S32391D	1
Trauma	10	S36501A	2
Trauma	10	S8981XD	1
Trauma	10	S60011A	1
Trauma	10	S8981XA	4
Trauma	10	S62333B	3
Trauma	10	S72391D	1
Trauma	10	S49009A	1
Trauma	10	S2521XA	1
Trauma	10	S5292XC	1
Trauma	10	S128XXS	1
Trauma	10	S76311A	2
Trauma	10	T282XXA	1

© 2021 Pelletier et al. *JAMA Network Open.*

Diagnosis	ICD Version	ICD Code	Number of Encounters
Trauma	10	S02102B	1
Trauma	10	S92252A	2
Trauma	10	S14116A	5
Trauma	10	S14115D	2
Trauma	10	S14155S	1
Trauma	10	S42002D	1
Trauma	10	S6991XA	3
Trauma	10	S88111D	2
Trauma	10	S06309D	12
Trauma	10	S62624B	1
Trauma	10	S32130A	1
Trauma	10	S3339XA	1
Trauma	10	S72301G	2
Trauma	10	S14157D	2
Trauma	10	S92241A	1
Trauma	10	S80812S	2
Trauma	10	S52591P	1
Trauma	10	S42021D	1
Trauma	10	S42392K	1
Trauma	10	S1189XA	3

© 2021 Pelletier et al. *JAMA Network Open.*

Diagnosis	ICD Version	ICD Code	Number of Encounters
Trauma	10	S06300D	2
Trauma	10	S31630A	3
Trauma	10	S14157S	1
Trauma	10	S61542A	4
Trauma	10	S36031D	3
Trauma	10	S32301K	1
Trauma	10	S89301A	2
Trauma	10	S32451B	1
Trauma	10	S56821A	3
Trauma	10	S63044A	1
Trauma	10	S59101A	2
Trauma	10	S12590B	1
Trauma	10	S63695A	1
Trauma	10	S24113A	4
Trauma	10	S37061D	1
Trauma	10	S0083XD	1
Trauma	10	S0240DD	1
Trauma	10	S97122A	2
Trauma	10	S34122D	1
Trauma	10	S02402D	2

© 2021 Pelletier et al. *JAMA Network Open.*

Diagnosis	ICD Version	ICD Code	Number of Encounters
Trauma	10	S43422D	1
Trauma	10	S82101N	1
Trauma	10	S66222A	1
Trauma	10	S06319S	1
Trauma	10	S27893D	1
Trauma	10	S82301M	1
Trauma	10	S83522A	5
Trauma	10	S52572B	1
Trauma	10	S52092K	1
Trauma	10	S86019A	2
Trauma	10	S68722A	2
Trauma	10	S53135A	1
Trauma	10	S27339A	2
Trauma	10	S170XXA	4
Trauma	10	S42141A	4
Trauma	10	S68613A	5
Trauma	10	S90931D	1
Trauma	10	S86811A	1
Trauma	10	S92301A	2
Trauma	10	S96921A	1

© 2021 Pelletier et al. *JAMA Network Open.*

Diagnosis	ICD Version	ICD Code	Number of Encounters
Trauma	10	S31814D	1
Trauma	10	S82142C	2
Trauma	10	S0281XS	1
Trauma	10	S21442A	4
Trauma	10	S35512A	2
Trauma	10	S022XXG	1
Trauma	10	S83124A	3
Trauma	10	T23002D	1
Trauma	10	S36892D	1
Trauma	10	S52132B	1
Trauma	10	S92422G	2
Trauma	10	S01401A	1
Trauma	10	S61257A	2
Trauma	10	S31809D	1
Trauma	10	S52025A	6
Trauma	10	S1096XA	1
Trauma	10	S61002A	1
Trauma	10	S89001A	8
Trauma	10	S3091XA	1
Trauma	10	S92812B	3

© 2021 Pelletier et al. *JAMA Network Open.*

Diagnosis	ICD Version	ICD Code	Number of Encounters
Trauma	10	S91302S	2
Trauma	10	S50312D	1
Trauma	10	S31839A	1
Trauma	10	S76221A	2
Trauma	10	S72301S	1
Trauma	10	S58121A	3
Trauma	10	S11012A	1
Trauma	10	S61459A	2
Trauma	10	S32011B	1
Trauma	10	S01111D	1
Trauma	10	S68611A	4
Trauma	10	S52571B	1
Trauma	10	S6990XA	1
Trauma	10	S00252A	1
Trauma	10	S32001A	2
Trauma	10	T2100XA	1
Trauma	10	S52023A	4
Trauma	10	T23091A	2
Trauma	10	S7402XD	1
Trauma	10	S96122D	1

© 2021 Pelletier et al. *JAMA Network Open.*

Diagnosis	ICD Version	ICD Code	Number of Encounters
Trauma	10	S6292XB	1
Trauma	10	S20222A	1
Trauma	10	S41159A	1
Trauma	10	S82432B	1
Trauma	10	S62307B	1
Trauma	10	S71041A	1
Trauma	10	T24201D	1
Trauma	10	T3132	1
Trauma	10	S42334A	2
Trauma	10	S52001K	1
Trauma	10	S91011D	1
Trauma	10	S81022D	1
Trauma	10	S58122A	1
Trauma	10	S72141B	2
Trauma	10	T24101A	1
Trauma	10	S66326A	1
Trauma	10	S62521A	1
Trauma	10	S6422XA	1
Trauma	10	S22062A	2
Trauma	10	S31153A	1

© 2021 Pelletier et al. *JAMA Network Open.*

Diagnosis	ICD Version	ICD Code	Number of Encounters
Trauma	10	S4402XA	2
Trauma	10	T2145XA	1
Trauma	10	S62390B	1
Trauma	10	S7401XA	3
Trauma	10	S9402XA	1
Trauma	10	S27431A	3
Trauma	10	S21232A	2
Trauma	10	S90812A	3
Trauma	10	S61200A	1
Trauma	10	S36503A	1
Trauma	10	S98021A	2
Trauma	10	S42112B	4
Trauma	10	S15102A	1
Trauma	10	S61402D	2
Trauma	10	S62325A	2
Trauma	10	S62221A	2
Trauma	10	S66321D	1
Trauma	10	S31501A	1
Trauma	10	S62001A	1
Trauma	10	S62211A	1

© 2021 Pelletier et al. *JAMA Network Open.*

Diagnosis	ICD Version	ICD Code	Number of Encounters
Trauma	10	S62002A	1
Trauma	10	S52322G	1
Trauma	10	S12110G	1
Trauma	10	S9301XD	1
Trauma	10	S62327A	2
Trauma	10	S82141G	1
Trauma	10	S5702XA	2
Trauma	10	S022XXK	1
Trauma	10	S73102A	2
Trauma	10	S72012D	1
Trauma	10	S82391G	1
Trauma	10	S79012G	1
Trauma	10	S52352G	1
Trauma	10	S72021K	1
Trauma	10	S62603P	1
Trauma	10	S62667G	1
Trauma	10	S52502G	1
Trauma	10	S00201A	1
Trauma	10	S42435A	2
Trauma	10	S52301G	1

© 2021 Pelletier et al. *JAMA Network Open.*

Diagnosis	ICD Version	ICD Code	Number of Encounters
Trauma	10	S52514A	1
Trauma	10	S02641B	2
Trauma	10	S82392P	1
Trauma	10	S72012P	1
Trauma	10	S52502H	1
Trauma	10	S92901A	1
Trauma	10	S31829D	1
Trauma	10	S36241A	3
Trauma	10	S61314A	2
Trauma	10	S76311D	1
Trauma	10	S93521A	1
Trauma	10	S59191A	2
Trauma	10	S7001XD	1
Trauma	10	S66021A	1
Trauma	10	S00271A	2
Trauma	10	S7011XS	1
Trauma	10	S86919A	1
Trauma	10	S62611A	4
Trauma	10	S80869A	1
Trauma	10	S93431A	1

© 2021 Pelletier et al. *JAMA Network Open.*

Diagnosis	ICD Version	ICD Code	Number of Encounters
Trauma	10	S82842S	1
Trauma	10	S143XXS	3
Trauma	10	S64497A	1
Trauma	10	S61355A	1
Trauma	10	S52552G	1
Trauma	10	S42445A	1
Trauma	10	S56811A	1
Trauma	10	S00252D	1
Trauma	10	S648X2A	1
Trauma	10	S42455A	4
Trauma	10	S064X2A	2
Trauma	10	S81832S	1
Trauma	10	S62605B	2
Trauma	10	S59001A	1
Trauma	10	S0265XD	1
Trauma	10	S52272G	1
Trauma	10	S82891S	1
Trauma	10	S0261XD	1
Trauma	10	S06306A	1
Trauma	10	S72392S	1

© 2021 Pelletier et al. *JAMA Network Open.*

Diagnosis	ICD Version	ICD Code	Number of Encounters
Trauma	10	S82114A	1
Trauma	10	S92325A	1
Trauma	10	S42461K	2
Trauma	10	S83501A	1
Trauma	10	S62624A	2
Trauma	10	T17898S	1
Trauma	10	S00522A	2
Trauma	10	S82301D	2
Trauma	10	S61251S	1
Trauma	10	S51801D	1
Trauma	10	S7221XG	1
Trauma	10	S61316A	2
Trauma	10	S0262XB	3
Trauma	10	S728X1G	1
Trauma	10	S91132A	1
Trauma	10	S52271G	2
Trauma	10	S91152A	1
Trauma	10	S023XXG	1
Trauma	10	S90456A	1
Trauma	10	S52272K	3

© 2021 Pelletier et al. *JAMA Network Open.*

Diagnosis	ICD Version	ICD Code	Number of Encounters
Trauma	10	S82312G	1
Trauma	10	S5292XP	3
Trauma	10	S53104A	2
Trauma	10	S68124D	1
Trauma	10	S62397B	1
Trauma	10	S42441G	1
Trauma	10	S52271Q	1
Trauma	10	S2619XD	1
Trauma	10	S82831S	1
Trauma	10	S35299A	1
Trauma	10	S36039D	1
Trauma	10	S62307A	1
Trauma	10	S83105D	2
Trauma	10	S66891A	1
Trauma	10	S52132G	2
Trauma	10	S52591E	1
Trauma	10	S12601G	1
Trauma	10	S8252XG	1
Trauma	10	S42412G	3
Trauma	10	S52131G	1

© 2021 Pelletier et al. *JAMA Network Open.*

Diagnosis	ICD Version	ICD Code	Number of Encounters
Trauma	10	S79121G	1
Trauma	10	S42294A	3
Trauma	10	S8982XS	1
Trauma	10	S6431XA	1
Trauma	10	S22048G	1
Trauma	10	S0080XA	1
Trauma	10	S56392A	1
Trauma	10	S65112A	4
Trauma	10	S76019A	1
Trauma	10	S02651G	1
Trauma	10	S62602A	2
Trauma	10	S61243A	1
Trauma	10	T24302D	2
Trauma	10	S86911A	2
Trauma	10	S91114A	2
Trauma	10	S82252H	1
Trauma	10	S62292A	2
Trauma	10	S68119A	1
Trauma	10	S62601A	1
Trauma	10	S3993XA	1

© 2021 Pelletier et al. *JAMA Network Open.*

Diagnosis	ICD Version	ICD Code	Number of Encounters
Trauma	10	T2602XS	1
Trauma	10	S72141P	1
Trauma	10	S3982XS	1
Trauma	10	S61111A	1
Trauma	10	S0219XS	3
Trauma	10	S92101A	2
Trauma	10	S59219A	1
Trauma	10	S59142A	1
Trauma	10	S63064A	1
Trauma	10	S52501G	3
Trauma	10	S04011A	1
Trauma	10	S82852D	1
Trauma	10	S82402M	1
Trauma	10	S52122P	2
Trauma	10	S88112S	1
Trauma	10	S62502A	1
Trauma	10	S61216A	2
Trauma	10	S0280XS	1
Trauma	10	S4291XA	2
Trauma	10	S62231A	3

© 2021 Pelletier et al. *JAMA Network Open.*

Diagnosis	ICD Version	ICD Code	Number of Encounters
Trauma	10	S31831D	1
Trauma	10	S061X9S	1
Trauma	10	S90446A	1
Trauma	10	S42121A	3
Trauma	10	S01339A	1
Trauma	10	S62633A	1
Trauma	10	S67197A	3
Trauma	10	S36202D	1
Trauma	10	S96021D	1
Trauma	10	S67195A	3
Trauma	10	S52511P	1
Trauma	10	S4292XA	1
Trauma	10	S62021A	3
Trauma	10	S01151D	1
Trauma	10	S064X1S	1
Trauma	10	S0035XA	1
Trauma	10	S62323A	1
Trauma	10	S22028A	6
Trauma	10	S8252XP	1
Trauma	10	S32612K	1

© 2021 Pelletier et al. *JAMA Network Open.*

Diagnosis	ICD Version	ICD Code	Number of Encounters
Trauma	10	S61304A	1
Trauma	10	S42452K	3
Trauma	10	S72321G	1
Trauma	10	S02652K	1
Trauma	10	S61313A	3
Trauma	10	S61226A	1
Trauma	10	S12000S	1
Trauma	10	S79822S	1
Trauma	10	S80211S	1
Trauma	10	S8261XG	1
Trauma	10	S52209A	2
Trauma	10	S06309S	2
Trauma	10	S52352M	1
Trauma	10	S82232G	1
Trauma	10	S4992XD	1
Trauma	10	S02113B	2
Trauma	10	S62202A	1
Trauma	10	S066X3S	1
Trauma	10	S8991XA	8
Trauma	10	S92011A	2

© 2021 Pelletier et al. *JAMA Network Open.*

Diagnosis	ICD Version	ICD Code	Number of Encounters
Trauma	10	S32301B	2
Trauma	10	S98122S	1
Trauma	10	S72461C	2
Trauma	10	T189XXD	1
Trauma	10	S89031P	1
Trauma	10	S24151A	1
Trauma	10	S89221A	1
Trauma	10	S06343A	1
Trauma	10	S60571A	1
Trauma	10	S62303A	1
Trauma	10	S12190B	1
Trauma	10	S98011A	1
Trauma	10	S33101A	1
Trauma	10	S52092A	3
Trauma	10	S79122D	2
Trauma	10	S37049A	1
Trauma	10	S0591XA	1
Trauma	10	S32019B	1
Trauma	10	S72032S	1
Trauma	10	S0124XA	2

© 2021 Pelletier et al. *JAMA Network Open.*

Diagnosis	ICD Version	ICD Code	Number of Encounters
Trauma	10	S41002A	2
Trauma	10	S42462B	4
Trauma	10	S82032P	1
Trauma	10	S06324A	1
Trauma	10	S82231G	1
Trauma	10	S32424A	1
Trauma	10	S32424D	1
Trauma	10	S06302A	1
Trauma	10	S5422XD	2
Trauma	10	S32602A	3
Trauma	10	S12330A	2
Trauma	10	S81021D	1
Trauma	10	S52332C	1
Trauma	10	S53114A	2
Trauma	10	S62254A	1
Trauma	10	S41031A	6
Trauma	10	S21149A	1
Trauma	10	S31833A	1
Trauma	10	S32692A	3
Trauma	10	S72351G	1

© 2021 Pelletier et al. *JAMA Network Open.*

Diagnosis	ICD Version	ICD Code	Number of Encounters
Trauma	10	T24612A	2
Trauma	10	S90572A	1
Trauma	10	S72431E	1
Trauma	10	S92211D	1
Trauma	10	S42034B	1
Trauma	10	S71042A	2
Trauma	10	S32692B	1
Trauma	10	S06317A	1
Trauma	10	T2610XA	1
Trauma	10	S3210XB	2
Trauma	10	S72354B	2
Trauma	10	S62317B	1
Trauma	10	S21231A	2
Trauma	10	S82101C	3
Trauma	10	S21239A	1
Trauma	10	S62112A	1
Trauma	10	S92334A	1
Trauma	10	T2009XD	2
Trauma	10	S72333A	2
Trauma	10	S22071D	1

© 2021 Pelletier et al. *JAMA Network Open.*

Diagnosis	ICD Version	ICD Code	Number of Encounters
Trauma	10	S92501A	1
Trauma	10	S41032A	3
Trauma	10	S15192A	4
Trauma	10	S7224XK	1
Trauma	10	S82831C	4
Trauma	10	S32485A	1
Trauma	10	S32425B	1
Trauma	10	S24153S	1
Trauma	10	S34113D	2
Trauma	10	S02849A	1
Trauma	10	S52002G	1
Trauma	10	S06375D	1
Trauma	10	S63094A	1
Trauma	10	S92425A	1
Trauma	10	S73001S	2
Trauma	10	S7223XA	1
Trauma	10	S72402K	1
Trauma	10	S01502A	5
Trauma	10	S60413A	1
Trauma	10	S30813A	1

© 2021 Pelletier et al. *JAMA Network Open.*

Diagnosis	ICD Version	ICD Code	Number of Encounters
Trauma	10	S63250A	1
Trauma	10	S01451S	4
Trauma	10	S62111A	1
Trauma	10	S52325A	3
Trauma	10	S76001A	1
Trauma	10	S60861A	2
Trauma	10	S3699XD	1
Trauma	10	S32022D	2
Trauma	10	S34102D	1
Trauma	10	S01119A	4
Trauma	10	S065X4D	1
Trauma	10	S42341P	1
Trauma	10	S8291XA	1
Trauma	10	S96991A	1
Trauma	10	S06331D	1
Trauma	10	S24133A	1
Trauma	10	S22082D	1
Trauma	10	S72354D	1
Trauma	10	S028XXD	1
Trauma	10	S72391S	1

© 2021 Pelletier et al. *JAMA Network Open.*

Diagnosis	ICD Version	ICD Code	Number of Encounters
Trauma	10	S14102S	2
Trauma	10	S32492D	1
Trauma	10	S12200D	1
Trauma	10	S32302D	1
Trauma	10	S0262XD	1
Trauma	10	S069X1S	3
Trauma	10	S3282XS	1
Trauma	10	S5291XG	1
Trauma	10	S24103S	4
Trauma	10	S14109S	1
Trauma	10	S32039D	2
Trauma	10	S82201G	2
Trauma	10	S52224B	2
Trauma	10	S32011D	3
Trauma	10	S12330D	1
Trauma	10	S82291D	1
Trauma	10	S025XXD	1
Trauma	10	S32313D	1
Trauma	10	S06370D	1
Trauma	10	S06341D	1

© 2021 Pelletier et al. *JAMA Network Open.*

Diagnosis	ICD Version	ICD Code	Number of Encounters
Trauma	10	S52561D	1
Trauma	10	S0180XD	1
Trauma	10	S52531B	1
Trauma	10	S14101D	2
Trauma	10	S12590D	2
Trauma	10	S92424B	5
Trauma	10	S0240FD	1
Trauma	10	S60441A	1
Trauma	10	S21439A	1
Trauma	10	S60474A	1
Trauma	10	S14129A	4
Trauma	10	S65812A	1
Trauma	10	T2007XD	1
Trauma	10	S88112D	1
Trauma	10	S52621A	2
Trauma	10	S98912A	1
Trauma	10	S62235A	2
Trauma	10	S82191G	1
Trauma	10	S63260A	2
Trauma	10	S82254A	3

© 2021 Pelletier et al. *JAMA Network Open.*

Diagnosis	ICD Version	ICD Code	Number of Encounters
Trauma	10	T3183	1
Trauma	10	S92523A	1
Trauma	10	S91135A	2
Trauma	10	S52209B	1
Trauma	10	S2231XD	3
Trauma	10	T3140	1
Trauma	10	S82402F	1
Trauma	10	S066X2D	1
Trauma	10	S72332P	1
Trauma	10	S0531XD	1
Trauma	10	S32692K	1
Trauma	10	S61451D	2
Trauma	10	S52132P	1
Trauma	10	S92012A	1
Trauma	10	S82301H	1
Trauma	10	S93314D	1
Trauma	10	S0193XD	2
Trauma	10	S43001A	1
Trauma	10	S92302A	1
Trauma	10	S72361D	1

© 2021 Pelletier et al. *JAMA Network Open.*

Diagnosis	ICD Version	ICD Code	Number of Encounters
Trauma	10	S52562B	1
Trauma	10	S50852A	2
Trauma	10	S79911A	4
Trauma	10	S52091A	3
Trauma	10	S42432P	1
Trauma	10	S78111D	1
Trauma	10	S728X1F	1
Trauma	10	S022XXS	4
Trauma	10	S13141D	1
Trauma	10	S79132S	1
Trauma	10	S14107S	1
Trauma	10	S82251P	1
Trauma	10	S0037XA	1
Trauma	10	S34121D	3
Trauma	10	S06829D	1
Trauma	10	S0211AD	1
Trauma	10	S72309K	1
Trauma	10	S72042G	1
Trauma	10	S06820S	1
Trauma	10	S32402K	1

© 2021 Pelletier et al. *JAMA Network Open.*

Diagnosis	ICD Version	ICD Code	Number of Encounters
Trauma	10	S14105D	3
Trauma	10	S12400D	1
Trauma	10	S59211A	1
Trauma	10	S82302M	3
Trauma	10	T2132XD	2
Trauma	10	S52302P	1
Trauma	10	S82034B	1
Trauma	10	S14114D	2
Trauma	10	S42452P	2
Trauma	10	S82131A	1
Trauma	10	S062X4D	1
Trauma	10	S14153S	1
Trauma	10	S24159D	2
Trauma	10	S3219XK	1
Trauma	10	S34119D	1
Trauma	10	S56911A	1
Trauma	10	S81809A	1
Trauma	10	S82111B	2
Trauma	10	S84802A	1
Trauma	10	S92902A	1

© 2021 Pelletier et al. *JAMA Network Open.*

Diagnosis	ICD Version	ICD Code	Number of Encounters
Trauma	10	S92512A	3
Trauma	10	S21431A	2
Trauma	10	S066X5S	1
Trauma	10	S062X3D	3
Trauma	10	S061X4A	1
Trauma	10	S3092XD	1
Trauma	10	S06898D	1
Trauma	10	S78111A	3
Trauma	10	S93432A	1
Trauma	10	S86821D	1
Trauma	10	S8292XA	1
Trauma	10	S948X1A	1
Trauma	10	S62502B	1
Trauma	10	S83004D	1
Trauma	10	S52322C	1
Trauma	10	S92042A	2
Trauma	10	S92312P	1
Trauma	10	S89122K	1
Trauma	10	S0211GD	2
Trauma	10	S42454A	1

© 2021 Pelletier et al. *JAMA Network Open.*

Diagnosis	ICD Version	ICD Code	Number of Encounters
Trauma	10	S75009A	1
Trauma	10	S63418A	1
Trauma	10	S0090XA	1
Trauma	10	S82499D	1
Trauma	10	S62661B	1
Trauma	10	S76912A	2
Trauma	10	S32059B	1
Trauma	10	S6981XA	1
Trauma	10	S728X1C	1
Trauma	10	S82221P	1
Trauma	10	S32039K	1
Trauma	10	S43224D	1
Trauma	10	S32481A	1
Trauma	10	S72321S	1
Trauma	10	S73191S	1
Trauma	10	S91111D	1
Trauma	10	S52291A	4
Trauma	10	S72141K	2
Trauma	10	S72415A	2
Trauma	10	S40871A	1

© 2021 Pelletier et al. *JAMA Network Open.*

Diagnosis	ICD Version	ICD Code	Number of Encounters
Trauma	10	S82252J	1
Trauma	10	S70369A	1
Trauma	10	S82191C	3
Trauma	10	S89141G	1
Trauma	10	S82222G	1
Trauma	10	S52609B	1
Trauma	10	S52321P	1
Trauma	10	S52502S	1
Trauma	10	S42191A	2
Trauma	10	T17900D	1
Trauma	10	S51842D	1
Trauma	10	S36032D	2
Trauma	10	S61319D	1
Trauma	10	S8410XA	2
Trauma	10	S13111D	2
Trauma	10	S62102P	1
Trauma	10	S42293A	1
Trauma	10	T2126XS	1
Trauma	10	S86312A	1
Trauma	10	S0219XK	1

© 2021 Pelletier et al. *JAMA Network Open.*

Diagnosis	ICD Version	ICD Code	Number of Encounters
Trauma	10	S82841K	1
Trauma	10	S59131A	1
Trauma	10	S72124A	3
Trauma	10	S80842A	1
Trauma	10	S60022A	1
Trauma	10	S72342D	3
Trauma	10	S38221A	1
Trauma	10	S72342G	1
Trauma	10	S82875B	1
Trauma	10	S92322K	1
Trauma	10	S4991XA	3
Trauma	10	S72491S	1
Trauma	10	S89102D	1
Trauma	10	S82152D	1
Trauma	10	S0572XA	1
Trauma	10	S3663XD	2
Trauma	10	S27899A	1
Trauma	10	S72354H	1
Trauma	10	S32602K	1
Trauma	10	T22331S	1

© 2021 Pelletier et al. *JAMA Network Open.*

Diagnosis	ICD Version	ICD Code	Number of Encounters
Trauma	10	S0572XD	1
Trauma	10	T2662XA	1
Trauma	10	S90464A	1
Trauma	10	S21432A	1
Trauma	10	S2753XA	2
Trauma	10	S0125XS	1
Trauma	10	S0125XD	1
Trauma	10	S8782XD	1
Trauma	10	S62014A	1
Trauma	10	S52121B	2
Trauma	10	S15002A	1
Trauma	10	S70312A	3
Trauma	10	S92521A	1
Trauma	10	S13120S	1
Trauma	10	S68123D	1
Trauma	10	S82301P	1
Trauma	10	S0219XG	2
Trauma	10	S63651A	2
Trauma	10	S86901A	1
Trauma	10	S35491A	1

© 2021 Pelletier et al. *JAMA Network Open.*

Diagnosis	ICD Version	ICD Code	Number of Encounters
Trauma	10	S82234B	1
Trauma	10	S38231A	1
Trauma	10	S76321A	1
Trauma	10	S2242XB	2
Trauma	10	S62390A	1
Trauma	10	S72301P	4
Trauma	10	S72332D	1
Trauma	10	S86301A	1
Trauma	10	S61519A	2
Trauma	10	S0185XS	1
Trauma	10	S5411XA	2
Trauma	10	S36020A	2
Trauma	10	S60811A	1
Trauma	10	S01141A	1
Trauma	10	S52034A	1
Trauma	10	S60842A	1
Trauma	10	S27392A	2
Trauma	10	S39093A	1
Trauma	10	S82872C	3
Trauma	10	S82031B	1

© 2021 Pelletier et al. *JAMA Network Open.*

Diagnosis	ICD Version	ICD Code	Number of Encounters
Trauma	10	S72321K	1
Trauma	10	S72321D	2
Trauma	10	S52265B	1
Trauma	10	S82001C	1
Trauma	10	S233XXA	1
Trauma	10	S61221A	1
Trauma	10	S72462K	1
Trauma	10	S82434A	1
Trauma	10	S62318B	1
Trauma	10	S42332B	1
Trauma	10	S82252E	1
Trauma	10	S36242A	2
Trauma	10	S32021S	1
Trauma	10	S72145B	1
Trauma	10	S81042S	1
Trauma	10	S0442XA	2
Trauma	10	S32139A	1
Trauma	10	S30873A	1
Trauma	10	S29022A	2
Trauma	10	S42451G	1

© 2021 Pelletier et al. *JAMA Network Open.*

Diagnosis	ICD Version	ICD Code	Number of Encounters
Trauma	10	S52261B	1
Trauma	10	T17518A	1
Trauma	10	S83261A	1
Trauma	10	S62122B	1
Trauma	10	S62357B	1
Trauma	10	S93115A	1
Trauma	10	S52392C	1
Trauma	10	T25121A	2
Trauma	10	S82261N	1
Trauma	10	S82262S	1
Trauma	10	S62397A	1
Trauma	10	S27311A	1
Trauma	10	T24512A	1
Trauma	10	S35228A	1
Trauma	10	S46392A	1
Trauma	10	S59111A	1
Trauma	10	S85012A	3
Trauma	10	T25092A	2
Trauma	10	S64493A	2
Trauma	10	S5332XA	1

© 2021 Pelletier et al. *JAMA Network Open.*

Diagnosis	ICD Version	ICD Code	Number of Encounters
Trauma	10	S72322K	2
Trauma	10	S72492M	1
Trauma	10	S62642A	1
Trauma	10	T24032A	1
Trauma	10	S72132B	1
Trauma	10	S72052G	1
Trauma	10	S38222A	4
Trauma	10	S72445A	2
Trauma	10	S62142A	1
Trauma	10	S52025C	1
Trauma	10	S62141A	1
Trauma	10	S62638B	2
Trauma	10	S12650A	1
Trauma	10	S68512A	1
Trauma	10	S59242A	5
Trauma	10	S2500XA	1
Trauma	10	S158XXA	1
Trauma	10	S62242B	1
Trauma	10	S0240CK	1
Trauma	10	S0521XD	2

© 2021 Pelletier et al. *JAMA Network Open.*

Diagnosis	ICD Version	ICD Code	Number of Encounters
Trauma	10	S31823A	2
Trauma	10	S68616A	3
Trauma	10	S3131XS	1
Trauma	10	S62320B	2
Trauma	10	S06829A	1
Trauma	10	S92492B	2
Trauma	10	S01351S	1
Trauma	10	S14113A	1
Trauma	10	S42131A	1
Trauma	10	S34122A	1
Trauma	10	S3216XA	1
Trauma	10	S70311D	1
Trauma	10	S82461B	1
Trauma	10	S32471A	1
Trauma	10	S1012XA	1
Trauma	10	S61151A	1
Trauma	10	T2079XA	1
Trauma	10	S59292P	1
Trauma	10	T22192A	1
Trauma	10	S68618A	1

© 2021 Pelletier et al. *JAMA Network Open.*

Diagnosis	ICD Version	ICD Code	Number of Encounters
Trauma	10	S12190D	2
Trauma	10	T2035XD	3
Trauma	10	S9702XA	2
Trauma	10	S52121P	1
Trauma	10	T24102A	2
Trauma	10	S42465A	1
Trauma	10	S52242B	1
Trauma	10	T22362A	1
Trauma	10	S0003XS	1
Trauma	10	S06365D	1
Trauma	10	S52111A	1
Trauma	10	S3282XB	1
Trauma	10	T24731A	1
Trauma	10	T2069XA	1
Trauma	10	S1087XA	1
Trauma	10	S62101A	1
Trauma	10	S60522A	1
Trauma	10	S83125A	2
Trauma	10	T20012A	1
Trauma	10	S89149A	1

© 2021 Pelletier et al. *JAMA Network Open.*

Diagnosis	ICD Version	ICD Code	Number of Encounters
Trauma	10	S42443A	1
Trauma	10	S92402D	1
Trauma	10	S91301S	1
Trauma	10	T22131A	1
Trauma	10	S82223A	1
Trauma	10	T23652A	2
Trauma	10	T23071A	2
Trauma	10	S82235B	1
Trauma	10	S52279A	1
Trauma	10	T24412A	1
Trauma	10	S42494A	1
Trauma	10	S52311A	1
Trauma	10	T2052XA	1
Trauma	10	S79192D	1
Trauma	10	S78112S	1
Trauma	10	S82852S	1
Trauma	10	S83289A	1
Trauma	10	S82892C	1
Trauma	10	S7221XS	1
Trauma	10	S92511A	2

© 2021 Pelletier et al. *JAMA Network Open.*

Diagnosis	ICD Version	ICD Code	Number of Encounters
Trauma	10	S24109S	1
Trauma	10	S31502A	1
Trauma	10	S15001A	1
Trauma	10	S42032K	1
Trauma	10	S14104S	3
Trauma	10	S82452C	1
Trauma	10	S7291XB	3
Trauma	10	S06339S	1
Trauma	10	S85112A	1
Trauma	10	S21329A	1
Trauma	10	S02641K	1
Trauma	10	S82035B	1
Trauma	10	S01352D	1
Trauma	10	S06373A	1
Trauma	10	S85091D	1
Trauma	10	S14109D	5
Trauma	10	S26021A	1
Trauma	10	S82391K	1
Trauma	10	S42272A	1
Trauma	10	T2142XA	1

© 2021 Pelletier et al. *JAMA Network Open.*

Diagnosis	ICD Version	ICD Code	Number of Encounters
Trauma	10	S71032A	1
Trauma	10	S72102K	1
Trauma	10	S32030K	1
Trauma	10	S43204D	1
Trauma	10	S1125XA	1
Trauma	10	S52601E	1
Trauma	10	S32499B	1
Trauma	10	S37031D	1
Trauma	10	S68712A	1
Trauma	10	S34105A	2
Trauma	10	S3730XD	1
Trauma	10	S14129S	2
Trauma	10	S32048D	1
Trauma	10	S069X5S	1
Trauma	10	S24159A	1
Trauma	10	S62300A	2
Trauma	10	S52025B	1
Trauma	10	S52135A	3
Trauma	10	S36030D	2
Trauma	10	S61310A	1

© 2021 Pelletier et al. *JAMA Network Open.*

Diagnosis	ICD Version	ICD Code	Number of Encounters
Trauma	10	S52302D	1
Trauma	10	S14159D	1
Trauma	10	S9002XA	2
Trauma	10	S52271K	1
Trauma	10	S140XXD	2
Trauma	10	S79141D	1
Trauma	10	S01341A	1
Trauma	10	S79822D	1
Trauma	10	S30851A	1
Trauma	10	S61217D	1
Trauma	10	S65510A	1
Trauma	10	S36021D	1
Trauma	10	S82831P	1
Trauma	10	S72342K	1
Trauma	10	S91022D	1
Trauma	10	S79131D	1
Trauma	10	S42024D	1
Trauma	10	S8992XD	1
Trauma	10	S91141A	1
Trauma	10	S62664G	1

© 2021 Pelletier et al. *JAMA Network Open.*

Diagnosis	ICD Version	ICD Code	Number of Encounters
Trauma	10	S59002A	2
Trauma	10	S30816A	1
Trauma	10	S7492XA	3
Trauma	10	S68623D	1
Trauma	10	S76111D	1
Trauma	10	S52272H	1
Trauma	10	S42025D	1
Trauma	10	S14123D	1
Trauma	10	S52352P	1
Trauma	10	S20461A	1
Trauma	10	S61222A	1
Trauma	10	S32000A	1
Trauma	10	S92322D	1
Trauma	10	S11014A	1
Trauma	10	S66222D	1
Trauma	10	S62323B	3
Trauma	10	S61315A	1
Trauma	10	S89142K	1
Trauma	10	S92354A	1
Trauma	10	S72401S	1

© 2021 Pelletier et al. *JAMA Network Open.*

Diagnosis	ICD Version	ICD Code	Number of Encounters
Trauma	10	S61351A	1
Trauma	10	S32691D	1
Trauma	10	S62022K	1
Trauma	10	T281XXS	1
Trauma	10	S79102K	2
Trauma	10	S79122P	2
Trauma	10	S42021K	1
Trauma	10	S55811A	1
Trauma	10	S41151D	1
Trauma	10	S06376A	1
Trauma	10	S88122D	1
Trauma	10	S41021A	1
Trauma	10	S40819A	1
Trauma	10	S42231A	1
Trauma	10	S37819A	1
Trauma	10	S72355M	1
Trauma	10	S062X2A	1
Trauma	10	S344XXA	1
Trauma	10	S72461B	1
Trauma	10	S46292A	1

© 2021 Pelletier et al. *JAMA Network Open.*

Diagnosis	ICD Version	ICD Code	Number of Encounters
Trauma	10	S02129A	1
Trauma	10	S46812A	1
Trauma	10	S82435A	1
Trauma	10	S82224B	1
Trauma	10	S6391XA	1
Trauma	10	S72301K	4
Trauma	10	S638X1A	1
Trauma	10	S79922A	2
Trauma	10	S82141E	1
Trauma	10	S8492XA	2
Trauma	10	S42451D	1
Trauma	10	S62331A	1
Trauma	10	S12390D	1
Trauma	10	S199XXD	1
Trauma	10	S52201G	1
Trauma	10	S9781XD	1
Trauma	10	S09391A	1
Trauma	10	S56124A	1
Trauma	10	S72142D	2
Trauma	10	T24132A	1

© 2021 Pelletier et al. *JAMA Network Open.*

Diagnosis	ICD Version	ICD Code	Number of Encounters
Trauma	10	S329XXD	1
Trauma	10	S358X1A	1
Trauma	10	S06361D	1
Trauma	10	S72061A	1
Trauma	10	S66422A	1
Trauma	10	S27898A	1
Trauma	10	S31801A	2
Trauma	10	S82853A	1
Trauma	10	S68627A	4
Trauma	10	S0992XA	2
Trauma	10	S15301A	1
Trauma	10	S76992A	1
Trauma	10	S06332A	1
Trauma	10	S39001A	2
Trauma	10	S85311A	1
Trauma	10	S2241XB	1
Trauma	10	S73014S	1
Trauma	10	S32599D	1
Trauma	10	S56892A	1
Trauma	10	S20412A	2

© 2021 Pelletier et al. *JAMA Network Open.*

Diagnosis	ICD Version	ICD Code	Number of Encounters
Trauma	10	S52251C	1
Trauma	10	S58921D	1
Trauma	10	S2222XD	1
Trauma	10	S62630D	1
Trauma	10	S06330D	1
Trauma	10	S76802A	1
Trauma	10	S46121A	1
Trauma	10	S72302G	1
Trauma	10	T2000XD	3
Trauma	10	S62600B	2
Trauma	10	S32423A	1
Trauma	10	S72351K	1
Trauma	10	S32008A	1
Trauma	10	S62235B	1
Trauma	10	S4421XA	3
Trauma	10	S76302A	1
Trauma	10	S43431A	1
Trauma	10	S22012A	1
Trauma	10	S36419A	1
Trauma	10	S12530A	2

© 2021 Pelletier et al. *JAMA Network Open.*

Diagnosis	ICD Version	ICD Code	Number of Encounters
Trauma	10	S13170D	1
Trauma	10	S72352P	1
Trauma	10	S32309A	1
Trauma	10	S24111A	2
Trauma	10	S31649A	1
Trauma	10	S22048D	1
Trauma	10	S82241C	1
Trauma	10	S36200A	2
Trauma	10	S62600A	1
Trauma	10	S52041B	1
Trauma	10	S0036XS	1
Trauma	10	S36231D	1
Trauma	10	S89112A	3
Trauma	10	S8253XA	1
Trauma	10	S82292N	1
Trauma	10	S96111A	1
Trauma	10	S59911A	1
Trauma	10	S86311A	1
Trauma	10	S82309S	1
Trauma	10	S72001P	1

© 2021 Pelletier et al. *JAMA Network Open.*

Diagnosis	ICD Version	ICD Code	Number of Encounters
Trauma	10	S66122A	3
Trauma	10	S72035K	1
Trauma	10	S62669A	1
Trauma	10	S82009A	1
Trauma	10	S42495B	1
Trauma	10	S82871K	1
Trauma	10	S91144A	1
Trauma	10	S7221XP	1
Trauma	10	S82899A	1
Trauma	10	S7222XK	1
Trauma	10	S37059A	1
Trauma	10	S68511A	5
Trauma	10	S92192A	1
Trauma	10	S27421A	1
Trauma	10	S52001P	1
Trauma	10	T22132A	1
Trauma	10	T3342XA	1
Trauma	10	S79011P	1
Trauma	10	S90451A	1
Trauma	10	S21129A	1

© 2021 Pelletier et al. *JAMA Network Open.*

Diagnosis	ICD Version	ICD Code	Number of Encounters
Trauma	10	S11029A	1
Trauma	10	S42492P	1
Trauma	10	S52611B	2
Trauma	10	T17918D	1
Trauma	10	S62306A	2
Trauma	10	S728X2K	1
Trauma	10	S01332A	1
Trauma	10	S62202B	2
Trauma	10	S32018K	1
Trauma	10	S43081A	1
Trauma	10	S9422XA	1
Trauma	10	S86321A	1
Trauma	10	S36249A	1
Trauma	10	S64491A	2
Trauma	10	S82102N	1
Trauma	10	S83281S	1
Trauma	10	S85202A	1
Trauma	10	S71012A	2
Trauma	10	S82113A	1
Trauma	10	T22211D	1

© 2021 Pelletier et al. *JAMA Network Open.*

Diagnosis	ICD Version	ICD Code	Number of Encounters
Trauma	10	S7291XP	2
Trauma	10	S32311B	1
Trauma	10	T17318A	1
Trauma	10	S21401A	1
Trauma	10	S42215A	1
Trauma	10	S42471B	1
Trauma	10	S42018A	1
Trauma	10	T23161A	1
Trauma	10	S59092A	2
Trauma	10	S3712XA	1
Trauma	10	S01541D	1
Trauma	10	S79011S	1
Trauma	10	S5491XA	1
Trauma	10	S72342B	1
Trauma	10	S61234A	1
Trauma	10	S63692A	1
Trauma	10	S169XXA	1
Trauma	10	S82152P	1
Trauma	10	S82192N	1
Trauma	10	S72131A	1

© 2021 Pelletier et al. *JAMA Network Open.*

Diagnosis	ICD Version	ICD Code	Number of Encounters
Trauma	10	S82262N	1
Trauma	10	S3219XD	1
Trauma	10	S52602P	1
Trauma	10	S42401P	1
Trauma	10	S7721XA	1
Trauma	10	S72451N	1
Trauma	10	S72362D	1
Trauma	10	S45891A	1
Trauma	10	S83282D	1
Trauma	10	T23092A	1
Trauma	10	S82132C	1
Trauma	10	S92201D	1
Trauma	10	S14129D	1
Trauma	10	S2501XA	1
Trauma	10	S02632B	1
Trauma	10	T25712A	1
Trauma	10	S31602A	1
Trauma	10	S34104A	1
Trauma	10	S82252D	1
Trauma	10	S82302J	1

© 2021 Pelletier et al. *JAMA Network Open.*

Diagnosis	ICD Version	ICD Code	Number of Encounters
Trauma	10	S72361C	1
Trauma	10	T22261A	1
Trauma	10	T23072A	1
Trauma	10	S37091D	1
Trauma	10	S51031A	1
Trauma	10	S72322G	1
Trauma	10	S36499D	1
Trauma	10	S27818D	1
Trauma	10	S82242N	1
Trauma	10	T2037XD	1
Trauma	10	T24021A	1
Trauma	10	T22042A	1
Trauma	10	S14151D	1
Trauma	10	T2006XD	1
Trauma	10	S43034A	1
Trauma	10	S72032K	1
Trauma	10	T2100XD	1
Trauma	10	S11024A	1
Trauma	10	S2759XD	1
Trauma	10	S96892A	1

© 2021 Pelletier et al. *JAMA Network Open.*

Diagnosis	ICD Version	ICD Code	Number of Encounters
Trauma	10	T25391D	1
Trauma	10	S15092A	1
Trauma	10	S72052E	1
Trauma	10	S85801A	1
Trauma	10	T24091D	1
Trauma	10	S98312A	1
Trauma	10	S15122A	2
Trauma	10	S20411A	2
Trauma	10	T25011A	1
Trauma	10	S62320A	1
Trauma	10	S72302N	1
Trauma	10	S32041D	1
Trauma	10	T24112A	1
Trauma	10	T3166	1
Trauma	10	T24022A	1
Trauma	10	S82301K	1
Trauma	10	S32810K	1
Trauma	10	S72091G	1
Trauma	10	S04891A	1
Trauma	10	S7291XE	1

© 2021 Pelletier et al. *JAMA Network Open.*

Diagnosis	ICD Version	ICD Code	Number of Encounters
Trauma	10	S61213S	1
Trauma	10	S3143XA	1
Trauma	10	S34111D	2
Trauma	10	S32461D	1
Trauma	10	S82831G	1
Trauma	10	S46912A	1
Trauma	10	S8990XA	2
Trauma	10	S82101P	1
Trauma	10	T2102XS	1
Trauma	10	S76892A	1
Trauma	10	S82102C	1
Trauma	10	S02601S	1
Trauma	10	S51041A	2
Trauma	10	S82014B	1
Trauma	10	S244XXA	1
Trauma	10	S92051B	1
Trauma	10	S028XXS	1
Trauma	10	S72391K	1
Trauma	10	S79112D	1
Trauma	10	S00501A	1

© 2021 Pelletier et al. *JAMA Network Open.*

Diagnosis	ICD Version	ICD Code	Number of Encounters
Trauma	10	S32592K	1
Trauma	10	S83102A	1
Trauma	10	S27491A	1
Trauma	10	S12350A	1
Trauma	10	S34123D	1
Trauma	10	S72435B	1
Trauma	10	S22088D	1
Trauma	10	T2039XD	1
Trauma	10	S52225A	1
Trauma	10	S22051D	1
Trauma	10	S178XXA	1
Trauma	10	S95001A	1
Trauma	10	S66901A	1
Trauma	10	S68011S	1
Trauma	10	S26022A	1
Trauma	10	S35492A	1
Trauma	10	S48112D	1
Trauma	10	S22060D	1
Trauma	10	S12111K	1
Trauma	10	S62312A	1

© 2021 Pelletier et al. *JAMA Network Open.*

Diagnosis	ICD Version	ICD Code	Number of Encounters
Trauma	10	S36250A	1
Trauma	10	S728X2B	1
Trauma	10	S83207A	1
Trauma	10	T24009A	1
Trauma	10	S62394A	1
Trauma	10	T2017XA	1
Trauma	10	S72401H	1
Trauma	10	S83115A	2
Trauma	10	S82465A	1
Trauma	10	T283XXA	1
Trauma	10	S83251D	1
Trauma	10	S0085XA	1
Trauma	10	T2002XA	1
Trauma	10	S72472D	1
Trauma	10	S63012A	1
Trauma	10	S53115A	1
Trauma	10	S06362A	1
Trauma	10	S62329A	1
Trauma	10	S68615A	1
Trauma	10	S53033A	1

© 2021 Pelletier et al. *JAMA Network Open.*

Diagnosis	ICD Version	ICD Code	Number of Encounters
Trauma	10	T2116XA	1
Trauma	10	S67192S	1
Trauma	10	S60131A	1
Trauma	10	T2013XA	1
Trauma	10	T189XXS	1
Trauma	10	S92315A	1
Trauma	10	S27819A	1
Trauma	10	S7222XC	1
Trauma	10	S82221K	1
Trauma	10	S82302K	1
Trauma	10	S15191A	2
Trauma	10	S7292XC	1
Trauma	10	S72432D	1
Trauma	10	S471XXA	1
Trauma	10	S14114A	2
Trauma	10	S72431C	1
Trauma	10	S32049B	1
Trauma	10	S66124A	1
Trauma	10	S63045A	1
Trauma	10	S82261B	1

© 2021 Pelletier et al. *JAMA Network Open.*

Diagnosis	ICD Version	ICD Code	Number of Encounters
Trauma	10	S72491C	2
Trauma	10	S82851B	1
Trauma	10	S82262A	5
Trauma	10	S52252A	1
Trauma	10	S0532XD	1
Trauma	10	S34124D	1
Trauma	10	S85151A	1
Trauma	10	S62022A	1
Trauma	10	S4411XA	1
Trauma	10	S62337B	1
Trauma	10	S42474A	1
Trauma	10	S67197S	1
Trauma	10	S52282C	1
Trauma	10	S3790XA	1
Trauma	10	S96912A	1
Trauma	10	S82262P	1
Trauma	10	S32311K	1
Trauma	10	S02101D	1
Trauma	10	S30844A	1
Trauma	10	S52021Q	1

© 2021 Pelletier et al. *JAMA Network Open.*

Diagnosis	ICD Version	ICD Code	Number of Encounters
Trauma	10	S42032P	1
Trauma	10	S066X6D	1
Trauma	10	S31132A	1
Trauma	10	S02122B	1
Trauma	10	S89021D	1
Trauma	10	S42111B	1
Trauma	10	S66113A	1
Trauma	10	T2104XD	1
Trauma	10	S36200D	1
Trauma	10	S83194A	1
Trauma	10	S85162A	1
Trauma	10	S52131B	1
Trauma	10	T23012A	1
Trauma	10	S51849A	1
Trauma	10	S61258A	1
Trauma	10	S53092A	1
Trauma	10	S89141D	1
Trauma	10	S0562XA	1
Trauma	10	S52232C	1
Trauma	10	S91311D	2

© 2021 Pelletier et al. *JAMA Network Open.*

Diagnosis	ICD Version	ICD Code	Number of Encounters
Trauma	10	S76902A	1
Trauma	10	S32432D	1
Trauma	10	S71052A	1
Trauma	10	S83222A	1
Trauma	10	S25312A	1
Trauma	10	S60412A	1
Trauma	10	T2174XA	1
Trauma	10	S61402S	1
Trauma	10	S82252N	2
Trauma	10	S8252XD	1
Trauma	10	S62032K	1
Trauma	10	T2027XD	1
Trauma	10	S63391S	1
Trauma	10	T17990S	1
Trauma	10	S32012D	1
Trauma	10	S37061S	1
Trauma	10	S12190G	1
Trauma	10	S12600S	1
Trauma	10	S1983XA	1
Trauma	10	S72091K	1

© 2021 Pelletier et al. *JAMA Network Open.*

Diagnosis	ICD Version	ICD Code	Number of Encounters
Trauma	10	S3510XA	1
Trauma	10	S82153B	1
Trauma	10	S02670B	1
Trauma	10	T23272D	1
Trauma	10	S92061G	1
Trauma	10	T2171XA	1
Trauma	10	T24732A	1
Trauma	10	T2005XS	1
Trauma	10	S31601A	1
Trauma	10	S75022D	1
Trauma	10	S52272C	1
Trauma	10	S134XXS	1
Trauma	10	S46822A	1
Trauma	10	S15021A	1
Trauma	10	T24721A	1
Trauma	10	S41042A	2
Trauma	10	T2156XA	1
Trauma	10	S72401J	1
Trauma	10	S65516A	1
Trauma	10	S85312A	1

© 2021 Pelletier et al. *JAMA Network Open.*

Diagnosis	ICD Version	ICD Code	Number of Encounters
Trauma	10	S52542A	1
Trauma	10	S82152C	1
Trauma	10	S0232XD	1
Trauma	10	S36112D	1
Trauma	10	S86802A	1
Trauma	10	S59042A	1
Trauma	10	S76292A	1
Trauma	10	S73044A	1
Trauma	10	S73045A	1
Trauma	10	S24112S	1
Trauma	10	S82821A	1
Trauma	10	S62606A	1
Trauma	10	S82432G	1
Trauma	10	S82242G	1
Trauma	10	S91341D	1
Trauma	10	S22088G	1
Trauma	10	S0266XG	1
Trauma	10	T25399S	1
Trauma	10	S72342P	1
Trauma	10	S27309A	1

© 2021 Pelletier et al. *JAMA Network Open.*

Diagnosis	ICD Version	ICD Code	Number of Encounters
Trauma	10	S32592B	1
Trauma	10	S52321C	1
Trauma	10	S82402G	1
Trauma	10	S0520XA	1
Trauma	10	S52181C	1
Trauma	10	S72142C	1
Trauma	10	T17810A	1
Trauma	10	S91119A	1
Trauma	10	S08119A	1
Trauma	10	T24001D	1
Trauma	10	S066X3D	1
Trauma	10	S91149A	1
Trauma	10	S32432B	1
Trauma	10	S88119D	1
Trauma	10	S92064A	1
Trauma	10	S42271A	1
Trauma	10	S02842A	3
Trauma	10	S51851D	1
Trauma	10	S82391D	1
Trauma	10	S0540XA	1

© 2021 Pelletier et al. *JAMA Network Open.*

Diagnosis	ICD Version	ICD Code	Number of Encounters
Trauma	10	S25322A	1
Trauma	10	S63014A	1
Trauma	10	S66303A	1
Trauma	10	S37893A	1
Trauma	10	S42135A	1
Trauma	10	S79149A	1
Trauma	10	S42411K	2
Trauma	10	S42022K	1
Trauma	10	S0919XA	1
Trauma	10	S9701XD	1
Trauma	10	S56121A	1
Trauma	10	S2241XD	2
Trauma	10	S82453A	1
Trauma	10	S93122A	1
Trauma	10	S32422B	1
Trauma	10	S89142D	1
Trauma	10	S36430D	1
Trauma	10	S3632XA	1
Trauma	10	S36420D	1
Trauma	10	S02109B	1

© 2021 Pelletier et al. *JAMA Network Open.*

Diagnosis	ICD Version	ICD Code	Number of Encounters
Trauma	10	S090XXD	1
Trauma	10	S37892A	1
Trauma	10	T22041A	2
Trauma	10	T3155	1
Trauma	10	S2224XA	1
Trauma	10	S02652D	1
Trauma	10	S06819D	1
Trauma	10	S62522G	1
Trauma	10	T25792A	1
Trauma	10	S62132A	1
Trauma	10	S92241B	1
Trauma	10	S61218A	1
Trauma	10	S02413S	1
Trauma	10	S06301D	1
Trauma	10	S02121B	1
Trauma	9	9351	2895
Trauma	9	80121	1569
Trauma	9	81241	10716
Trauma	9	8248	1935
Trauma	9	8901	184

© 2021 Pelletier et al. *JAMA Network Open.*

Diagnosis	ICD Version	ICD Code	Number of Encounters
Trauma	9	8922	156
Trauma	9	81303	298
Trauma	9	80026	441
Trauma	9	81249	81
Trauma	9	85222	278
Trauma	9	8784	141
Trauma	9	81333	1243
Trauma	9	82322	1174
Trauma	9	8500	1857
Trauma	9	82300	1605
Trauma	9	8442	428
Trauma	9	81323	936
Trauma	9	85181	303
Trauma	9	80131	196
Trauma	9	8208	407
Trauma	9	8786	196
Trauma	9	83401	29
Trauma	9	82111	232
Trauma	9	85241	489
Trauma	9	81242	1990

© 2021 Pelletier et al. *JAMA Network Open.*

Diagnosis	ICD Version	ICD Code	Number of Encounters
Trauma	9	81243	641
Trauma	9	81500	30
Trauma	9	85011	1418
Trauma	9	81601	79
Trauma	9	8261	300
Trauma	9	82320	760
Trauma	9	86399	44
Trauma	9	86329	274
Trauma	9	80101	1858
Trauma	9	8911	444
Trauma	9	8782	67
Trauma	9	8505	982
Trauma	9	81301	339
Trauma	9	920	904
Trauma	9	80326	75
Trauma	9	85246	75
Trauma	9	9213	230
Trauma	9	81305	122
Trauma	9	8832	164
Trauma	9	8249	314

© 2021 Pelletier et al. *JAMA Network Open.*

Diagnosis	ICD Version	ICD Code	Number of Encounters
Trauma	9	8708	79
Trauma	9	8024	451
Trauma	9	81502	17
Trauma	9	8242	170
Trauma	9	81354	756
Trauma	9	92400	91
Trauma	9	86501	153
Trauma	9	8240	394
Trauma	9	82001	452
Trauma	9	9222	259
Trauma	9	81344	818
Trauma	9	80322	65
Trauma	9	8910	428
Trauma	9	92810	38
Trauma	9	80120	356
Trauma	9	87349	74
Trauma	9	81611	242
Trauma	9	88102	31
Trauma	9	82022	763
Trauma	9	8363	100

© 2021 Pelletier et al. *JAMA Network Open.*

Diagnosis	ICD Version	ICD Code	Number of Encounters
Trauma	9	82101	8403
Trauma	9	80021	3219
Trauma	9	82332	489
Trauma	9	82382	249
Trauma	9	80220	88
Trauma	9	8244	589
Trauma	9	81200	169
Trauma	9	82122	376
Trauma	9	80102	429
Trauma	9	80001	2158
Trauma	9	81306	225
Trauma	9	82380	163
Trauma	9	937	63
Trauma	9	9352	755
Trauma	9	85301	317
Trauma	9	80112	99
Trauma	9	81612	191
Trauma	9	81321	122
Trauma	9	83501	90
Trauma	9	80122	607

© 2021 Pelletier et al. *JAMA Network Open.*

Diagnosis	ICD Version	ICD Code	Number of Encounters
Trauma	9	81201	84
Trauma	9	94423	85
Trauma	9	80224	84
Trauma	9	8470	254
Trauma	9	8448	41
Trauma	9	85306	129
Trauma	9	86500	412
Trauma	9	85201	655
Trauma	9	80106	329
Trauma	9	8054	688
Trauma	9	8052	616
Trauma	9	87352	90
Trauma	9	86602	544
Trauma	9	92720	59
Trauma	9	8730	388
Trauma	9	8220	255
Trauma	9	81002	277
Trauma	9	8082	522
Trauma	9	8080	243
Trauma	9	81209	176

© 2021 Pelletier et al. *JAMA Network Open.*

Diagnosis	ICD Version	ICD Code	Number of Encounters
Trauma	9	8860	286
Trauma	9	8702	221
Trauma	9	936	741
Trauma	9	8064	65
Trauma	9	8028	402
Trauma	9	81251	373
Trauma	9	80109	124
Trauma	9	82009	255
Trauma	9	9563	19
Trauma	9	80321	323
Trauma	9	80126	622
Trauma	9	87343	208
Trauma	9	81322	45
Trauma	9	81250	38
Trauma	9	81342	308
Trauma	9	9273	122
Trauma	9	80703	52
Trauma	9	82330	245
Trauma	9	82392	107
Trauma	9	80011	247

© 2021 Pelletier et al. *JAMA Network Open.*

Diagnosis	ICD Version	ICD Code	Number of Encounters
Trauma	9	87353	46
Trauma	9	87201	56
Trauma	9	85206	158
Trauma	9	9166	33
Trauma	9	80506	100
Trauma	9	86389	126
Trauma	9	80022	467
Trauma	9	87342	334
Trauma	9	81352	45
Trauma	9	82129	414
Trauma	9	80116	122
Trauma	9	82130	28
Trauma	9	87363	83
Trauma	9	85012	21
Trauma	9	80227	38
Trauma	9	88101	48
Trauma	9	9472	81
Trauma	9	83659	4
Trauma	9	8831	126
Trauma	9	8748	160

© 2021 Pelletier et al. *JAMA Network Open.*

Diagnosis	ICD Version	ICD Code	Number of Encounters
Trauma	9	8760	49
Trauma	9	81313	186
Trauma	9	80221	287
Trauma	9	81393	160
Trauma	9	85221	1381
Trauma	9	80502	203
Trauma	9	80501	160
Trauma	9	8509	398
Trauma	9	81221	420
Trauma	9	8438	44
Trauma	9	83901	120
Trauma	9	88100	117
Trauma	9	86503	941
Trauma	9	8796	180
Trauma	9	9348	206
Trauma	9	82120	786
Trauma	9	8246	276
Trauma	9	85106	15
Trauma	9	8744	99
Trauma	9	81252	26

© 2021 Pelletier et al. *JAMA Network Open.*

Diagnosis	ICD Version	ICD Code	Number of Encounters
Trauma	9	80019	13
Trauma	9	9116	12
Trauma	9	9341	866
Trauma	9	86405	520
Trauma	9	8912	212
Trauma	9	8770	56
Trauma	9	82123	251
Trauma	9	86121	501
Trauma	9	85220	661
Trauma	9	80141	128
Trauma	9	80071	130
Trauma	9	82121	197
Trauma	9	80228	142
Trauma	9	81332	73
Trauma	9	80841	240
Trauma	9	80051	200
Trauma	9	8026	616
Trauma	9	86403	417
Trauma	9	9514	15
Trauma	9	90003	12

© 2021 Pelletier et al. *JAMA Network Open.*

Diagnosis	ICD Version	ICD Code	Number of Encounters
Trauma	9	81353	33
Trauma	9	85226	315
Trauma	9	82521	97
Trauma	9	83500	289
Trauma	9	80081	21
Trauma	9	80225	251
Trauma	9	88003	53
Trauma	9	8056	127
Trauma	9	80507	92
Trauma	9	82100	644
Trauma	9	83809	6
Trauma	9	8921	323
Trauma	9	86803	66
Trauma	9	9195	8
Trauma	9	80229	128
Trauma	9	92619	30
Trauma	9	92820	61
Trauma	9	80072	37
Trauma	9	85141	60
Trauma	9	9349	287

© 2021 Pelletier et al. *JAMA Network Open.*

Diagnosis	ICD Version	ICD Code	Number of Encounters
Trauma	9	8600	524
Trauma	9	8029	39
Trauma	9	87341	242
Trauma	9	8701	95
Trauma	9	9583	265
Trauma	9	8260	52
Trauma	9	87365	153
Trauma	9	931	25
Trauma	9	938	213
Trauma	9	86345	89
Trauma	9	80238	49
Trauma	9	81240	167
Trauma	9	82525	160
Trauma	9	82301	25
Trauma	9	88111	43
Trauma	9	80012	68
Trauma	9	86402	198
Trauma	9	8749	68
Trauma	9	80000	373
Trauma	9	8711	357

© 2021 Pelletier et al. *JAMA Network Open.*

Diagnosis	ICD Version	ICD Code	Number of Encounters
Trauma	9	81311	36
Trauma	9	9160	72
Trauma	9	81304	24
Trauma	9	86413	54
Trauma	9	8250	73
Trauma	9	80222	162
Trauma	9	82302	171
Trauma	9	82021	282
Trauma	9	80236	86
Trauma	9	80226	251
Trauma	9	86509	350
Trauma	9	80040	12
Trauma	9	9331	1126
Trauma	9	82003	87
Trauma	9	81383	153
Trauma	9	9031	49
Trauma	9	80100	268
Trauma	9	80006	105
Trauma	9	8731	127
Trauma	9	86404	460

© 2021 Pelletier et al. *JAMA Network Open.*

Diagnosis	ICD Version	ICD Code	Number of Encounters
Trauma	9	86344	32
Trauma	9	85102	25
Trauma	9	8822	80
Trauma	9	80135	15
Trauma	9	9212	21
Trauma	9	9553	17
Trauma	9	80061	70
Trauma	9	9176	79
Trauma	9	9110	114
Trauma	9	80231	31
Trauma	9	86801	47
Trauma	9	932	65
Trauma	9	8449	45
Trauma	9	85101	42
Trauma	9	87375	14
Trauma	9	9100	266
Trauma	9	80032	39
Trauma	9	8830	49
Trauma	9	87369	17
Trauma	9	9330	173

© 2021 Pelletier et al. *JAMA Network Open.*

Diagnosis	ICD Version	ICD Code	Number of Encounters
Trauma	9	8020	279
Trauma	9	86352	23
Trauma	9	80059	5
Trauma	9	81231	129
Trauma	9	80139	11
Trauma	9	86504	244
Trauma	9	82340	16
Trauma	9	9106	15
Trauma	9	87374	22
Trauma	9	81308	113
Trauma	9	80002	188
Trauma	9	80020	555
Trauma	9	87344	61
Trauma	9	92821	7
Trauma	9	82535	153
Trauma	9	86400	59
Trauma	9	8750	78
Trauma	9	82381	17
Trauma	9	81417	2
Trauma	9	9470	63

© 2021 Pelletier et al. *JAMA Network Open.*

Diagnosis	ICD Version	ICD Code	Number of Encounters
Trauma	9	85223	13
Trauma	9	80016	62
Trauma	9	9340	214
Trauma	9	85146	24
Trauma	9	82002	78
Trauma	9	80129	96
Trauma	9	81244	262
Trauma	9	8620	21
Trauma	9	8027	13
Trauma	9	87279	4
Trauma	9	86321	139
Trauma	9	85225	127
Trauma	9	80235	51
Trauma	9	8370	13
Trauma	9	85209	27
Trauma	9	9350	26
Trauma	9	8920	122
Trauma	9	81504	17
Trauma	9	80009	64
Trauma	9	9283	24

© 2021 Pelletier et al. *JAMA Network Open.*

Diagnosis	ICD Version	ICD Code	Number of Encounters
Trauma	9	92411	30
Trauma	9	9565	6
Trauma	9	92800	11
Trauma	9	80076	67
Trauma	9	80029	55
Trauma	9	9136	8
Trauma	9	81001	62
Trauma	9	80130	37
Trauma	9	80503	31
Trauma	9	85200	173
Trauma	9	85302	104
Trauma	9	80124	34
Trauma	9	85204	6
Trauma	9	83200	15
Trauma	9	86229	38
Trauma	9	87351	168
Trauma	9	92232	28
Trauma	9	86611	6
Trauma	9	86510	55
Trauma	9	8792	112

© 2021 Pelletier et al. *JAMA Network Open.*

Diagnosis	ICD Version	ICD Code	Number of Encounters
Trauma	9	86330	68
Trauma	9	85186	152
Trauma	9	9560	6
Trauma	9	8700	97
Trauma	9	80600	49
Trauma	9	80702	60
Trauma	9	81382	7
Trauma	9	8821	228
Trauma	9	8932	17
Trauma	9	87362	17
Trauma	9	80014	10
Trauma	9	80039	7
Trauma	9	9156	2
Trauma	9	9178	9
Trauma	9	80025	70
Trauma	9	80171	36
Trauma	9	88112	16
Trauma	9	94424	48
Trauma	9	85402	148
Trauma	9	92310	15

© 2021 Pelletier et al. *JAMA Network Open.*

Diagnosis	ICD Version	ICD Code	Number of Encounters
Trauma	9	81000	199
Trauma	9	94202	15
Trauma	9	86600	144
Trauma	9	8850	82
Trauma	9	80070	48
Trauma	9	86331	17
Trauma	9	9582	33
Trauma	9	87269	10
Trauma	9	87364	97
Trauma	9	8670	95
Trauma	9	80010	58
Trauma	9	80031	323
Trauma	9	90453	7
Trauma	9	8713	6
Trauma	9	87211	24
Trauma	9	80132	73
Trauma	9	9224	83
Trauma	9	8072	37
Trauma	9	81109	17
Trauma	9	87200	29

© 2021 Pelletier et al. *JAMA Network Open.*

Diagnosis	ICD Version	ICD Code	Number of Encounters
Trauma	9	86804	43
Trauma	9	81602	26
Trauma	9	87361	27
Trauma	9	81513	23
Trauma	9	9473	7
Trauma	9	88122	75
Trauma	9	9587	139
Trauma	9	81345	21
Trauma	9	87371	9
Trauma	9	80036	49
Trauma	9	9529	82
Trauma	9	9586	25
Trauma	9	9251	13
Trauma	9	9244	9
Trauma	9	85309	19
Trauma	9	95204	20
Trauma	9	85180	89
Trauma	9	8788	16
Trauma	9	8900	212
Trauma	9	80091	10

© 2021 Pelletier et al. *JAMA Network Open.*

Diagnosis	ICD Version	ICD Code	Number of Encounters
Trauma	9	82390	39
Trauma	9	84509	24
Trauma	9	85240	87
Trauma	9	8221	69
Trauma	9	81501	14
Trauma	9	8931	38
Trauma	9	80849	27
Trauma	9	81003	36
Trauma	9	80111	187
Trauma	9	8794	72
Trauma	9	85205	60
Trauma	9	86502	210
Trauma	9	8488	34
Trauma	9	85242	78
Trauma	9	9522	20
Trauma	9	8021	45
Trauma	9	87354	19
Trauma	9	80146	49
Trauma	9	81220	73
Trauma	9	92410	48

© 2021 Pelletier et al. *JAMA Network Open.*

Diagnosis	ICD Version	ICD Code	Number of Encounters
Trauma	9	80301	101
Trauma	9	80024	21
Trauma	9	8025	52
Trauma	9	82020	68
Trauma	9	8717	9
Trauma	9	80504	67
Trauma	9	95892	83
Trauma	9	94224	340
Trauma	9	82321	41
Trauma	9	83104	19
Trauma	9	94320	32
Trauma	9	87350	28
Trauma	9	80844	45
Trauma	9	92300	6
Trauma	9	87412	3
Trauma	9	80136	72
Trauma	9	85182	141
Trauma	9	90253	6
Trauma	9	8840	22
Trauma	9	88002	17

© 2021 Pelletier et al. *JAMA Network Open.*

Diagnosis	ICD Version	ICD Code	Number of Encounters
Trauma	9	80705	26
Trauma	9	9050	21
Trauma	9	94229	114
Trauma	9	8842	2
Trauma	9	83920	54
Trauma	9	8704	45
Trauma	9	83502	2
Trauma	9	8761	30
Trauma	9	83651	2
Trauma	9	8502	24
Trauma	9	9120	16
Trauma	9	94427	48
Trauma	9	83412	16
Trauma	9	80015	16
Trauma	9	94321	281
Trauma	9	82341	4
Trauma	9	9146	6
Trauma	9	87360	22
Trauma	9	9177	83
Trauma	9	80302	17

© 2021 Pelletier et al. *JAMA Network Open.*

Diagnosis	ICD Version	ICD Code	Number of Encounters
Trauma	9	80151	66
Trauma	9	94434	30
Trauma	9	88120	78
Trauma	9	8360	36
Trauma	9	8820	96
Trauma	9	80149	14
Trauma	9	80065	12
Trauma	9	81613	19
Trauma	9	8361	56
Trauma	9	94431	15
Trauma	9	8300	8
Trauma	9	80609	29
Trauma	9	85202	187
Trauma	9	9194	18
Trauma	9	8751	38
Trauma	9	86355	42
Trauma	9	80380	1
Trauma	9	80237	10
Trauma	9	9042	8
Trauma	9	80191	6

© 2021 Pelletier et al. *JAMA Network Open.*

Diagnosis	ICD Version	ICD Code	Number of Encounters
Trauma	9	9221	66
Trauma	9	82310	66
Trauma	9	80842	34
Trauma	9	83652	6
Trauma	9	82522	13
Trauma	9	81401	20
Trauma	9	92231	60
Trauma	9	88110	101
Trauma	9	81100	12
Trauma	9	83101	7
Trauma	9	8472	24
Trauma	9	8408	7
Trauma	9	90225	3
Trauma	9	81203	13
Trauma	9	80604	21
Trauma	9	8439	29
Trauma	9	8440	7
Trauma	9	8471	8
Trauma	9	80603	5
Trauma	9	85191	18

© 2021 Pelletier et al. *JAMA Network Open.*

Diagnosis	ICD Version	ICD Code	Number of Encounters
Trauma	9	95200	80
Trauma	9	80052	17
Trauma	9	84500	41
Trauma	9	8485	2
Trauma	9	8479	19
Trauma	9	8441	14
Trauma	9	81610	15
Trauma	9	86603	111
Trauma	9	9167	11
Trauma	9	85405	45
Trauma	9	8400	5
Trauma	9	9570	13
Trauma	9	9552	27
Trauma	9	86401	92
Trauma	9	8247	7
Trauma	9	83219	1
Trauma	9	9147	14
Trauma	9	81259	6
Trauma	9	80142	63
Trauma	9	81331	37

© 2021 Pelletier et al. *JAMA Network Open.*

Diagnosis	ICD Version	ICD Code	Number of Encounters
Trauma	9	83961	37
Trauma	9	81318	49
Trauma	9	83202	9
Trauma	9	82531	25
Trauma	9	82131	33
Trauma	9	9390	19
Trauma	9	8787	14
Trauma	9	8902	6
Trauma	9	8716	74
Trauma	9	8483	1
Trauma	9	86339	110
Trauma	9	9032	27
Trauma	9	80110	58
Trauma	9	80075	30
Trauma	9	85229	52
Trauma	9	80170	20
Trauma	9	9134	6
Trauma	9	8604	102
Trauma	9	83650	20
Trauma	9	92710	16

© 2021 Pelletier et al. *JAMA Network Open.*

Diagnosis	ICD Version	ICD Code	Number of Encounters
Trauma	9	82132	19
Trauma	9	9165	42
Trauma	9	86409	34
Trauma	9	86131	32
Trauma	9	84510	2
Trauma	9	8245	36
Trauma	9	8960	46
Trauma	9	86384	125
Trauma	9	87330	11
Trauma	9	94520	29
Trauma	9	94526	369
Trauma	9	94223	289
Trauma	9	94536	157
Trauma	9	94534	284
Trauma	9	94134	8
Trauma	9	94525	30
Trauma	9	94127	335
Trauma	9	94225	109
Trauma	9	94234	179
Trauma	9	8088	35

© 2021 Pelletier et al. *JAMA Network Open.*

Diagnosis	ICD Version	ICD Code	Number of Encounters
Trauma	9	94232	175
Trauma	9	81254	42
Trauma	9	80060	27
Trauma	9	80161	24
Trauma	9	94222	673
Trauma	9	9181	56
Trauma	9	94406	5
Trauma	9	80176	32
Trauma	9	80300	49
Trauma	9	86415	57
Trauma	9	8710	189
Trauma	9	94322	17
Trauma	9	8951	37
Trauma	9	86512	16
Trauma	9	94436	65
Trauma	9	9581	10
Trauma	9	80239	37
Trauma	9	94420	179
Trauma	9	9012	1
Trauma	9	8241	53

© 2021 Pelletier et al. *JAMA Network Open.*

Diagnosis	ICD Version	ICD Code	Number of Encounters
Trauma	9	94335	53
Trauma	9	80023	20
Trauma	9	9144	9
Trauma	9	9061	10
Trauma	9	94522	357
Trauma	9	9115	17
Trauma	9	85401	256
Trauma	9	87332	3
Trauma	9	94314	1
Trauma	9	86222	35
Trauma	9	94203	10
Trauma	9	80320	107
Trauma	9	95216	4
Trauma	9	85400	134
Trauma	9	86601	81
Trauma	9	94120	324
Trauma	9	94110	14
Trauma	9	80050	24
Trauma	9	80115	12
Trauma	9	94425	306

© 2021 Pelletier et al. *JAMA Network Open.*

Diagnosis	ICD Version	ICD Code	Number of Encounters
Trauma	9	94139	53
Trauma	9	80505	79
Trauma	9	80062	20
Trauma	9	94422	13
Trauma	9	80704	36
Trauma	9	94426	101
Trauma	9	80125	108
Trauma	9	80082	8
Trauma	9	9081	2
Trauma	9	80421	36
Trauma	9	94532	229
Trauma	9	94443	5
Trauma	9	9066	3
Trauma	9	87339	6
Trauma	9	86612	36
Trauma	9	94123	26
Trauma	9	94129	305
Trauma	9	86381	52
Trauma	9	88013	28
Trauma	9	95210	8

© 2021 Pelletier et al. *JAMA Network Open.*

Diagnosis	ICD Version	ICD Code	Number of Encounters
Trauma	9	86414	63
Trauma	9	94124	92
Trauma	9	80312	7
Trauma	9	85140	10
Trauma	9	94239	78
Trauma	9	80620	22
Trauma	9	80085	5
Trauma	9	80035	14
Trauma	9	9535	1
Trauma	9	8715	27
Trauma	9	82019	5
Trauma	9	94128	145
Trauma	9	80080	10
Trauma	9	81317	2
Trauma	9	9463	8
Trauma	9	80331	46
Trauma	9	80123	15
Trauma	9	94233	123
Trauma	9	8605	57
Trauma	9	9584	19

© 2021 Pelletier et al. *JAMA Network Open.*

Diagnosis	ICD Version	ICD Code	Number of Encounters
Trauma	9	94539	88
Trauma	9	94435	61
Trauma	9	80030	58
Trauma	9	9471	22
Trauma	9	9402	12
Trauma	9	86341	19
Trauma	9	8631	42
Trauma	9	94542	15
Trauma	9	86383	45
Trauma	9	94103	6
Trauma	9	94524	249
Trauma	9	80140	25
Trauma	9	87402	15
Trauma	9	87340	71
Trauma	9	94126	49
Trauma	9	90089	9
Trauma	9	9401	29
Trauma	9	88019	11
Trauma	9	95205	31
Trauma	9	94529	84

© 2021 Pelletier et al. *JAMA Network Open.*

Diagnosis	ICD Version	ICD Code	Number of Encounters
Trauma	9	90442	2
Trauma	9	8603	20
Trauma	9	92703	6
Trauma	9	83942	7
Trauma	9	82133	17
Trauma	9	85406	137
Trauma	9	94137	32
Trauma	9	8057	4
Trauma	9	94121	58
Trauma	9	81253	29
Trauma	9	80309	9
Trauma	9	9509	2
Trauma	9	9180	16
Trauma	9	9248	24
Trauma	9	80346	6
Trauma	9	9060	8
Trauma	9	95206	5
Trauma	9	82139	30
Trauma	9	86613	9
Trauma	9	80430	1

© 2021 Pelletier et al. *JAMA Network Open.*

Diagnosis	ICD Version	ICD Code	Number of Encounters
Trauma	9	83908	15
Trauma	9	95891	55
Trauma	9	8251	53
Trauma	9	86320	61
Trauma	9	80701	72
Trauma	9	94428	115
Trauma	9	87410	3
Trauma	9	86342	14
Trauma	9	8714	27
Trauma	9	82312	44
Trauma	9	86814	10
Trauma	9	94133	9
Trauma	9	8798	7
Trauma	9	81351	12
Trauma	9	87359	41
Trauma	9	80119	9
Trauma	9	85212	1
Trauma	9	9588	37
Trauma	9	82532	4
Trauma	9	9114	5

© 2021 Pelletier et al. *JAMA Network Open.*

**Diagnosis**	**ICD Version**	**ICD Code**	**Number of Encounters**
Trauma	9	94102	1
Trauma	9	85203	4
Trauma	9	80325	34
Trauma	9	80376	13
Trauma	9	94401	4
Trauma	9	90254	7
Trauma	9	80420	11
Trauma	9	86354	14
Trauma	9	85145	5
Trauma	9	8678	3
Trauma	9	80113	4
Trauma	9	9040	14
Trauma	9	9124	2
Trauma	9	8781	12
Trauma	9	85300	95
Trauma	9	80041	57
Trauma	9	90081	2
Trauma	9	80843	30
Trauma	9	85304	5
Trauma	9	81211	7

© 2021 Pelletier et al. *JAMA Network Open.*

Diagnosis	ICD Version	ICD Code	Number of Encounters
Trauma	9	9074	3
Trauma	9	94800	663
Trauma	9	83816	2
Trauma	9	94300	7
Trauma	9	80045	4
Trauma	9	80172	13
Trauma	9	87401	8
Trauma	9	94323	160
Trauma	9	85142	25
Trauma	9	86103	3
Trauma	9	94438	62
Trauma	9	94319	1
Trauma	9	8074	9
Trauma	9	94131	10
Trauma	9	94136	29
Trauma	9	8970	18
Trauma	9	80636	2
Trauma	9	94325	87
Trauma	9	82520	7
Trauma	9	94214	10

© 2021 Pelletier et al. *JAMA Network Open.*

Diagnosis	ICD Version	ICD Code	Number of Encounters
Trauma	9	94219	3
Trauma	9	87320	44
Trauma	9	80625	31
Trauma	9	9010	12
Trauma	9	94411	4
Trauma	9	80042	13
Trauma	9	85305	24
Trauma	9	9288	6
Trauma	9	9219	10
Trauma	9	86382	63
Trauma	9	9308	3
Trauma	9	9145	12
Trauma	9	94433	43
Trauma	9	92811	3
Trauma	9	94100	13
Trauma	9	81503	16
Trauma	9	80605	37
Trauma	9	94333	129
Trauma	9	94305	2
Trauma	9	94503	3

© 2021 Pelletier et al. *JAMA Network Open.*

Diagnosis	ICD Version	ICD Code	Number of Encounters
Trauma	9	94530	25
Trauma	9	86100	1
Trauma	9	94445	6
Trauma	9	80094	2
Trauma	9	94405	8
Trauma	9	94331	143
Trauma	9	85105	3
Trauma	9	9107	5
Trauma	9	94122	30
Trauma	9	94430	81
Trauma	9	85409	43
Trauma	9	94109	4
Trauma	9	94101	2
Trauma	9	94230	13
Trauma	9	94339	69
Trauma	9	94446	3
Trauma	9	8602	21
Trauma	9	80706	15
Trauma	9	86419	7
Trauma	9	86813	56

© 2021 Pelletier et al. *JAMA Network Open.*

Diagnosis	ICD Version	ICD Code	Number of Encounters
Trauma	9	81341	62
Trauma	9	9070	26
Trauma	9	90001	8
Trauma	9	8601	47
Trauma	9	9534	55
Trauma	9	86513	56
Trauma	9	80629	15
Trauma	9	9041	18
Trauma	9	8941	5
Trauma	9	86514	19
Trauma	9	83905	11
Trauma	9	81102	3
Trauma	9	83921	6
Trauma	9	8371	10
Trauma	9	8630	29
Trauma	9	92801	7
Trauma	9	86511	5
Trauma	9	85403	7
Trauma	9	9562	4
Trauma	9	80181	6

© 2021 Pelletier et al. *JAMA Network Open.*

Diagnosis	ICD Version	ICD Code	Number of Encounters
Trauma	9	82533	5
Trauma	9	9164	19
Trauma	9	94410	3
Trauma	9	88012	7
Trauma	9	9001	5
Trauma	9	86800	28
Trauma	9	8871	12
Trauma	9	94117	4
Trauma	9	9150	11
Trauma	9	94519	2
Trauma	9	81514	15
Trauma	9	9140	15
Trauma	9	94212	15
Trauma	9	8672	7
Trauma	9	9170	12
Trauma	9	9065	3
Trauma	9	94311	8
Trauma	9	81230	10
Trauma	9	9163	3
Trauma	9	86132	28

© 2021 Pelletier et al. *JAMA Network Open.*

Diagnosis	ICD Version	ICD Code	Number of Encounters
Trauma	9	8851	7
Trauma	9	9130	29
Trauma	9	86101	26
Trauma	9	94329	79
Trauma	9	92401	43
Trauma	9	85185	25
Trauma	9	94235	6
Trauma	9	84200	3
Trauma	9	9579	2
Trauma	9	80003	1
Trauma	9	85189	18
Trauma	9	94130	41
Trauma	9	85235	4
Trauma	9	86122	17
Trauma	9	87373	9
Trauma	9	9109	2
Trauma	9	86351	6
Trauma	9	94249	5
Trauma	9	9068	5
Trauma	9	80707	7

© 2021 Pelletier et al. *JAMA Network Open.*

Diagnosis	ICD Version	ICD Code	Number of Encounters
Trauma	9	80180	5
Trauma	9	81307	14
Trauma	9	94404	3
Trauma	9	8973	4
Trauma	9	85252	3
Trauma	9	94502	17
Trauma	9	80063	1
Trauma	9	82110	19
Trauma	9	80601	18
Trauma	9	9172	8
Trauma	9	94324	13
Trauma	9	80621	16
Trauma	9	8961	31
Trauma	9	88010	12
Trauma	9	94118	2
Trauma	9	94810	18
Trauma	9	80470	3
Trauma	9	8861	33
Trauma	9	94400	11
Trauma	9	94220	9

© 2021 Pelletier et al. *JAMA Network Open.*

Diagnosis	ICD Version	ICD Code	Number of Encounters
Trauma	9	94138	39
Trauma	9	95203	15
Trauma	9	85183	6
Trauma	9	8083	16
Trauma	9	86809	28
Trauma	9	81510	18
Trauma	9	94204	11
Trauma	9	80708	22
Trauma	9	80066	25
Trauma	9	81210	10
Trauma	9	88011	6
Trauma	9	85404	6
Trauma	9	94116	3
Trauma	9	83804	3
Trauma	9	8170	11
Trauma	9	94213	4
Trauma	9	80234	16
Trauma	9	80626	11
Trauma	9	9493	1
Trauma	9	80152	12

© 2021 Pelletier et al. *JAMA Network Open.*

Diagnosis	ICD Version	ICD Code	Number of Encounters
Trauma	9	80004	1
Trauma	9	8065	9
Trauma	9	86394	9
Trauma	9	8068	1
Trauma	9	94421	23
Trauma	9	8793	26
Trauma	9	94415	7
Trauma	9	9211	27
Trauma	9	80034	2
Trauma	9	86392	7
Trauma	9	80056	14
Trauma	9	8795	32
Trauma	9	9229	3
Trauma	9	85195	26
Trauma	9	80366	1
Trauma	9	92303	7
Trauma	9	85224	11
Trauma	9	80360	2
Trauma	9	81512	12
Trauma	9	94125	21

© 2021 Pelletier et al. *JAMA Network Open.*

Diagnosis	ICD Version	ICD Code	Number of Encounters
Trauma	9	81101	5
Trauma	9	9102	5
Trauma	9	94418	3
Trauma	9	94416	1
Trauma	9	8739	6
Trauma	9	80709	43
Trauma	9	81343	23
Trauma	9	8799	6
Trauma	9	80392	1
Trauma	9	8950	135
Trauma	9	8771	34
Trauma	9	9260	7
Trauma	9	94546	6
Trauma	9	80606	17
Trauma	9	94820	4
Trauma	9	86819	11
Trauma	9	8671	23
Trauma	9	8712	37
Trauma	9	80074	7
Trauma	9	86239	16

© 2021 Pelletier et al. *JAMA Network Open.*

Diagnosis	ICD Version	ICD Code	Number of Encounters
Trauma	9	9162	3
Trauma	9	95219	3
Trauma	9	8728	9
Trauma	9	94437	16
Trauma	9	85190	8
Trauma	9	82032	14
Trauma	9	9054	4
Trauma	9	81010	6
Trauma	9	80163	1
Trauma	9	81381	16
Trauma	9	9108	16
Trauma	9	94523	27
Trauma	9	92721	1
Trauma	9	83503	11
Trauma	9	85149	3
Trauma	9	85151	1
Trauma	9	85415	4
Trauma	9	8780	49
Trauma	9	81511	14
Trauma	9	8629	6

© 2021 Pelletier et al. *JAMA Network Open.*

Diagnosis	ICD Version	ICD Code	Number of Encounters
Trauma	9	9135	14
Trauma	9	85315	1
Trauma	9	86110	4
Trauma	9	80511	1
Trauma	9	81391	5
Trauma	9	80611	1
Trauma	9	9131	3
Trauma	9	80230	17
Trauma	9	9492	2
Trauma	9	81392	12
Trauma	9	94513	2
Trauma	9	80508	32
Trauma	9	9083	5
Trauma	9	9137	5
Trauma	9	88001	8
Trauma	9	8676	5
Trauma	9	94113	5
Trauma	9	9141	3
Trauma	9	80329	4
Trauma	9	81600	7

© 2021 Pelletier et al. *JAMA Network Open.*

Diagnosis	ICD Version	ICD Code	Number of Encounters
Trauma	9	83906	9
Trauma	9	9-E9330	1
Trauma	9	8621	30
Trauma	9	83904	6
Trauma	9	94533	43
Trauma	9	8418	5
Trauma	9	9033	14
Trauma	9	8399	1
Trauma	9	88000	28
Trauma	9	9309	3
Trauma	9	80500	13
Trauma	9	8719	29
Trauma	9	9067	5
Trauma	9	80332	7
Trauma	9	8745	21
Trauma	9	83410	1
Trauma	9	87271	2
Trauma	9	82031	2
Trauma	9	80223	2
Trauma	9	88009	11

© 2021 Pelletier et al. *JAMA Network Open.*

Diagnosis	ICD Version	ICD Code	Number of Encounters
Trauma	9	9132	2
Trauma	9	82534	3
Trauma	9	85311	2
Trauma	9	94301	3
Trauma	9	95208	10
Trauma	9	9404	17
Trauma	9	80046	19
Trauma	9	80385	2
Trauma	9	94549	3
Trauma	9	80425	5
Trauma	9	81111	3
Trauma	9	80175	17
Trauma	9	9500	9
Trauma	9	80853	7
Trauma	9	94349	3
Trauma	9	9039	3
Trauma	9	80624	4
Trauma	9	85215	4
Trauma	9	9249	6
Trauma	9	94105	1

© 2021 Pelletier et al. *JAMA Network Open.*

Diagnosis	ICD Version	ICD Code	Number of Encounters
Trauma	9	9105	14
Trauma	9	94303	3
Trauma	9	81314	7
Trauma	9	80095	3
Trauma	9	82311	7
Trauma	9	94244	7
Trauma	9	94535	22
Trauma	9	86410	4
Trauma	9	80335	8
Trauma	9	80336	5
Trauma	9	83969	8
Trauma	9	86340	5
Trauma	9	85170	2
Trauma	9	85175	3
Trauma	9	80090	5
Trauma	9	80365	2
Trauma	9	94504	7
Trauma	9	8171	15
Trauma	9	9095	1
Trauma	9	94221	5

© 2021 Pelletier et al. *JAMA Network Open.*

Diagnosis	ICD Version	ICD Code	Number of Encounters
Trauma	9	95201	2
Trauma	9	85135	3
Trauma	9	85184	10
Trauma	9	85171	4
Trauma	9	81212	1
Trauma	9	80700	9
Trauma	9	8243	19
Trauma	9	90002	1
Trauma	9	81312	1
Trauma	9	9190	34
Trauma	9	92420	12
Trauma	9	95211	6
Trauma	9	86350	8
Trauma	9	80166	9
Trauma	9	80426	12
Trauma	9	90222	3
Trauma	9	94330	26
Trauma	9	94340	1
Trauma	9	85130	4
Trauma	9	85210	3

© 2021 Pelletier et al. *JAMA Network Open.*

Diagnosis	ICD Version	ICD Code	Number of Encounters
Trauma	9	90223	3
Trauma	9	82000	13
Trauma	9	80330	7
Trauma	9	80086	8
Trauma	9	80435	2
Trauma	9	94112	3
Trauma	9	85196	7
Trauma	9	90301	5
Trauma	9	9019	1
Trauma	9	9490	5
Trauma	9	80150	10
Trauma	9	84503	9
Trauma	9	9161	20
Trauma	9	87329	13
Trauma	9	9300	12
Trauma	9	83209	7
Trauma	9	81390	4
Trauma	9	80103	3
Trauma	9	86412	22
Trauma	9	86390	2

© 2021 Pelletier et al. *JAMA Network Open.*

Diagnosis	ICD Version	ICD Code	Number of Encounters
Trauma	9	94531	13
Trauma	9	8940	15
Trauma	9	80371	18
Trauma	9	80079	7
Trauma	9	94343	1
Trauma	9	81340	2
Trauma	9	94215	5
Trauma	9	8930	14
Trauma	9	94412	2
Trauma	9	9400	5
Trauma	9	82523	14
Trauma	9	86810	9
Trauma	9	86353	6
Trauma	9	92421	2
Trauma	9	94334	8
Trauma	9	94511	1
Trauma	9	94341	3
Trauma	9	9157	7
Trauma	9	94242	5
Trauma	9	86349	4

© 2021 Pelletier et al. *JAMA Network Open.*

Diagnosis	ICD Version	ICD Code	Number of Encounters
Trauma	9	94344	3
Trauma	9	94243	5
Trauma	9	80055	2
Trauma	9	94115	2
Trauma	9	9118	14
Trauma	9	82331	8
Trauma	9	81315	9
Trauma	9	9138	5
Trauma	9	9046	1
Trauma	9	85236	3
Trauma	9	9530	5
Trauma	9	83902	27
Trauma	9	9104	12
Trauma	9	80306	7
Trauma	9	80159	4
Trauma	9	87202	4
Trauma	9	80476	2
Trauma	9	81202	5
Trauma	9	86802	10
Trauma	9	80406	2

© 2021 Pelletier et al. *JAMA Network Open.*

Diagnosis	ICD Version	ICD Code	Number of Encounters
Trauma	9	86113	12
Trauma	9	9111	3
Trauma	9	9556	15
Trauma	9	90226	1
Trauma	9	87210	9
Trauma	9	9551	15
Trauma	9	9239	4
Trauma	9	80145	12
Trauma	9	8460	6
Trauma	9	81347	7
Trauma	9	9392	26
Trauma	9	80185	2
Trauma	9	81012	10
Trauma	9	8872	7
Trauma	9	8504	4
Trauma	9	81103	5
Trauma	9	9043	1
Trauma	9	9571	19
Trauma	9	80602	1
Trauma	9	9510	1

© 2021 Pelletier et al. *JAMA Network Open.*

Diagnosis	ICD Version	ICD Code	Number of Encounters
Trauma	9	80064	5
Trauma	9	81403	1
Trauma	9	90441	15
Trauma	9	86359	2
Trauma	9	8972	3
Trauma	9	80096	3
Trauma	9	83114	1
Trauma	9	8280	1
Trauma	9	80156	11
Trauma	9	81219	6
Trauma	9	85249	8
Trauma	9	92321	1
Trauma	9	9559	5
Trauma	9	87321	7
Trauma	9	8797	17
Trauma	9	88023	12
Trauma	9	80134	6
Trauma	9	80851	28
Trauma	9	81519	6
Trauma	9	80412	1

© 2021 Pelletier et al. *JAMA Network Open.*

Diagnosis	ICD Version	ICD Code	Number of Encounters
Trauma	9	87379	1
Trauma	9	83210	5
Trauma	9	9047	3
Trauma	9	9174	6
Trauma	9	81418	1
Trauma	9	92311	12
Trauma	9	83819	2
Trauma	9	85144	2
Trauma	9	94315	1
Trauma	9	9462	9
Trauma	9	94135	5
Trauma	9	9179	5
Trauma	9	8690	10
Trauma	9	9075	1
Trauma	9	80370	6
Trauma	9	86120	2
Trauma	9	8058	6
Trauma	9	86610	5
Trauma	9	9072	4
Trauma	9	9086	5

© 2021 Pelletier et al. *JAMA Network Open.*

Diagnosis	ICD Version	ICD Code	Number of Encounters
Trauma	9	8790	3
Trauma	9	87372	3
Trauma	9	9290	7
Trauma	9	94231	9
Trauma	9	94209	8
Trauma	9	8783	8
Trauma	9	80386	3
Trauma	9	80616	4
Trauma	9	8971	8
Trauma	9	94146	2
Trauma	9	80199	1
Trauma	9	92611	1
Trauma	9	90451	5
Trauma	9	9399	1
Trauma	9	80607	3
Trauma	9	83102	5
Trauma	9	94332	13
Trauma	9	94545	2
Trauma	9	94544	19
Trauma	9	94552	1

© 2021 Pelletier et al. *JAMA Network Open.*

Diagnosis	ICD Version	ICD Code	Number of Encounters
Trauma	9	80316	7
Trauma	9	80614	2
Trauma	9	86391	3
Trauma	9	94516	9
Trauma	9	80311	6
Trauma	9	94156	1
Trauma	9	94342	2
Trauma	9	94450	1
Trauma	9	80162	5
Trauma	9	94432	6
Trauma	9	94541	2
Trauma	9	86343	10
Trauma	9	94453	3
Trauma	9	94521	10
Trauma	9	9112	2
Trauma	9	94140	1
Trauma	9	8870	14
Trauma	9	80615	2
Trauma	9	80608	2
Trauma	9	94444	3

© 2021 Pelletier et al. *JAMA Network Open.*

Diagnosis	ICD Version	ICD Code	Number of Encounters
Trauma	9	8873	5
Trauma	9	86811	1
Trauma	9	8362	14
Trauma	9	80310	5
Trauma	9	9528	8
Trauma	9	82524	8
Trauma	9	83203	2
Trauma	9	80315	2
Trauma	9	82342	16
Trauma	9	86393	7
Trauma	9	95215	10
Trauma	9	87212	1
Trauma	9	80174	3
Trauma	9	82529	7
Trauma	9	8075	19
Trauma	9	8407	9
Trauma	9	9210	6
Trauma	9	83100	7
Trauma	9	87322	3
Trauma	9	9173	14

© 2021 Pelletier et al. *JAMA Network Open.*

Diagnosis	ICD Version	ICD Code	Number of Encounters
Trauma	9	90241	6
Trauma	9	84211	1
Trauma	9	80186	3
Trauma	9	9121	2
Trauma	9	9059	3
Trauma	9	8290	10
Trauma	9	8628	5
Trauma	9	9393	2
Trauma	9	86519	4
Trauma	9	80375	11
Trauma	9	9051	1
Trauma	9	81119	3
Trauma	9	9245	7
Trauma	9	83903	7
Trauma	9	88121	8
Trauma	9	80165	7
Trauma	9	86111	2
Trauma	9	80485	1
Trauma	9	80422	8
Trauma	9	85164	2

© 2021 Pelletier et al. *JAMA Network Open.*

Diagnosis	ICD Version	ICD Code	Number of Encounters
Trauma	9	82539	4
Trauma	9	94506	9
Trauma	9	94458	1
Trauma	9	80364	1
Trauma	9	8738	9
Trauma	9	83411	2
Trauma	9	87370	2
Trauma	9	83907	2
Trauma	9	80410	1
Trauma	9	94501	1
Trauma	9	9123	1
Trauma	9	94407	1
Trauma	9	80662	3
Trauma	9	94452	1
Trauma	9	94822	2
Trauma	9	94866	3
Trauma	9	94821	1
Trauma	9	90221	2
Trauma	9	8076	4
Trauma	9	9569	2

© 2021 Pelletier et al. *JAMA Network Open.*

Diagnosis	ICD Version	ICD Code	Number of Encounters
Trauma	9	80610	3
Trauma	9	8841	4
Trauma	9	83212	2
Trauma	9	94351	2
Trauma	9	94119	10
Trauma	9	8322	7
Trauma	9	86231	1
Trauma	9	94440	2
Trauma	9	94447	1
Trauma	9	95893	1
Trauma	9	9555	2
Trauma	9	86221	4
Trauma	9	90229	1
Trauma	9	95209	10
Trauma	9	9228	4
Trauma	9	9524	1
Trauma	9	9-E8498	1
Trauma	9	90082	2
Trauma	9	9539	2
Trauma	9	92320	15

© 2021 Pelletier et al. *JAMA Network Open.*

Diagnosis	ICD Version	ICD Code	Number of Encounters
Trauma	9	80859	1
Trauma	9	81509	4
Trauma	9	94107	6
Trauma	9	81110	4
Trauma	9	80341	2
Trauma	9	9117	2
Trauma	9	80441	1
Trauma	9	9550	1
Trauma	9	8709	1
Trauma	9	8059	1
Trauma	9	83900	2
Trauma	9	87261	3
Trauma	9	9171	9
Trauma	9	83803	4
Trauma	9	85416	2
Trauma	9	81415	1
Trauma	9	9048	3
Trauma	9	80513	2
Trauma	9	8791	4
Trauma	9	9198	2

© 2021 Pelletier et al. *JAMA Network Open.*

Diagnosis	ICD Version	ICD Code	Number of Encounters
Trauma	9	80143	2
Trauma	9	95899	1
Trauma	9	9009	1
Trauma	9	94403	2
Trauma	9	8419	5
Trauma	9	80372	1
Trauma	9	81316	3
Trauma	9	85104	2
Trauma	9	9159	2
Trauma	9	84210	2
Trauma	9	9020	12
Trauma	9	9168	6
Trauma	9	9460	2
Trauma	9	94205	8
Trauma	9	80189	1
Trauma	9	94114	2
Trauma	9	9149	2
Trauma	9	94417	1
Trauma	9	9409	2
Trauma	9	9169	5

© 2021 Pelletier et al. *JAMA Network Open.*

Diagnosis	ICD Version	ICD Code	Number of Encounters
Trauma	9	88022	3
Trauma	9	8489	1
Trauma	9	85411	4
Trauma	9	80160	10
Trauma	9	9557	7
Trauma	9	8691	3
Trauma	9	8679	3
Trauma	9	9035	2
Trauma	9	9233	3
Trauma	9	80232	8
Trauma	9	9268	7
Trauma	9	82011	2
Trauma	9	85100	8
Trauma	9	9155	5
Trauma	9	80351	6
Trauma	9	81302	10
Trauma	9	9175	3
Trauma	9	88020	1
Trauma	9	86112	6
Trauma	9	9152	4

© 2021 Pelletier et al. *JAMA Network Open.*

Diagnosis	ICD Version	ICD Code	Number of Encounters
Trauma	9	80350	3
Trauma	9	9038	5
Trauma	9	80069	2
Trauma	9	80305	1
Trauma	9	80631	2
Trauma	9	94147	3
Trauma	9	84219	1
Trauma	9	80190	4
Trauma	9	8406	1
Trauma	9	8703	11
Trauma	9	88021	2
Trauma	9	9099	1
Trauma	9	9585	2
Trauma	9	9056	1
Trauma	9	8081	3
Trauma	9	9089	3
Trauma	9	86346	3
Trauma	9	87333	2
Trauma	9	86130	3
Trauma	9	9143	5

© 2021 Pelletier et al. *JAMA Network Open.*

Diagnosis	ICD Version	ICD Code	Number of Encounters
Trauma	9	94106	4
Trauma	9	8409	7
Trauma	9	94448	1
Trauma	9	80436	1
Trauma	9	80196	3
Trauma	9	94345	1
Trauma	9	90454	1
Trauma	9	85124	1
Trauma	9	90182	2
Trauma	9	80395	2
Trauma	9	81407	1
Trauma	9	9532	1
Trauma	9	85173	1
Trauma	9	87400	1
Trauma	9	80711	2
Trauma	9	9011	5
Trauma	9	80635	1
Trauma	9	8073	1
Trauma	9	95207	1
Trauma	9	80381	1

© 2021 Pelletier et al. *JAMA Network Open.*

Diagnosis	ICD Version	ICD Code	Number of Encounters
Trauma	9	8090	1
Trauma	9	9034	1
Trauma	9	9063	4
Trauma	9	82012	1
Trauma	9	85155	1
Trauma	9	80391	1
Trauma	9	9085	1
Trauma	9	82530	1
Trauma	9	9093	8
Trauma	9	9071	1
Trauma	9	9080	1
Trauma	9	9299	1
Trauma	9	85176	2
Trauma	9	8942	2
Trauma	9	80712	3
Trauma	9	86380	1
Trauma	9	90233	2
Trauma	9	80173	3
Trauma	9	84512	1
Trauma	9	9391	1

© 2021 Pelletier et al. *JAMA Network Open.*

Diagnosis	ICD Version	ICD Code	Number of Encounters
Trauma	9	80013	4
Trauma	9	9058	2
Trauma	9	9013	1
Trauma	9	80005	3
Trauma	9	8673	4
Trauma	9	80323	3
Trauma	9	9580	1
Trauma	9	9531	1
Trauma	9	83660	1
Trauma	9	85143	2
Trauma	9	8785	11
Trauma	9	95217	2
Trauma	9	9252	5
Trauma	9	83815	1
Trauma	9	84213	2
Trauma	9	94402	1
Trauma	9	9101	7
Trauma	9	80169	1
Trauma	9	86232	2
Trauma	9	9153	5

© 2021 Pelletier et al. *JAMA Network Open.*

Diagnosis	ICD Version	ICD Code	Number of Encounters
Trauma	9	87331	1
Trauma	9	80179	2
Trauma	9	94500	3
Trauma	9	80852	1
Trauma	9	88029	1
Trauma	9	80049	2
Trauma	9	80512	1
Trauma	9	90181	1
Trauma	9	82030	2
Trauma	9	86102	1
Trauma	9	94326	1
Trauma	9	81402	2
Trauma	9	85232	1
Trauma	9	9243	3
Trauma	9	9301	3
Trauma	9	86411	6
Trauma	9	9192	1
Trauma	9	83211	3
Trauma	9	8963	1
Trauma	9	9403	1

© 2021 Pelletier et al. *JAMA Network Open.*

Diagnosis	ICD Version	ICD Code	Number of Encounters
Trauma	9	83304	3
Trauma	9	92301	2
Trauma	9	9558	1
Trauma	9	81380	5
Trauma	9	81350	2
Trauma	9	9578	3
Trauma	9	8053	3
Trauma	9	81013	1
Trauma	9	82391	3
Trauma	9	80486	1
Trauma	9	80634	2
Trauma	9	83654	1
Trauma	9	9052	2
Trauma	9	8209	4
Trauma	9	9119	1
Trauma	9	85211	6
Trauma	9	80630	1
Trauma	9	85410	1
Trauma	9	80073	3
Trauma	9	85131	4

© 2021 Pelletier et al. *JAMA Network Open.*

Diagnosis	ICD Version	ICD Code	Number of Encounters
Trauma	9	9561	1
Trauma	9	83805	1
Trauma	9	94860	1
Trauma	9	94150	1
Trauma	9	85245	1
Trauma	9	80339	1
Trauma	9	80854	1
Trauma	9	85192	1
Trauma	9	9053	1
Trauma	9	80192	1
Trauma	9	9082	1
Trauma	9	9069	1
Trauma	9	9139	1
Trauma	9	84209	2
Trauma	9	9540	3
Trauma	9	81330	3
Trauma	9	90302	1
Trauma	9	9512	2
Trauma	9	85412	1
Trauma	9	85161	1

© 2021 Pelletier et al. *JAMA Network Open.*

Diagnosis	ICD Version	ICD Code	Number of Encounters
Trauma	9	81603	2
Trauma	9	80440	2
Trauma	9	84502	1
Trauma	9	80402	1
Trauma	9	9461	1
Trauma	9	80471	2
Trauma	9	85243	1
Trauma	9	9158	2
Trauma	9	9-E8845	1
Trauma	9	95890	1
Trauma	9	83511	1
Trauma	9	80092	1
Trauma	9	80183	1
Trauma	9	94201	1
Trauma	9	86385	3
Trauma	9	9182	3
Trauma	9	90210	2
Trauma	9	80352	1
Trauma	9	95212	1
Trauma	9	94414	1

© 2021 Pelletier et al. *JAMA Network Open.*

Diagnosis	ICD Version	ICD Code	Number of Encounters
Trauma	9	81346	2
Trauma	9	94811	11
Trauma	9	83305	2
Trauma	9	94850	1
Trauma	9	80089	1
Trauma	9	9513	2
Trauma	9	85194	1
Trauma	9	85231	3
Trauma	9	83806	2
Trauma	9	94844	2
Trauma	9	80639	1
Trauma	9	90141	1
Trauma	9	8411	1
Trauma	9	94313	2
Trauma	9	94413	1
Trauma	9	80401	1
Trauma	9	81400	2
Trauma	9	81113	1
Trauma	9	80361	1
Trauma	9	85244	2

© 2021 Pelletier et al. *JAMA Network Open.*

Diagnosis	ICD Version	ICD Code	Number of Encounters
Trauma	9	84841	1
Trauma	9	8055	1
Trauma	9	8503	3
Trauma	9	80164	2
Trauma	9	80114	6
Trauma	9	83201	2
Trauma	9	83402	3
Trauma	9	80105	2
Trauma	9	85303	2
Trauma	9	80044	1
Trauma	9	80084	1
Trauma	9	80195	2
Trauma	9	90220	1
Trauma	9	90242	1
Trauma	9	81411	1
Trauma	9	9523	1
Trauma	9	83309	1
Trauma	9	92309	2
Trauma	9	9238	1
Trauma	9	8874	1

© 2021 Pelletier et al. *JAMA Network Open.*

Diagnosis	ICD Version	ICD Code	Number of Encounters
Trauma	9	80409	1
Trauma	9	80472	1
Trauma	9	9474	1
Trauma	9	9220	1
Trauma	9	8677	2
Trauma	9	80340	1
Trauma	9	9125	1
Trauma	9	8271	1
Trauma	9	81320	1
Trauma	9	80233	1
Trauma	9	83510	1
Trauma	9	81410	1
Trauma	9	9518	2
Trauma	9	8674	1
Trauma	9	94442	1
Trauma	9	94148	2
Trauma	9	80324	1
Trauma	9	94830	1
Trauma	9	94509	1
Trauma	9	80517	1

© 2021 Pelletier et al. *JAMA Network Open.*

Diagnosis	ICD Version	ICD Code	Number of Encounters
Trauma	9	94108	2
Trauma	9	94408	2
Trauma	9	80660	1
Trauma	9	85216	1
Trauma	9	90000	1
Trauma	9	95202	1
Trauma	9	80619	1
Trauma	9	85312	1
Trauma	9	94111	2
Trauma	9	8089	1
Trauma	9	80033	1
Trauma	9	9129	1
Trauma	9	9189	1
Trauma	9	8403	1
Trauma	9	8404	2
Trauma	9	90231	1
Trauma	9	83400	1
Trauma	9	85109	1
Trauma	9	80182	1
Trauma	9	83513	1

© 2021 Pelletier et al. *JAMA Network Open.*

eTable 2. Number of Encounters With Included Diagnoses

Diagnosis	Number of Encounters (%)
Appendicitis	113,394 (2.09%)
Atrial Septal Defect	10,787 (0.2%)
Asthma	203,486 (3.75%)
Birth	229,059 (4.22%)
Bronchiolitis	228,637 (4.21%)
Cardiac arrest	1,557 (0.03%)
Coarctation of the Aorta	9,899 (0.18%)
Dehydration	63,291 (1.17%)
Diabetic ketoacidosis	65,688 (1.21%)
Hypoplastic Left Heart Syndrome	11,083 (0.2%)
Kawasaki syndrome	16,653 (0.31%)
Mental health	190,355 (3.51%)
S. pneumoniae	2,818 (0.05%)
Sepsis	64,245 (1.18%)
Tetralogy of Fallot	13,549 (0.25%)
Trauma	221,875 (4.09%)

Note: the percentage shown is a percentage of all PHIS inpatient encounters.

© 2021 Pelletier et al. *JAMA Network Open.*
